# Supplementary material for: Enhancing fieldworkers’ performance management support in health research: an exploratory study on the views of field managers and fieldworkers from major research centres in Africa
Source: BMJ Open. 2019 Dec 18;9(12):e028453. doi: 10.1136/bmjopen-2018-028453 (PMC6937094; doi:10.1136/bmjopen-2018-028453)
Supplement: Supplementary data [file bmjopen-2018-028453supp001.pdf]

DISCUSSION GUIDE FOR RESEARCH INSTITUTIONS WORKING WITH FIELDWORKERS (FWs) IN SSA

TITLE: STRENGTHENING TRAINING OF FRONT LINE (FWs) STAFF AS A MEANS TO ENHANCE COMMUNITY ENGAGEMENT

INFORMATION SHEET FOR MANAGERS AND SENIOR FRONTLINE STAFF

What is this survey all about?

This survey will be conducted through telephone/ teleconference discussion. One front line staff manager (people who have a direct role of providing support supervision to frontline staff) and one senior frontline staff member from a total of 20 health research institutions in Sub-Saharan Africa will be invited to participate in the discussion.

The aim of the discussion is to identify practical and ethical challenges experienced by frontline staff in their day to day activities and the available support supervision, performance management and Continuing Professional Development (CPD) practices within these institutions. Later, representatives from a few of the 20 institutions will be invited to a consultative workshop to follow up and discuss these issues in detail. The report generated from the survey and workshop will be shared with all institutes for their own use. In the long term, the report could be used to inform further discussions on the potential of developing a generic curriculum for frontline staff training.

What is expected of you if you take part in this discussion?

If you agree to take part in this discussion, we will request you to select a date and time when you would prefer the moderator to call and have the discussion with you. We will then call and ask a few questions with regards to your perceptions of the roles of frontline staff and the institutional support supervision, monitoring and evaluation and general CPD practices. These questions are shown on the attached discussion guide for your attention. The discussion is expected to take a maximum of 30 minutes.

Participation in this interview is voluntary. While it is important to answer all the questions, feel free to skip any question that you do not feel comfortable to answer and the moderator will continue asking the next question. You can also decide to terminate the discussion at any time if you decide not to continue with the discussion for any reason.

Is there any individual benefit for taking part in this discussion?

There is no individual benefit in taking part in this discussion. However, by sharing your experiences in working with frontline staff, you will assist in contributing to knowledge that could improve the way frontline staff deal with their practical and ethical challenges as well as their support supervision, performance and general CPD management practices in future.

If you have any question

If you have any question about this survey, please contact any of the following people through the email addresses provided below

Francis Kombe<br>KWTRP<br>P.O BOX 230, KILIFI<br>Fkombe@kemri-wellcome.org	Sam Kinyanjui<br>KWTRP<br>P.O BOX 230, KILIFI<br>Skinyajui@kemri-wellcome.org	Dorcas Kamuya<br>KWTRP/ Ethox-Nuffield Department of Population Science, Oxford University<br>P.O BOX 230, KILIFI<br>Dkamuya@kemri-wellcome.org

DISCUSSION GUIDE FOR RESEARCH INSTITUTIONS WORKING WITH FIELDWORKERS (FWs) IN SSA

TITLE: STRENGTHENING TRAINING OF FRONT LINE (FWs) STAFF AS A MEANS TO ENHANCE COMMUNITY ENGAGEMENT

SECTION A: Institutional Background

1. Name of organization ____
2. Vision ____
3. Mission ____
4. Motto ______________________________________
5. Year organization was established [__][__][__] [__]
6. Source of main funding for the organization ______________________________________
7. Other funding agencies and collaborators ______________________________________
8. Types of research conducted at the organization: ___________________________________

SECTION B: Frontline staff Managers' Perspective[1]

This section explores managers' perceptions of the roles of and practical and ethical challenges faced by interface staff when discharging their duties and the institutional support supervision, performance management and Continuing Professional Development (CPD) practices

1. What names are given to your frontline staff whose primary role involves recruiting and consenting research participants into studies?
2. What key roles/activities do they undertake in your organization?
3. In your institution, how many staff does it have (in total) and how many of these are employed as frontline staff?
4. What is the range of qualifications for frontline staff and what is their minimum level of education/qualification?
5. What is the nature of their employment e.g. are they in contract? What is the average contract period? Are the contracts renewable and what is considered during renewal? Any systems that ensure retention of frontline staff in the institutions?
6. What systems are in place to coordinate the way frontline staff are employed and retained across different studies in the institutions? What is the role of PI (if any) in retaining frontline staff?
7. What formal and informal processes are in place to induct new frontline staff when they first arrive, and to train them for their roles?

[1] Manager is the person whose primary responsibility involves working directly with front line staff

DISCUSSION GUIDE FOR RESEARCH INSTITUTIONS WORKING WITH FIELDWORKERS (FWs) IN SSA

TITLE: STRENGTHENING TRAINING OF FRONT LINE (FWs) STAFF AS A MEANS TO ENHANCE COMMUNITY ENGAGEMENT

8. How is the training for interface staff organized? Are there any refresher trainings/skills improvement activities? What are the *training approach eg frequency and objectives of the training,*

9. How is the frontline staff training managed? Describe funding, design, whether centralized or decentralized, and monitoring and evaluation of the training

10. Describe any practices, experiences and studies (if any) that have informed the current training eg feedback mechanisms; availability of a community engagement strategy, community Knowledge Attitude and Perception (KAP) surveys etc

11. How do you ensure skills learned are assimilated into the frontline's day to day activities? Are there any support supervision mechanisms, performance appraisals etc ? How are these implemented?

12. Describe any career progression scheme used by your organization for these staff groups

13. What challenges do you experience in working with and managing frontline staff?

SECTION C: Frontline staffs' Experience-Staff perspective

This section explores frontline staff's practical and ethical challenges faced as they discharge their duties and their perceptions of the institutional support in training and CPD

1. What are roles and responsibilities of frontline staff?

2. What challenges do frontline staff face when interacting with community members? *Practical and ethical challenges/dilemmas faced, e.g. issues of confidentiality?*

3. What type of in-house and external training is given to frontline staff in your institution?
    - ***Which of these do you perceive to be most appreciated and for what reason?***

4. What are the perceived institutional challenges in supporting and capacity building of frontline staff?
    - ***How do frontline members of staff address the above challenges?***

SECTION D: Planned Workshop

At the end of this survey, we hope to invite a selected number of the institutions to a 2 day workshop. Kindly indicate if you would like to be considered to attend this workshop.
