# Supplementary material for: Enhancing fieldworkers’ performance management support in health research: an exploratory study on the views of field managers and fieldworkers from major research centres in Africa
Source: BMJ Open. 2019 Dec 18;9(12):e028453. doi: 10.1136/bmjopen-2018-028453 (PMC6937094; doi:10.1136/bmjopen-2018-028453)
Supplement: Author's manuscript [file bmjopen-2018-028453.draft_revisions.pdf]

BMJ Open is committed to open peer review. As part of this commitment we make the peer review history of every article we publish publicly available.

When an article is published we post the peer reviewers' comments and the authors' responses online. We also post the versions of the paper that were used during peer review. These are the versions that the peer review comments apply to.

The versions of the paper that follow are the versions that were submitted during the peer review process. They are not the versions of record or the final published versions. They should not be cited or distributed as the published version of this manuscript.

BMJ Open is an open access journal and the full, final, typeset and author-corrected version of record of the manuscript is available on our site with no access controls, subscription charges or pay-per-view fees (http://bmjopen.bmj.com).

If you have any questions on BMJ Open's open peer review process please email info.bmjopen@bmj.com

BMJ Open

# Enhancing Fieldworkers' Performance Management Support in Health Research: Views of Field managers and Fieldworkers from major research centres in Africa

| | |
|---|---|
| Journal: | *BMJ Open* |
| Manuscript ID | bmjopen-2018-028453 |
| Article Type: | Research |
| Date Submitted by the Author: | 11-Dec-2018 |
| Complete List of Authors: | Kombe, Francis; Centre for Geographic Medicine Research Coast; African Research Integrity Network<br>Marsh, V; Centre for Geographic Medicine Research Coast; Centre for Tropical Medicine and Global Health, Nuffield Department of Medicine, Oxford University, UK<br>Molyneux, Sassy ; Centre for Geographic Medicine Research Coast; Centre for Tropical Medicine and Global Health, Nuffield Department of Medicine, Oxford University, UK<br>Kamuya, Dorcas; KEMRI Wellcome Trust Research Programme; Ethox Centre, Nuffield Department of Population Health, Oxford University, UK<br>Ikamba, Dorothy; KEMRI Wellcome Trust Research Programme<br>Kinyanjui, Samson; Centre for Geographic Medicine Research Coast; Centre for Tropical Medicine and Global Health, Nuffield Department of Medicine, Oxford University, UK |
| Keywords: | Fieldworkers, Africa, Capacity Building, Health Research, Research Ethics |
| | |

SCHOLARONE™
Manuscripts

# Enhancing Fieldworkers' Performance Management Support in Health Research: *Views of Field managers and Fieldworkers from major research centres in Africa*

Francis Kombe[1], Vicki Marsh[1,3], Sassy Molyneux[1,3], Dorcas Kamuya[1,2], Dorothy Ikamba[1], Sam Kinyanjui[1,3]

[1]KEMRI Wellcome Trust Research Programme, Kenya; [2]Ethox Centre, Nuffield Department of Population Health, Oxford University, UK; [3]Centre for Tropical Medicine and Global Health, Nuffield Department of Medicine, Oxford University, UK.

**Corresponding author**: Francis Kombe, KEMRI Wellcome Trust Research Programme, PO Box 230, Kilifi 80108, Kenya. Mobile +254 722459045; Email-Kajoleh@gmail.com

**Acknowledgements**: We acknowledge and greatly appreciate the support of all research institutions' directors who allowed their field managers and field workers to take part in this project. We thank all the field managers and field workers who agreed to participate in this project. We are extremely indebted to Prof Mike Parker, the Director of the Wellcome Centre for Ethics and Humanities and Director of Ethox Centre at the University of Oxford for his support and guidance during the project; and all the senior field managers and delegate who attended the consultative workshop. The manuscript is published with the support of the Director, KEMRI.

**Funding**:
This project was funded by Global Health Bioethics Network-UK (Wellcome Trust grant no. OXF-GHB02) and supplemented by KWTRP Capacity Building Strategic Award (Wellcome Trust grant no 084538). The research and capacity building activities of the Global Health Bioethics Network are supported by a Wellcome Trust Strategic Award (096527)

**Keywords:** Africa, Fieldworkers, Integrity, Research, Ethics, Capacity.

For peer review only

**Abstract**

***Introduction***

Research frontline staff; popularly known as fieldworkers, play a critical role in promoting scientific and ethical standards through data collection and seeking consent. The credibility and integrity of community-based research, therefore, rely heavily on how this cadre of staff adheres to institutional and research procedures and guidelines.

**Study objectives**

This study mapped out existing practices in relation to training, support and performance management of fieldworkers in sub-Saharan Africa and described experiences and lessons learned in the process of managing fieldworkers.

**Methods**

In this qualitative study, we conducted a total of 32 telephone interviews involving field managers (n=20) and senior fieldworkers (n=12) from 18 major research Centres in Africa, MRC-UK and the INDEPTH Network Secretariat. In the interviews, we explored institutional performance management practices, and fieldworker challenges and support processes. Subsequently, out of the 32 telephone interviews held, 25 participants comprising of field managers, senior social scientists with long-term experience of working with fieldworkers, training experts and an independent consultant with experience in group moderation and consensus building techniques, were invited to attend a deliberative workshop. Data from the interviews and workshop proceedings were analyzed thematically using Nvivo 10 software.

**Results**

Most institutions employed fieldworkers, usually with 12 years of formal education and residing within the community, to support studies. Although their roles were common, there were marked differences in the type of training, continuous professional development schemes and other support fieldworkers received across the research centres. Fieldworkers faced various challenges, including physical burdens of fieldwork, emotional burdens, lack of structured training, lack of career development pathways and insecure employment contract, which had the potential to affect their ethical and scientific practices.

**Conclusion**

We argue that there is a need for research institutions to enhance structured support, supervision, soft skills training for fieldworkers.



For peer review only - http://bmjopen.bmj.com/site/about/guidelines.xhtml

**Strengths and limitations of this study**

- The study involved a wide range of institutions with various experiences and practices in managing fieldworkers. It therefore provides a good overview of how research institutions in Africa manage fieldworkers
- Selection of the research institutions that were involved in the study was based on their relationship with the INDEPTH Network and the training department at the KWTRP. This selection is not representative of research institutions that employ fieldworkers in Africa
- The interviews were conducted through skype and telephone. As such, it was not possible to capture important interview dynamics, including facial expressions and other non-verbal commination which communicate important participants' reactions.

## BACKGROUND

High-quality data is a critical output of all science worldwide. Thus, research institutions put significant emphasis on generating data that are of the highest scientific and ethical standards. In many research programmes in low and middle-income countries, data collection is often done by frontline staff who are commonly referred to as "fieldworkers". This cadre of staff is also responsible for recruiting potential research participants, administering consent forms and following up participants during studies (Kamuya et al., 2013). As such, beyond ensuring data integrity, fieldworkers play a key role in ensuring the ethical implementation of research protocols. The role of fieldworkers is even more critical in international collaborative programmes which often include researchers drawn from outside the local communities. In such settings, fieldworkers, who are normally from the local communities, serve as the face of the research programmes and therefore have the potential to significantly influence the communities' trust in the programmes and research in general.

In carrying out these complex roles, fieldworkers often face practical and ethical dilemmas that require the balancing of institutional roles and guidelines against community expectations and fieldworkers' personal interests. Hence, fieldworkers continuously make independent (often unconscious) decisions on how to apply 'ethical principles' in reality. For example fieldworkers reported challenges such as dealing with silent refusals (Kamuya et al., 2015)-participants who may not openly refuse to participate in research but indirectly avoid key study procedures, dealing with increasing demands for study benefits from community members who may or not be study participants and dealing with request for health and other social- economic support from study participants that are beyond the scope of the respective study (Chantler et al., 2013; Kingori, 2013; Madiega, Jones, Prince, & Geissler, 2013; Marshall & Marshall, 2007)



For peer review only - http://bmjopen.bmj.com/site/about/guidelines.xhtml

Over the years there has been an increasing interest and investment in developing research capacity in Africa and other low and middle income regions through long-term initiatives such as the WHO-Tropical Diseases Research Programme (TDR) (Lansang & Dennis, 2004), the European and Developing Countries Clinical Trials Initiative Partnership (EDCTP) and more recently, The Wellcome Trust-DFID funded Developing Excellence in Leadership, Training and Science scheme (DELTAS) Africa. Efforts to strengthen research capacity are likely to continue, as the trend towards greater cross-border data sharing, globalization of clinical research and comparability of data quality at the global level continues (Glickman et al., 2009).

Given that fieldworkers play such a vital role in health research, it would be expected that research programmes, in addition to training scientists, would also focus on building fieldworkers' capacity to enable them to carry out their duties in the most effective manner. However, fieldworkers' capacity and skill sets, have received relatively little attention. The mechanisms and processes by which research institutions in Africa support fieldworkers in their execution of duties remain unclear. Apart from the Good Clinical Practice guidelines, which focus primarily on clinical trials, no other guidelines are available that provide a framework for the content and approach for supporting the Continuing Professional Development and career growth of fieldworkers.

Understanding the challenges that fieldworkers face and the type of systems, and level of support research institutions in Africa provide can enable us to identify opportunities for strengthening fieldworkers' scientific and ethical practices.

In this paper, we draw on our findings from a two-phase qualitative study on the perceptions, experiences and views of field managers and fieldworkers on the structural and performance management support extended to health research fieldworkers with the aim of informing institutional policies that promote research integrity and ethical practices for this critical cadre of staff (Participants of an International Workshop in Kenya on the Role of Frontline Staff in Biomedical Research & Kombe, 2015).

## METHODS

The study was planned over two phases, focused on understanding structural and performance management practices and their implications within institutions. In Phase one, we conducted semi-structured telephone interviews with 32 fieldworker managers and experienced fieldworkers across 20 health research institutions in 14 countries in Africa and the United Kingdom. In Phase two, we held a two-day workshop including 25 fieldworker managers from the group that participated in Phase one, and a number of additional stakeholders, as described in the following sections.



For peer review only - http://bmjopen.bmj.com/site/about/guidelines.xhtml

**Study site and selection of research institutions.**

This study was led by a team based at the Kenya Medical Research Institute (KEMRI) Wellcome Trust Research Programme (KWTRP) in Kenya. The KWTRP is a long-standing international health research institution with a wide range of existing scientific and capacity building collaborations with other research institutes in sub-Saharan Africa (see http://kemri-wellcome.org/). During the first phase, our sampling strategy for research institutions, and relevant individuals within these focused on African institutions that manage Health and Demographic Surveillance Systems as the group that would consistently employ significant numbers of fieldworkers. We identified these institutions from our existing scientific and capacity building linkages, and from information from the INDEPTH Network ( http://www.indepth-network.org/), an international research network for health and demographic surveillance sites in Africa. From the original list of 34 institutions obtained from the KWTP training department and INDEPTH site, we sent an email to the directors with an information sheet about the study and requested them to identify one senior fieldworker and field manager from their institution, who were able to talk to us about the study. A total of eighteen African international research institutions (see table 1) and the MRC-UK and the INDEPTH Network Secretariat in Ghana responded positively and hence participated in our discussions. The eighteen African research institutions included a mix of institutions with varied years of operations, employed a diverse number of fieldworkers, engaged in a range of local and international collaborations and specialized in different research programmes/thematic areas. This mixture of characteristics was important in maximizing shared learning and experiences across institutions. In each institution, we wrote to the director and sought permission to interview one senior fieldworker and one field manager. In the letter, we particularly underscored the need to select a manager whose primary responsibility involved working directly with and managing fieldworkers, including offering support supervision, training and other forms of performance management.

**Data collection and analysis**

***Phase 1: Telephone Interviews with managers and fieldworkers***

We contacted all the 34 institutions, whose contacts were obtained from KWTRP's collaborators' database and the INDEPTH Network website. Of these, 53% replied (n=18) replied positively, with names of two individuals (1 senior fieldworker and 1 field manager) to be interviewed. With permission from the institution's directors, we directly contacted the proposed individuals and sought their individual consent to participate in a semi-structured interview, using telephone or Voice Over Internet Protocol (VOIP) communication. In total, the 18 institutions that participated in the study employed a total workforce of 7494 staff, with 2105 (28.1%) being fieldworkers. Table 1 shows all the



For peer review only - http://bmjopen.bmj.com/site/about/guidelines.xhtml

institutions that participated in the study, marked in dark blue (those that attended the workshop) and light blue (those that did not attend the workshop), with their names anonymized. In total, 7 Research Institution from West Africa, 4 Research Institution from Southern Africa and 7 Research Institution from East Africa participated in the interviews. In addition, 1 regional research coordination institution from Africa and 1 UK research funding organization were also interviewed.

Overall, from these interviews, we sought to develop an understanding of current institutional practices and experiences in relation to training, support supervision and performance management of fieldworkers. Interviews with field managers explored issues related to processes of recruiting fieldworkers, the minimum qualification for fieldworkers, and the requirements for professional training as well training given to fieldworkers. Additionally, issues around capacity building, support supervision and professional development for fieldworkers were discussed. Interviews with senior fieldworkers focused on their perceptions of the roles they play in research, challenges and mechanisms for addressing these challenges, career development opportunities and other existing support systems. The first author conducted the interviews, which were digitally voice recorded, transcribed and immediately read through for verification and filling of any gaps by the interviewer before analysis.

Data analysis in this study followed a Framework Analysis approach (Srivastava & Thomson, 2009). Transcripts were independently read and discussed in-depth by two members of the study team (FK and VM) to develop an initial coding framework, drawing on the main questions raised during interviews and themes that emerged during discussions. FK used Nvivo 10 software to organize data across the transcripts under these codes, adding codes for new issues emerging from the data. Data analysis charts were developed by FK and VM to account for the codes under themes by the participants, and support interpretation of patterns within the responses, drawing on the social science and research ethics literature.

***The workshop with field managers***

A total of 25 managers from fifteen of the eighteen research Centres that took part in the phase one telephone interviews attended the 2 ½ days' workshop to further discuss the phase one findings. In total, the fifteen institutions represented at the workshop employed a total of 6702 staff, with 1726 (25.9%) of them being fieldworkers. The institutions came from nine different African countries. To attend the workshop, invitations were sent directly to the individuals who participated in phase one interviews and requested to seek clearance from their line managers before attending the workshop.

The workshop agenda was developed collaboratively between the study team and an independent Kenyan consultant who acted as the main workshop facilitator, and who had been selected based on



For peer review only - http://bmjopen.bmj.com/site/about/guidelines.xhtml

his in-depth experience of participatory workshop methods and prior familiarity with research institutions in Africa. Overall, the workshop combined information sharing and large group discussions on day one with a series of participatory deliberative and consensus building activities over the following days. On day one, two sets of presentations were made and discussed in plenary. The first presentation described Phase one findings, with discussions on the range, strengths and challenges of fieldworkers' employment and support structures across institutions. The second presentation highlighted findings from earlier social science research in KWTRP and other research sites in Africa on the complex nature of fieldwork, including practical, social and ethical challenges that fieldworkers often face as part of their routine work, particularly within low-income communities and householders. Discussions that followed the presentations focused on the relevance and importance of these challenges across sites.

**Patients and Public Involvement**

This study did not include patients or/and public involvement. However, some of the participants who participated in the telephone interviews attended the consultative workshop where results from the telephone interviews were presented. During the workshop, the participants who were involved had an opportunity to review the telephone interview findings and share their comments.

**FINDINGS AND DISCUSSION**

In this section, we describe and discuss findings from the telephone survey and the subsequent workshop together across three inter-related themes that were explored or emerged as important across this study. These themes are A) The current institutional structures and systems that support fieldworkers across institutions involved in the study; B) Participants' perceptions of work-related challenges for field workers and their relationship to support structures and; C) Views on the ways in which challenges for fieldworkers might be better addressed, including agreement on institutional and wider recommendations.

**A. Current Institutional structures and systems for fieldworkers**

Across interviews and the subsequent workshop, it became clear that there is a large amount of diversity in the names, roles and institutional structures that govern and support the way that fieldworkers operate in the institutions involved. For example, during interviews, senior fieldworkers described the existence of many different roles and titles, including fieldworker, field enumerator, field officer, tracer, community technician and field assistant, which were often used interchangeably, within and across different centres. Similarly, the way that fieldworkers were recruited, contracted, trained and supported in their work was highly variable, again, between projects within an institution



For peer review only - http://bmjopen.bmj.com/site/about/guidelines.xhtml

and between institutions. Table 1 describes these parameters across the research institutions involved.

As Table 1 shows, some form of in-house fieldworker training was common across institutions, with training mainly focused on the skills needed to undertake study-specific tasks, such as using particular data collection tools. In some cases, training in Good Clinical Practice (GCP) was also offered, particularly to support fieldworkers undertaking informed consent processes. This training was generally the responsibility of individual study teams within institutions. Four out of the fifteen institutions confirmed having a centrally coordinated training approach, three of which covered a more comprehensive set of topics including data collection skills, communication skills, informed consent processes, GCP and research ethics more generally. The other sites described conducting some kind of ad hoc training based on a specific study requirement or had no training plans for fieldworkers.

One area of fieldworker performance management which was rarely discussed and generally under-recognized across institutions was the concept of fieldworkers' professional career progression or continuing professional development. While such measures were largely routine for other cadres of research staff within institutions, they had not been applied to fieldworkers. Many felt this would be an important step towards recognizing the important role fieldworkers play, and in motivating them to maintain a high professional standard of work, but challenges were seen in sustaining such an initiative given the typically short-term contracts offered to fieldworkers, based on similarly short research funding cycles.

### B. Participants' perceptions of work-related challenges for fieldworkers and their relationship to support structures

Across the following paragraphs, we describe the forms of challenges that fieldworkers and those working closely with them described during interviews, and that were taken forward for discussion at the workshop. In describing these, we highlight the types of institutional support that influence these challenges, including structural influences from human resource management strategies and research funding cycles, and more *ad hoc* often individual efforts to manage complex challenges on the ground.

#### i) ***Using different 'labels' for fieldworkers***

Discussions during interviews and at the workshop drew out differences in opinions on the importance of the 'labels' that were used to describe the work we describe here as 'fieldwork'. At a most pragmatic level, some fieldworkers felt that the most important issue was having a job, rather than being concerned about the title that was associated with that job. Practically, this attitude seems to



For peer review only - http://bmjopen.bmj.com/site/about/guidelines.xhtml

hint at potentially problematic power imbalances for fieldworkers in relation to the institutions in which they were employed. Other fieldworkers felt that using different 'labels', particularly within one institution, could generate confusion about roles and lead to employment conditions being less transparent:

> *"It [roles taken by field workers] is not different. These are the organisation's terms…community technician, field worker, follow up staff, field technician…I mean it is just the same thing…it's just their [the organisation] way of making us look different, but we are the same"*. (SFW, KN)

Many field managers recognised the phenomenon of multiple labels as being used for slightly different forms of 'fieldwork'. There was, however, an initial divergence of opinion about the value of harmonising job titles. Some felt that this variation underlined important, even if small differences, in fieldworkers' current roles and prior experience or qualifications, which might not be obvious to all team members. Others felt there was little need for multiple titles, given the potential for confusion and conflict this might present. As will be described in the later section on recommendations, developing clear and strategic approaches to fieldworkers' roles within an institution emerged as an important feature of good practice for fieldworkers employment. For many, harmonizing these structures and processes across institutions was also seen as valuable. Field managers felt harmonizing fieldworker employment terms, training and skills would promote cross-study and institution transfer of fieldworkers and address the challenge associated with high turnover of fieldworkers due to short-term contracts

ii) ***Seeing fieldwork as a short-term activity***

A core tension for fieldworkers that was strongly expressed in interviews and the workshop was a general lack of recognition for the work that fieldworkers undertake within research institutions. Fieldworkers themselves saw their work as important, and as fundamentally contributing to answering important research questions and the reputation of institutions and careers of scientists and other cadres of research staff. There was, however, some frustration and perceived unfairness around the lack of opportunities for fieldworkers to progress within their area of work, or achieve recognition over sometimes periods of many years working in this role. Experiences were shared in which scientists who joined research centres were able to move ahead to higher positions through focused capacity building strategies, while fieldworkers' positions remained static. Some fieldworkers described working for an institution for more than ten years without more than basic training or promotion. Perhaps not surprisingly, given the way that 'fieldwork' has traditionally been viewed within research institutions (that is, as short-term and relatively non-technical work), the institutions involved in this study had not developed strategic approaches to fieldworkers' career development.



For peer review only - http://bmjopen.bmj.com/site/about/guidelines.xhtml

In response to these frustrations, some fieldworkers had taken up self-funded part-time training courses outside their existing jobs in the hope that this would allow them to progress within the institution. In practice, however, institutions did not have structures that would allow fieldworkers to move into roles in which any such new skills could be used. Against this background, most fieldworkers reported feeling demoralized and demotivated, including a feeling of low self-esteem and disillusionment:

> *"Once you are employed as a fieldworker, you will always be a fieldworker* [regardless of your qualifications]*". (SFW, GN)*

Given these challenges, which workshop participants saw as fundamentally unfair and as potentially importantly undermining the quality of research at its roots, participants agreed that fieldworkers should have greater recognition for the role they play within research institutions. They also agreed that it was important for institutions to support some form of career progression, for example, within a specialised category of staff supporting fieldwork. Providing fieldworkers with generic knowledge and skills on core research areas as part of such a scheme would enable them to transfer between studies at the end of a funding cycle. This would enable research centres to retain experienced fieldworkers, a policy that was thought likely to promote high scientific and ethical standards; and give fieldworkers more stability, skills and recognition.

### iii) **Responding to complex challenges on the ground**

Across this study, both during interviews and the subsequent workshop, fieldworkers and managers described often very difficult challenges that seem to be implicit in the nature of much field work. Some of these were practical, including the physical burdens of fieldwork, such as the experience of long working days with long distances to cover on foot, and risks of injury through dog or snake bites, and physical assault in less secure settings. In addition, many described emotional burdens, including anxiety, fear, sadness and sometimes helplessness associated with the nature of their work. This latter form of burden was described as being part of the nature of fieldwork, given common interactions with individuals and families who are facing often severe social and economic hardship. Within the community, fieldworkers were described as being seen as 'fellow community members' and, at the same time, the 'face' of the institutions that employ them, generating expectations of support from them on the part of community members visited (Molyneux et al., 2013)

These challenges have been well described in the literature on global health ethics, including from social science research at KWTRP, and our findings in this study underline the existence of these important challenges (Angwenyi et al., 2013; Chantler et al., 2013; Kamuya et al., 2013; Kingori, 2013;



For peer review only - http://bmjopen.bmj.com/site/about/guidelines.xhtml

Molyneux et al., 2013). The fact that our study draws on experiences from 18 major research Centres in Africa and found similar challenges is an indication of how common and important those challenges are. Within this literature, there is increasing recognition of the moral work that fieldworkers are involved in during their everyday work (Kamuya et al., 2013; Wax, 1977; Zinn, 1979) which can directly impact the science and ethics of the studies of which they are part (Kingori, 2013; Kombe et al., 2014). For example, a key issue in the literature concerns the negotiation of informed consent processes, where fieldworkers balance perceived pressures to recruit participants according to targets set by managers and the extended period of time that may be needed to explain complex and often unfamiliar topics within consent forms to householders; and community members may feel that by agreeing to participate in studies, they will develop some leverage to access greater institutional support, including through their relationships with fieldworkers (Chantler et al., 2013; Kingori, 2013; Simon & Mosavel, 2010)

A particularly difficult situation that fieldworkers involved in routine Demographic Surveillance System (DSS) activities in this study described was the need to directly interact with bereaved families and seek information about deceased relatives as part of conducting verbal autopsies (interviews held with close family members of a recently deceased person to try to establish the cause of death) (Fottrell & Byass, 2010; Zahr & Pinchuk, 2007) and other study-specific follow-ups. Such emotionally challenging interactions generated distress and worry for fieldworkers and could chronically undermine performance, leading to 'burnout':

> *"Yeah but you see what, after maybe going through these challenges sometimes they affect you or they affect your output"* [SFW, SA]
>
> *I felt bad, and you know the following day I was to go back to work, and there is no supportive counselling or something like that… So as fieldworkers sometimes we just burn out and sometimes with this kind of trauma, it becomes difficult…we go through a lot*" [SFW, KE]

During workshop discussions about support mechanisms to help fieldworkers address these emotional challenges, field managers and the fieldworkers were not able to identify existing strategies. Some managers described generally relying on fieldworkers' own personal judgements in handling these difficult situations. Experiences shared by fieldworkers revealed that the issues were usually quite complex and intertwined with cultural, structural and emotional sensitivities associated with the need to balance community and cultural expectation against the policies and guidelines of research institutions. As such, fieldworkers were often expected to strike a delicate balance, without knowledge of how acceptable their actions were. Despite these actions having the potential to directly affect the scientific and ethical standards of data fieldworkers collect, structural support and referral systems



For peer review only - http://bmjopen.bmj.com/site/about/guidelines.xhtml

were reportedly not available. This was well captured by one senior fieldworker who said. "…. *sometimes, you get that huruma [pity], you go into your pocket and give out something…if you have a human heart, sometimes there are some situations that are touching"*.

During interviews and workshop discussions, there were no accounts of existing internal or external institutional mechanisms to support fieldworkers to specifically deal with these types of emotional challenges. Although effective support supervision and sharing these challenges during team meeting was identified as a potential avenue to identify strategies to deal with these challenges, the approaches used to supervise fieldworkers were noted to be significantly different across institutions, and not centrally coordinated within institutions, making it difficult to reflect on their effectiveness. Workshop participants therefore generally felt there was a gap in fieldworkers' support structures that should be addressed. These challenges were seen as occurring across all the institutions involved in the study.

**C. Ways of strengthening institutional support**

Based on the outputs of discussions described so far, two core institutional fieldworker support strategies were raised, discussed and agreed upon over the course of the workshop. The first concerned adapting the *content of skill building initiatives* to support fieldworkers to manage their interactions with community members and householders in ways that could support good scientific and ethical practice, seen from multiple perspectives. The second strategic support mechanism addressed the wider question of how professional support within institutions could support fieldworkers more *structurally* towards these aims, including aspects of performance management that commonly exist for other groups of research staff. Workshop participants agreed that 'performance management' in this respect would include aspects of recruitment, training, supervision, support, assessment and career or professional development. Specifically, delegates emphasized the need to strengthen fieldworkers' soft skills training to ensure they are well equipped to deal with cross-cutting issues around communication, community engagement, building trust and respect, which are fundamental in consenting and quality data collection. The need to develop a harmonized training curriculum, with core training modules across Africa was identified.

**Agreement on recommendations**

Given the issues discussed in the preceding paragraphs around institutional fieldworkers' support structures, participants at the workshop agreed there was an urgent need to introduce more standardized and comprehensive approaches, focused on important areas that affect fieldworkers' performance and practices, including career progression, training/capacity building and management of emotional and ethical challenges. It was felt that strategies could build on the positive experiences



For peer review only - http://bmjopen.bmj.com/site/about/guidelines.xhtml

of research institutions that had already begun to institutionalize such approaches. Participants recommended a pragmatic approach, aimed at empowering and building the knowledge and skills of fieldworkers, including processes to support continuous learning through the regular reflection of the practical and ethical issues they face in everyday experience. In this way, an important element of training and support supervision was described as enhancing skills to recognise and respond to these ethical issues. The specific recommendations made are described and discussed in the following paragraphs.

i) ***Creating space for experience sharing and problem-solving***

One challenge identified during the survey was lack of psychological support after experiencing difficult and sometimes traumatic experiences. Documented evidence shows that stressful and traumatic experiences can alter the way people view themselves, the world around them, and alter how they process information and the way they behave and respond to the environment around them(Steele, 2002). A strategy described in the literature to address this challenge is the use of regular de-brief sessions, where fieldworkers are given opportunities to share experiences and consider the best approach to address challenges. During the workshop, participants recommended that field managers should create such opportunities for fieldworkers to talk about distressing or worrying issues encountered at work, through forms of feedback and reflection. This approach also requires a support supervision mechanism that is sensitive to the complex nature of the experiences fieldworkers face. This supports True Galas' (2011) arguments that having access to colleagues to discuss issues and having effective support supervision acted as a buffering factor for research front-line staff who did not engage in ethical misconduct, despite facing the same pressures to meet recruitment goals and other stressors of conducting community-based research (True, Alexander, & Richman, 2011). Combining different ways of knowing and learning will enable fieldworkers to standardize the way they handle practical and ethical issues they encounter, even where these present as dilemmas with limited information on the best course of action. At the same time, in some cases, more traditional approaches to managing emotional distress, such as staff counselling, might also be needed.

ii) ***Increasing attention on soft skills for fieldworkers***

As reported by both fieldworkers and field managers, advancement has been made in the area of technical training for research staff, including fieldworkers. Good Clinical Practice, Health and Safety, Good Clinical Laboratory Practice (GCLP) training have become a standard approach for clinical study site preparation. While these trainings enable studies to adhere to international GCP/GCLP standards, they rarely equip fieldworkers with the necessary skills to enable them to deal with practical local challenges they face on the ground. There is, therefore, a need for fieldworkers to undergo more



For peer review only - http://bmjopen.bmj.com/site/about/guidelines.xhtml

generic soft skills training, such as communication skills, basic psychology and counselling and community engagement technics to enable them to appreciate the practical local situations and develop skills to deal with the ground challenges.

iii) ***Having career development guidelines that are responsive to fieldworkers***

In a previous commentary that reported on the views of field managers who attended a deliberative workshop as part of this study, we presented important recommendations the managers made, including the need to i) increase institutional recognition of fieldworker's roles and the need for systematic and comprehensive capacity building to ensure adequate resources are allocated and that capacity building activities are well described in research proposals and grant applications; ii) develop common areas of a core curriculum for field worker capacity building to enhance quality of training processes and build their knowledge in basic biology, research approaches and methods, research ethics and research regulatory frameworks as well as data collection and documentation; respectful communication, and being aware of and managing ethical challenges and issues in practice; and iii) increase emphasis on fieldworkers' career development including developing regionally accredited training to support fieldworkers' professional development to increase individual motivation and enhance capacity for employment across different research organizations (Kombe, 2015). These recommendations should be considered in order to address the challenges presented in this paper and enhance the overall quality and integrity of research in Africa.

**CONCLUSIONS**

The important role fieldworkers play in health research can be enhanced if fieldworkers are given adequate support. The challenges they face require a combination of knowledge, skills, attitude change and ethical values that can be enhanced through structured institutional support and instilling a sense of professional responsibility and moral values to fieldworkers; through ongoing support supervision, soft skills training and creating space for deliberative discussions and experience sharing. As stated by Alexander and Richman, strengthening the capacity of fieldworkers has the potential to increase the protection of research participants, enhance research integrity and promote the collection of valid data (Alexander & Richman, 2008). Efforts to empower fieldworkers to make them better able to deal with these challenges should therefore continue. We realize that the results of this study may not be generalizable, as research institutions were selected based on specific context commonalities and association with the INDEPTH network and KWTRP. As such, more research needs to be done to explore the generalizability of these study findings. Finally, the proposed solutions may not solve all the challenges fieldworkers face, but they are certainly an important step towards improving the scientific and ethical standards of health research output from the African continent.



For peer review only - http://bmjopen.bmj.com/site/about/guidelines.xhtml

More empirical studies should be done to identify which approaches are most effective in enabling research fieldworkers to deal with the myriad challenges they face. Such research will go a long way to improving the scientific and ethical standards research, not only in Africa but internationally, given the critical global role of Africa in research.

For peer review only

## Table 1: Main characteristics of Institutions Involved in the project

| No | Name of Institution | Year Established | The main type of research | Number of Staff | Number of fieldworkers | % of FWs | Source of Funding | Description of training given to Fieldworkers | Description of career progressing system used |
|---|---|---|---|---|---|---|---|---|---|
| 1. | **Research Institution 1-WA.** | 1947 | Infectious tropical diseases | 1104 | 301 | 27.3 | MRC, UK | CORE training is given to all fieldworkers with three level, including basic, intermediary and advanced. Everybody who gets employed as a fieldworker must go through core training. | Successful completion of fieldworker straining is automatically awarded one salary grade promotion and allows fieldworkers to move through a career progression from A2 A3, B1 B2 B3, C1 C2 C3, D1 D2 D3 and E1 E2 E3. Some fieldworkers move up to C2 after the training because of the change in job description and responsibilities. |
| 2. | **Research Institution 2-SA.** | 1997 | Health and Population, HIV studies, | 800 | 52 | 5.4 | WT, NIH & EGPAF | A mass training is done for 5 weeks: 4 weeks course work and 1 week practical. The test is done to assess skills gained and certificate issued upon passing the exams. Those who pass are put on a waiting list and employed directly to studies when a vacancy arises. | A professional development process involving all staff is available. It is not specific to fieldworkers. |
| 3. | **Research Institution 3-WA.** | 1983 | Anthropology, Demography, Health Economics, Clinical trials | 153 | 40 | 26.1 | NIH and USAID, WHO & EGAD, | A centrally managed training coordinated by a social scientist who takes through the fieldworkers on how to approach the community is usually given | Fieldworkers are usually outsourced. No scheme of service is available |
| 4. | **Research Institution 4-EA.** | 1970 | Infectious diseases, prevention and control | 120 | 40 | 33.3 | NORAD, SIDA, EDCTP, WT, BMGF, WHO, TDR, GLOBVAC, Swiss TPH, | New staff are trained on the specific project and other training such as GCP, Data Management and Ethics. | There is no career ladder but if they improve their educational qualification, they get an opportunity to apply for other higher posts |
| 5. | **Research Institution 5-SA.** | 1996 | Malaria, HIV AIDS, Vaccine Trials, Pneumonia, Bacterial infection | 200 | 100 | 50.0 | AECID, MOH & EDPTP | All fieldworkers undergo project-related training, GCP, Data Management and Ethics upon being employed | None |
| 6. | **Research Institution 6-EA** | 1989 | Immunology, TB, Bacteremia, HIV, Social Science, Health Economics, | 750 | 300 | 40 | WT, IAVI, NIH | Fieldworkers training is centrally coordinated. All new fieldworkers undergo training in communication skills, research ethics, introduction to medical research, GCP and phlebotomy before being allowed to work in the field | A career progression framework for fieldworkers exists, which stipulate the entry point and career progression subject to attainment of job experience, in-house training as well as external training which could either be self or programme sponsored |
| 7. | **Research Institution 7-EA** | 1998 | Health system research, Maternal and Newborn Health & HIV | 700 | 29 | 4.2 | DFID, INDEPTH, MOH, IDRC, DFID, RF, BMGF, FP7, USAID & CDC-RBM- | Field enumerators who work in the DSS go through a refresher training every six months to share the challenges from the previous round and train them on new issues, discuss the challenges and how to address them we also remind the fieldworkers on the ethical issues for example about their own responsibilities | An interviewer and field staff can start as a field interviewer and then be a supervisor and then sometimes field manager. HR have their own arrangement but the structure is not formal |
| 8. | **Research Institution 8-EA** | 1996 | Malaria, HIV, Health and Poverty, TB | 800 | 42 | 5.3 | MOH, Swiss Institute, BMG, EU | FWs are given intensive training about how to work in the DSS and the procedures of doing the DSS activities. We also train them about ethics and their responsibilities. At the same time, other related issues concerning DSS activities are varied. | The training unit deals with all the training needs and capacity building. So a fieldworker can even be appointed to be a field supervisor or a field manager if they express interest. A structure is available |
| 9. | **Research Institution 9-EA** | 2011 | Health system research, Reproductive health | 250 | 100 | 75.6 | Wellcome Trust | New fieldworkers are paired with more experienced fieldworkers for a given period of time. They are also required to attend mandatory meetings to share their experiences. After every DSS round, a three days' refresher training is organized to recap on what they have done and solve any problems. | Training and capacity building is available for senior research staff but not for fieldworkers |
| 10. | **Research Institution 10-WA.** | 2005 | social scientist and clinical research | 90 | 50 | 50.0 | MOH & LSHTM | Training for field workers is coordinated by the leader of the study team so for every study, we have a team responsible for coordinating the fieldworkers training, who train them about field practices, community entry law and so on. | There is no formal structure for fieldworkers' career development. Only those with a degree and Masters qualification can be promoted to senior positions |
| 11. | **Research Institution 11-WA.** | 1994 | Malaria, TB, HIV, and Maternal and child health | 200 | 70 | 35.0 | LSHTM, The Kwame Nkrumah University of science and technology, University of Ghana | FWs receive training retraining on the forms they use in the field, at the end of the round and at the end of the year. Every week, they meet with their supervisors in the field to discuss any emerging issues. | Fieldworkers can be supported to undertake an advanced training in nursing or laboratory but there is no guarantee that their position would be reserved when they complete the course. A formal structure is not available |
| 12. | **Research Institution 12-EA.** | 1984 | Malaria, TB, HIV and emerging infection | 1500 | 600 | 40.0 | CDC, EDCTP and other agencies | Fieldworkers are trained on ethics, which entails Good Clinical Practice then they are paired up with more experienced ones and then trained on relevant processes and specific study document, tools and protocol training, general orientation, confidentiality issues and after this, they are left to | There are promotions on a yearly basis when a staff is being evaluated and other conditions which the staff should meet before the supervisor would recommend they get a promotion. No career progression framework for fieldworkers |
| 13. | **Research Institution 13-EA** | 1988 | Maternal and Newborn health | 35 | 12 | 34.3 | MRC UK | All fieldworkers undergo a GCP training and then trained on their specific study protocol | Career progression depends on studies. Different studies have different requirement for a career, some of them may directly be organized by the program for a certain cadre to go for training. We don't have a structure of career progression for fieldworkers |
| 14. | **Research Institution 14-SA** | 1995 | HIV and AIDS, Malaria, Non-communicable diseases | 300 | 73 | 24.3 | WT & EU | There is no standard structure. Once employed and taken through HR induction and study protocol, they are ready to go and work. There is no process to build FWs capacity to address practical and ethical challenges FWs face. | No structure available but if there is a new position, a fieldworker who has the appropriate qualification can apply. There is no formal approach to supporting fieldworkers. Sponsorship is competitive against all staff members |
| 15. | **Research Institution 15-SA** | 1979 | TB, HIV, NCD, Clinical and epidemiological research | 184 | 73 | 39.7 | WT, LSHTM, BMGF & EU | Fieldworkers get trained on demographic surveillance its basic outline and principles because most studies are linked to our Continuous Registration System (CRS). They are also trained on HIV counselling and testing and data entry. | No formal career path exists |
| 16. | **Research Institution 16-WA** | 1992 | Vaccine and Drug trials, fundamental sciences, Mosquitoes genetics, Parasites genetics, Drug Resistance (PCR, in Vitro tests…), Malaria Immunology, Malaria and Pregnancy trial | 45 | 30 | 66.7 | NIH, MOH and EDCTP | Fieldworkers are given protocol-specific training and told about their responsibilities and objectives of the study. | Training/Career Development Program is in place to increase the number of qualified scientific and technical personnel capable of conducting independent medical research. This includes post-doctoral training-PhD levels, Parasitology, Cellular Biology, Pharmacology, Immunology; Maters of Sciences: Clinical research, Clinical Pharmacology, Public Health, Parasitology, Entomology, Nutrition. No specific framework for fieldworkers |
| 17. | **Research Institution 17-WA.** | 1998 | Maternal and child health, tropical diseases; malaria, Reproductive Health, Communicable diseases; Non-communicable diseases; human genetics; Health systems research, Social and Behavioural Studies, Poverty and Equity Studies | 263 | 193 | 73 | Ghana Health Service, Rockefeller Foundation, Population Council- USA, USAID | Fieldworkers training depends on the nature of the project, so for instance, if you are doing a survey on the health system, you will be taken through that and the overview of the overall Centre, community engagement and ethics. The project staff and the PI undertake the training | If a junior/frontline staff goes for further studies and has worked for 10 or more years and the field of study is relevant to their line of work, then the Centre, they may be supported. There is no formal structure to support fieldworkers. |
| 18. | **Research Institution 18-WA.** | 2007 | Molecular biology, immunology, clinical biology and bioinformatics | Staff are contracted from other organizations | FWs are contracted through other organization on a need basis then laid off at the end of a study | Depends on the project | National government, UNI-AID, Liverpool University, NIH and Fogarty foundation | New fieldworkers are usually given a Standard Operational Procedure then shown what they are going to do after that, they can be allowed to work in the field. | FWs are contracted through other organization on a need basis then laid off at the end of a study |
| 19. | ***Note: A discussion with MRC-UK and the INDEPTH secretariat in Ghana was also held with a view to crosscheck the information gathered from the interviews held** | | | | | | | | |
| **Total- 7 Research Institution from West Africa, 4 Research Institution from Southern Africa and 7 Research Institution from East Africa and 1 regional research coordination institution from Africa and 1 UK research funding institution** | | | | | | | | | |

For peer review only - http://bmjopen.bmj.com/site/about/guidelines.xhtml

**References**

Alexander, L. B., & Richman, K. A. (2008). Ethical Dilemmas in Evaluations Using Indigenous Research Workers. *American Journal of Evaluation, 29*(1), 73-85.

Angwenyi, V., Kamuya, D., Mwachiro, D., Marsh, V., Njuguna, P., & Molyneux, S. (2013). Working with Community Health Workers as 'volunteers' in a vaccine trial: practical and ethical experiences and implications. *Dev World Bioeth, 13*(1), 38-47. doi:10.1111/dewb.12015

Chantler, T., Otewa, F., Onyango, P., Okoth, B., Odhiambo, F., Parker, M., & Geissler, P. W. (2013). Ethical challenges that arise at the community interface of health research: village reporters' experiences in Western Kenya. *Dev World Bioeth, 13*(1), 30-37. doi:10.1111/dewb.12023

Glickman, S. W., McHutchison, J. G., Peterson, E. D., Cairns, C. B., Harrington, R. A., Califf, R. M., & Schulman, K. A. (2009). Ethical and scientific implications of the globalization of clinical research. *New England Journal of Medicine, 360*(8), 816-823.

Kamuya, D. M., Theobald, S. J., Munywoki, P. K., Koech, D., Geissler, W. P., & Molyneux, S. C. (2013). Evolving friendships and shifting ethical dilemmas: fieldworkers' experiences in a short term community based study in Kenya. *Dev World Bioeth, 13*(1), 1-9. doi:10.1111/dewb.12009

Kingori. (2013). Experiencing everyday ethics in context: frontline data collectors perspectives and practices of bioethics. *Soc Sci Med, 98*, 361-370. doi:S0277-9536(13)00561-3 [pii]
10.1016/j.socscimed.2013.10.013

Kombe, F. (2015). Enhancing quality and integrity in biomedical research in Africa: an international call for greater focus, investment and standardisation in capacity strengthening for frontline staff. *BMC medical ethics, 16*(1), 77.

Lansang, M. A., & Dennis, R. (2004). Building capacity in health research in the developing world. *Bulletin of the World Health Organization, 82*(10), 764-770.

Molyneux, S., Kamuya, D., Madiega, P. A., Chantler, T., Angwenyi, V., & Geissler, P. W. (2013). Field workers at the interface. *Dev World Bioeth, 13*(1), ii-iv. doi:10.1111/dewb.12027

Participants of an International Workshop in Kenya on the Role of Frontline Staff in Biomedical Research, J., & Kombe, F. (2015). Enhancing quality and integrity in biomedical research in Africa: an international call for greater focus, investment and standardisation in capacity strengthening for frontline staff. *BMC Med Ethics, 16*(1), 77. doi:10.1186/s12910-015-0071-3

Steele, W. (2002). Trauma's Impact on Learning. *Trauma and Loss: Research and Interventions*.

True, G., Alexander, L. B., & Richman, K. A. (2011). Misbehaviors of front-line research personnel and the integrity of community-based research. *J Empir Res Hum Res Ethics, 6*(2), 3-12. doi:10.1525/jer.2011.6.2.3

Website, I. N.

Chantler, T., Otewa, F., Onyango, P., Okoth, B., Odhiambo, F., Parker, M., & Geissler, P. W. (2013). Ethical Challenges that Arise at the Community Interface of Health R esearch: Village R eporters' Experiences in Western K enya. *Developing World Bioethics*, *13*(1), 30–37.

Fottrell, E., & Byass, P. (2010). Verbal autopsy: methods in transition. *Epidemiologic Reviews*, *32*(1), 38–55.

Kamuya, D. M., Theobald, S. J., Marsh, V., Parker, M., Geissler, W. P., & Molyneux, S. C. (2015). The one who chases you away does not tell you go: Silent refusals and complex power relations in research consent processes in coastal Kenya. *PLoS ONE*, *10*(5). https://doi.org/10.1371/journal.pone.0126671

Kamuya, D. M., Theobald, S. J., Munywoki, P. K., Koech, D., Geissler, W. P., & Molyneux, S. C. (2013). Evolving Friendships and Shifting Ethical Dilemmas: Fieldworkers' Experiences in a Short Term Community Based Study in K enya. *Developing World Bioethics*, *13*(1), 1–9.

Kingori, P. (2013). Experiencing everyday ethics in context: Frontline data collectors perspectives and practices of bioethics. *Social Science & Medicine*, *98*, 361–370.

Kombe, F., Anunobi, E. N., Tshifugula, N. P., Wassenaar, D., Njadingwe, D., Mwalukore, S., … Dlamini, P. S. (2014). Promoting Research Integrity in A frica: An African Voice of Concern on Research



For peer review only - http://bmjopen.bmj.com/site/about/guidelines.xhtml

Misconduct and the Way Forward. *Developing World Bioethics*, *14*(3), 158–166.

Madiega, P. A., Jones, G., Prince, R. J., & Geissler, P. W. (2013). 'She's My Sister-In-Law, My Visitor, My Friend'–Challenges of Staff Identity in Home Follow-Up in an HIV Trial in W estern K enya. *Developing World Bioethics*, *13*(1), 21–29.

Marshall, P. A., & Marshall, P. L. (2007). *Ethical challenges in study design and informed consent for health research in resource-poor settings* (Vol. 5). World Health Organization.

Molyneux, S., Kamuya, D., Madiega, P. A., Chantler, T., Angwenyi, V., & Geissler, P. W. (2013). Field Workers at the Interface. *Developing World Bioethics*, *13*(1). https://doi.org/10.1111/dewb.12027

Simon, C., & Mosavel, M. (2010). Response to open peer commentaries on "Community members as recruiters of human subjects: ethical considerations." *The American Journal of Bioethics*, *10*(3), W1–W3.

Srivastava, A., & Thomson, S. B. (2009). Framework analysis: a qualitative methodology for applied policy research.

Wax, M. L. (1977). On fieldworkers and those exposed to fieldwork: Federal regulations and moral issues. *Human Organization*, *36*(3), 321–328.

Zahr, C. A., & Pinchuk, M. (2007). *Verbal autopsy standards: ascertaining and attributing cause of death*. World Health Organization.

Zinn, M. B. (1979). Field research in minority communities: Ethical, methodological and political observations by an insider. *Social Problems*, *27*(2), 209–219.

For peer review only



For peer review only - http://bmjopen.bmj.com/site/about/guidelines.xhtml

**Contributor and guarantor information**

All authors were involved in conceptualizing the idea and contributed in writing the manuscript. Francis Kombe contributed to the planning, conduct, and reporting of the work described in the article and is responsible for the overall content. Vicki Marsh and Dorothy Ikamba assisted with reviewing the transcripts and the development of themes and sub-themes during the analysis. Sassy Molyneux, Dorcas Kamuya and Samson Kinyanju contributed in designing the initial research ideas, reviewed the data collected and were involved in drafting the initial manuscript and edited subsequent versions. All authors reviewed and approved the final manuscript for publication. Francis Kombe accepts full responsibility for the work and the conduct of the study. He had full access to the data, and controlled the decision to publish. Francis Kombe attests that all listed authors meet authorship criteria and that no others meeting the criteria have been omitted.

**Copyright/license for publication**

Francis Kombe has the right to grant on behalf of all authors and does grant on behalf of all authors, a worldwide license to the Publishers and its licensees in perpetuity, in all forms, formats and media (whether known now or created in the future), to i) publish, reproduce, distribute, display and store the Contribution, ii) translate the Contribution into other languages, create adaptations, reprints, include within collections and create summaries, extracts and/or, abstracts of the Contribution, iii) create any other derivative work(s) based on the Contribution, iv) to exploit all subsidiary rights in the Contribution, v) the inclusion of electronic links from the Contribution to third party material where-ever it may be located; and, vi) license any third party to do any or all of the above

**Data sharing statement**

No additional data available

**Transparency statement**

Francis Kombe affirms that the manuscript is an honest, accurate, and transparent account of the study being reported; that no important aspects of the study have been omitted; and that any discrepancies from the study as originally planned have been explained.

**Competing interests**

The views on this paper are the authors own viewpoints and do not represent the position of their organizations. The authors declare no conflict of interests.
The authors declared that they have no competing interests.

**Ethical approval**

The paper is published with the permission of the Centre director, KEMRI. All the participant who participate in the telephone survey as well as those who attended the consultative workshop gave individual consent to participate in the study and the workshop, and permitted the publication of this manuscript. Permission to conduct this study was granted by a review conducted by the Centre for Geographic Medicine Research, Coast (CGMRC)- Centre



For peer review only - http://bmjopen.bmj.com/site/about/guidelines.xhtml

Specific Committee and a final Ethical approval given by the Research and Scientific Review Unit (SERU) at the Kenya Medical Research Institute (KEMRI).

For peer review only



For peer review only - http://bmjopen.bmj.com/site/about/guidelines.xhtml

**DISCUSSION GUIDE FOR RESEARCH INSTITUTIONS WORKING WITH FIELDWORKERS (FWs) IN SSA**

**TITLE: STRENGTHENING TRAINING OF FRONT LINE (FWs) STAFF AS A MEANS TO ENHANCE COMMUNITY ENGAGEMENT**

## SECTION A: Institutional Background

1. Name of organization ____________________________________________
2. Vision ____________________________________________
3. Mission ____________________________________________
4. Motto ____________________________________________
5. Year organization was established [__][__][__] [__]
6. Source of main funding for the organization ____________________________________
7. Other funding agencies and collaborators ____________________________________
8. Types of research conducted at the organization: ____________________________________

## SECTION B: Frontline staff Managers' Perspective[1]

*This section explores managers' perceptions of the roles of and practical and ethical challenges faced by interface staff when discharging their duties and the institutional support supervision, performance management and Continuing Professional Development (CPD) practices*

1. What names are given to your frontline staff whose primary role involves recruiting and consenting research participants into studies?

2. What key roles/activities do they undertake in your organization?

3. In your institution, how many staff does it have (in total) and how many of these are employed as frontline staff?

4. What is the range of qualifications for frontline staff and what is their minimum level of education/qualification?

5. What is the nature of their employment e.g. are they in contract? What is the average contract period? Are the contracts renewable and what is considered during renewal? Any systems that ensure retention of frontline staff in the institutions?

6. What systems are in place to coordinate the way frontline staff are employed and retained across different studies in the institutions? What is the role of PI (if any) in retaining frontline staff?

7. What formal and informal processes are in place to induct new frontline staff when they first arrive, and to train them for their roles?

[1] Manager is the person whose primary responsibility involves working directly with front line staff

**DISCUSSION GUIDE FOR RESEARCH INSTITUTIONS WORKING WITH FIELDWORKERS (FWs) IN SSA**

**TITLE: STRENGTHENING TRAINING OF FRONT LINE (FWs) STAFF AS A MEANS TO ENHANCE COMMUNITY ENGAGEMENT**

---

*8.* How is the training for interface staff organized? Are there any refresher trainings/skills improvement activities? What are the *training approach eg frequency and objectives of the training,*

*9.* How is the frontline staff training managed? Describe funding, design, whether centralized or decentralized, and monitoring and evaluation of the training

*10.* Describe any practices, experiences and studies (if any) that have informed the current training eg feedback mechanisms; availability of a community engagement strategy, community Knowledge Attitude and Perception (KAP) surveys etc

*11.* How do you ensure skills learned are assimilated into the frontline's day to day activities? Are there any support supervision mechanisms, performance appraisals etc ? How are these implemented?

*12.* Describe any career progression scheme used by your organization for these staff groups

*13.* What challenges do you experience in working with and managing frontline staff?

## SECTION C: Frontline staffs' Experience-Staff perspective

*This section explores frontline staff's practical and ethical challenges faced as they discharge their duties and their perceptions of the institutional support in training and CPD*

1. What are roles and responsibilities of frontline staff?

2. What challenges do frontline staff face when interacting with community members? *Practical and ethical challenges/dilemmas faced, e.g. issues of confidentiality?*

3. What type of in-house and external training is given to frontline staff in your institution?
   - ***Which of these do you perceive to be most appreciated and for what reason?***

4. What are the perceived institutional challenges in supporting and capacity building of frontline staff?
   - ***How do frontline members of staff address the above challenges?***

## SECTION D: Planned Workshop

At the end of this survey, we hope to invite a selected number of the institutions to a 2 day workshop. Kindly indicate if you would like to be considered to attend this workshop.

For peer review only

**STRENGTHENING TRAINING OF FRONT LINE STAFF AS A MEANS TO ENHANCE COMMUNITY ENGAGEMENT**

**INFORMATION SHEET FOR MANAGERS AND SENIOR FRONTLINE STAFF**

## What is this survey all about?

This survey will be conducted through telephone/ teleconference discussion. One front line staff manager (people who have a direct role of providing support supervision to frontline staff) and one senior frontline staff member from a total of 20 health research institutions in Sub-Saharan Africa will be invited to participate in the discussion.

The aim of the discussion is to identify practical and ethical challenges experienced by frontline staff in their day to day activities and the available support supervision, performance management and Continuing Professional Development (CPD) practices within these institutions. Later, representatives from a few of the 20 institutions will be invited to a consultative workshop to follow up and discuss these issues in detail.

The report generated from the survey and workshop will be shared with all institutes for their own use. In the long term, the report could be used to inform further discussions on the potential of developing a generic curriculum for frontline staff training.

## What is expected of you if you take part in this discussion?

If you agree to take part in this discussion, we will request you to select a date and time when you would prefer the moderator to call and have the discussion with you. We will then call and ask a few questions with regards to your perceptions of the roles of frontline staff and the institutional support supervision, monitoring and evaluation and general CPD practices. These questions are shown on the attached discussion guide for your attention. The discussion is expected to take a maximum of 30 minutes.

Participation in this interview is voluntary. While it is important to answer all the questions, feel free to skip any question that you do not feel comfortable to answer and the moderator will continue asking the next question. You can also decide to terminate the discussion at any time if you decide not to continue with the discussion for any reason.

## Is there any individual benefit for taking part in this discussion?

There is no individual benefit in taking part in this discussion. However, by sharing your experiences in working with frontline staff, you will assist in contributing to knowledge that could improve the way frontline staff deal with their practical and ethical challenges as well as their support supervision, performance and general CPD management practices in future.

## If you have any question

If you have any question about this survey, please contact any of the following people through the email addresses provided below


| | | |
|---|---|---|
| Francis Kombe<br>KWTRP<br>P.O BOX 230, KILIFI<br>Fkombe@kemri-wellcome.org | Sam Kinyanjui<br>KWTRP<br>P.O BOX 230, KILIFI<br>Skinyajui@kemri-wellcome.org | Dorcas Kamuya<br>KWTRP/ Ethox-Nuffield<br>Department of Population<br>Science, Oxford University<br>P.O BOX 230, KILIFI<br>Dkamuya@kemri-wellcome.org |


For peer review only

BMJ Open

# Enhancing Fieldworkers' Performance Management Support in Health Research: Views of Field managers and Fieldworkers from major research centres in Africa



| | |
|---|---|
| Journal: | *BMJ Open* |
| Manuscript ID | bmjopen-2018-028453.R1 |
| Article Type: | Original research |
| Date Submitted by the Author: | 18-May-2019 |



| | |
|---|---|
| Complete List of Authors: | Kombe, Francis; Centre for Geographic Medicine Research Coast; African Research Integrity Network<br>Marsh, V; Centre for Geographic Medicine Research Coast; Centre for Tropical Medicine and Global Health, Nuffield Department of Medicine, Oxford University, UK<br>Molyneux, Sassy ; Centre for Geographic Medicine Research Coast; Centre for Tropical Medicine and Global Health, Nuffield Department of Medicine, Oxford University, UK<br>Kamuya, Dorcas; KEMRI Wellcome Trust Research Programme; Ethox Centre, Nuffield Department of Population Health, Oxford University, UK<br>Ikamba, Dorothy; KEMRI Wellcome Trust Research Programme<br>Kinyanjui, Samson; Centre for Geographic Medicine Research Coast; Centre for Tropical Medicine and Global Health, Nuffield Department of Medicine, Oxford University, UK |



| | |
|---|---|
| <b>Primary Subject Heading</b>: | Ethics |
| Secondary Subject Heading: | Ethics, Qualitative research |
| Keywords: | Fieldworkers, Africa, Capacity Building, Health Research, Research Ethics, Research Integrity |
| | |


SCHOLARONE™
Manuscripts

**Enhancing Fieldworkers' Performance Management Support in Health Research: *Views of Field managers and Fieldworkers from major research centres in Africa***

Francis Kombe[1], Vicki Marsh[1,3], Sassy Molyneux[1,3], Dorcas Kamuya[1,2], Dorothy Ikamba[1], Kinyanjui, Samson Muchina[1,3]

[1]KEMRI Wellcome Trust Research Programme, Kenya; [2]Ethox Centre, Nuffield Department of Population Health, Oxford University, UK; [3]Centre for Tropical Medicine and Global Health, Nuffield Department of Medicine, Oxford University, UK.

**Corresponding author**: Francis Kombe, KEMRI Wellcome Trust Research Programme, PO Box 230, Kilifi 80108, Kenya. Mobile +254 722459045; Email-Kajoleh@gmail.com

**Acknowledgements**: We acknowledge and greatly appreciate the support of all research institutions' directors who allowed their field managers and field workers to take part in this project, and all the field managers and field workers who participated. We are extremely indebted to Prof Mike Parker, the Director of the Wellcome Centre for Ethics and Humanities and Director of Ethox Centre at the University of Oxford for his support and guidance during the project; and all the senior field managers and delegates who attended the consultative workshop. The manuscript is published with the support of the Director, KEMRI.

**Funding**:

This project was funded by Global Health Bioethics Network-UK (Wellcome Trust grant no. OXF-GHB02) and supplemented by KWTRP Capacity Building Strategic Award



For peer review only - http://bmjopen.bmj.com/site/about/guidelines.xhtml

(Wellcome Trust grant no 084538). The research and capacity building activities of the Global Health Bioethics Network are supported by a Wellcome Trust Strategic Award (096527)

**Keywords:** Africa, Fieldworkers, Research Integrity, Research Ethics, Capacity Building.

**Word Count: 6417**

For peer review only



For peer review only - http://bmjopen.bmj.com/site/about/guidelines.xhtml

**ABSTRACT**

**Introduction**

Fieldworkers are part of the system that promotes scientific and ethical standards in research, through data collection, seeking consent and providing support in research, due to their insider cultural knowledge and fluency in local languages. The credibility and integrity of community-based research therefore rely heavily on how this cadre of staff adheres to institutional and research procedures and guidelines.

**Study objectives**

This study mapped out existing practices in training, support and performance management of fieldworkers in Africa, described fieldworker and their managers' experiences, and lessons learned. Consultative process, involving field managers from 15 international health research institutions, were used to identify appropriate ways of addressing the challenges faced by fieldworkers working in Africa.

**Methods**

**Phase one-** We conducted 32 telephone interviews with 20 field managers and 12 senior fieldworkers from 18 major research Centres in Africa, MRC-UK and the INDEPTH Network Secretariat.

**Phase two-** We held a two-day workshop involving 25 delegates including 18 from the institutions that were involved in phase I; and 7 additional stakeholders from the KWTRP. Data transcribed from the interviews and workshop proceedings were analyzed thematically using Nvivo 10 software.

**Results**

Most institutions employed fieldworkers, usually with 12 years of formal education and residing within the geographic areas of research, to support studies. Although their roles were common, there were marked differences in the type of training, professional development schemes and fieldworkers support. Fieldworkers faced various challenges, with the potential to affect their ethical and scientific practices.

**Conclusion**

Fieldworkers undertake vital tasks that promote the quality of data and ethical practice in research studies. There is, therefore, a need for research institutions to enhance structured support, supervision, soft skills training for fieldworkers and to provide space for discussion, reflection and experience-sharing to help fieldworkers tackle the practical and ethical challenges they face.




For peer review only - http://bmjopen.bmj.com/site/about/guidelines.xhtml

**Strengths and limitations of this study**

- Findings of this study add to a growing body of literature on how to enhance the quality and integrity of data collected with the support of fieldworkers; an important, but largely under supported and understudied cadre of staff, in international research settings in Africa.
- The fact that participants of this study were drawn from West, South and East Africa makes the findings of this study rich with an extended spectrum of experiences adduced, which provides deeper insights to the issues that were discussed.
- The use of deliberative approaches, that allowed issues raised during telephone interviews to be followed up and discussed further in a workshop, provided a unique opportunity to cross-check data from fieldworkers with their managers, reflect on and unpack important issues, with a view to identifying relevant and applicable strategies for addressing challenges faced.
- All interviews were done by phone. As such, important interview dynamics may have been missed. We do however believe that this did not affect the quality of data collected.
- Secondly, interviewees were nominated by Centre directors, who were the primary contact people. Although individual consent was obtained from those nominated by the directors prior to the interview, it is possible that interviewees could have reported what they believed would be acceptable to their directors. During the interviews, we assured our participants about the confidentiality of the data they would share, which we believe enabled them to share information sincerely and transparently.
- Thirdly, because our interviews focused on fieldworkers challenges and potential opportunities, this could have raised expectations from the fieldworkers who were interviewed hence leading to overreporting of the challenges they experienced. The fact that fieldworkers' managers reported similar challenges supports the validity of the fieldworkers data.
- The interviewer was a senior community facilitator at KWTRP and had worked as a fieldworker prior to being promoted to this position. It is possible that his personal experience influenced his interpretation of the data. Continuous discussions were held with VM and DK to discuss emerging themes from the data and reflect on the his positionality in the data collection process. This enabled him to reflect on his potential influence, in the data collection process
- Finally, institutions that participated in this study were selected on the basis of existing relationship with the INDEPTH Network and the KWTRP. It is possible that other institutions outside these networks had different experiences hence these data may not be generalizable to all health research institutions in Africa. However, we believe the institutions involved had a wide range of characteristics hence results from this study can inform practice in many institutions within and without Africa, that share similar characteristics as those involved in this study and that employ fieldworkers to support in their research.



For peer review only - http://bmjopen.bmj.com/site/about/guidelines.xhtml

For peer review only

## 1. BACKGROUND

High-quality data is a critical output of all science worldwide. Thus, research institutions put significant emphasis on generating data that are of the highest scientific and ethical standards. In many research programmes in low and middle-income countries, much of the data is collected by frontline staff referred to as “fieldworkers”. This cadre of staff is also often responsible for recruiting potential research participants, administering consent forms and following up participants during studies (1). As such, beyond ensuring data integrity, fieldworkers play a key role in ensuring the ethical implementation of research protocols. The role of fieldworkers is particularly critical in international collaborative programmes with researchers drawn from outside local communities. In such settings, fieldworkers, who are normally from the local communities, can serve as the face of research programmes with potential to significantly influence communities’ trust in research.

In carrying out these complex roles, fieldworkers face practical and ethical dilemmas that require the balancing of institutional roles and guidelines against community expectations and fieldworkers’ personal interests (2). Hence, fieldworkers continuously make independent (often unconscious) decisions on how to apply ‘ethical principles’ on the ground. For example fieldworkers reported challenges such as dealing with ‘silent refusals’ (3) (where participants avoid key study procedures but do not openly refuse), and with many requests for health and other social- economic support that are not included in the study protocol (4-8).

### 1.1 Capacity Building and Investiment In Research

Over the years there has been an increasing interest and investment in developing research capacity in Africa and other low and middle income regions through long-term initiatives such as the WHO-Tropical Diseases Research Programme (TDR) (9), the European and Developing Countries Clinical Trials Initiative Partnership (EDCTP) and more recently, The Wellcome Trust-DFID funded Developing Excellence in Leadership, Training and Science scheme (DELTAS) Africa. Efforts to strengthen research capacity are likely to continue, as the trend towards greater cross-border data sharing, globalization of clinical research and comparability of data quality at the global level continues (10).

Given that fieldworkers play such a vital role in health research, it would be expected that research programmes, in addition to training scientists, would also focus on building fieldworkers’ capacity to enable them to carry out their duties in the most effective manner. However, fieldworkers’ capacity and skill sets have received relatively little attention. The mechanisms and processes by which research institutions in Africa support fieldworkers in their execution of duties remain unclear. Apart from the Good Clinical Practice guidelines, which focus primarily on clinical trials, no other guidelines



For peer review only - http://bmjopen.bmj.com/site/about/guidelines.xhtml

are available that provide a framework for the content and approach for supporting the Continuing Professional Development and career growth of fieldworkers.

Understanding the challenges that fieldworkers face and the type of systems, and level of support research institutions in Africa provide, can enable us to identify opportunities for strengthening fieldworkers' scientific and ethical practices.

In this paper, we draw on our findings from a two-phase qualitative study on the perceptions, experiences and views of field managers and fieldworkers on the structural and performance management support extended to health research fieldworkers with the aim of informing institutional policies that promote research integrity and ethical practices for this critical cadre of staff (11).

## 2. STUDY OBJECTIVES

This study mapped out existing practices in relation to training, support and performance management of fieldworkers in sub-Saharan Africa, fieldworkers' and their managers' experiences, and lessons learned. In addition, field managers from international health research institutions were involved in a consultative workshop to identify appropriate ways of addressing the challenges faced by fieldworkers working in research Centres in Africa.

## 3. METHODS

The study was planned over two phases, focused on understanding structural and performance management practices and their implications within institutions. In Phase one, which was done in February 2014, we conducted semi-structured telephone interviews with 32 fieldworker managers and experienced fieldworkers across 18 health research institutions in 14 countries in Africa and the United Kingdom. In Phase two, which was done in July 2014, we held a two-day workshop involving 25 delegates including 18 (one per institution) from the institutions that were involved in phase I; and the director of training, three senior social scientists with long term experience in managing fieldworkers (also Co-PIs of the project), the Head of community engagement and two senior fieldworkers' supervisor from the KWTRP- Total 25 participants (12).

Although this paper aims to focuses more on the results from Phase I, it is important to note that the same participants who attended the workshop were also interviewed during the telephone interviews (Phase I). There may therefore be some overlaps in certain sections of these papers. It is also important to note that in Phase II (the workshop} issues raised in phase 1 were re-discussed in more detail with a view to build consensus and identify common recommendations and strategies for addressing the reported challenges. As such, there are certain sections that inevitably refer to views



For peer review only - http://bmjopen.bmj.com/site/about/guidelines.xhtml

shared during the workshop. It is however, not the intention of the authors to publish the phase II results of this study, (12) that have already been published.

### 3.1 Patient and public involvement

No patient involved

### 3.2 Study site and selection of research institutions and interview participants.

This study was led by a team based at the Kenya Medical Research Institute (KEMRI) Wellcome Trust Research Programme (KWTRP) in Kenya. The KWTRP is a long-standing international health research institution with a wide range of existing scientific and capacity building collaborations with other research institutes in sub-Saharan Africa (see http://kemri-wellcome.org/). During the first phase, our sampling strategy for research institutions, and relevant individuals within these, focused on African institutions that manage Health and Demographic Surveillance Systems, given their employment of significant numbers of fieldworkers. We identified these institutions from our existing scientific and capacity building linkages, including the INDEPTH Network (http://www.indepth-network.org), an international research network for health and demographic surveillance sites in Africa. The decision to invite institutions affiliated to INDEPTH Network was also informed by the fact that the INDEPTH Network encompasses all research centers in sub-Saharan Africa that have Health Demographic Surveillance Systems which in themselves require a significant amount of fieldwork and are also used as platforms for other field studies.

From the original list of 34 institutions obtained from the KWTP training department and INDEPTH site, we sent an email to the directors with an information sheet about the study to invite them to participate in the study. A total of eighteen African international research institutions (see table 1) and the MRC-UK and the INDEPTH Network Secretariat in Ghana responded positively. 16 directors did not reply to our email and could not therefore determine why they did not respond. These were dropped after three reminders. The eighteen African research institutions included a mix of institutions with varied years of operations, employed a diverse number of fieldworkers, engaged in a range of local and international collaborations and specialized in different research programmes/thematic areas. This mixture of characteristics was important in maximizing shared learning and experiences across institutions. In each institution, we wrote to the director and sought permission to interview one senior fieldworker and one field manager. In the letter, we particularly underscored the need to select a manager whose primary responsibility involved working directly with and managing fieldworkers, including offering support supervision, training and other forms of performance management.



For peer review only - http://bmjopen.bmj.com/site/about/guidelines.xhtml

## Table 1: Main characteristics of Institutions Involved in the project

| No | Name of Institution | Year Established | The main type of research | Number of Staff | Number of fieldworkers | % of FWs | Source of Funding | Description of training given to Fieldworkers | Description of career progressing system used |
|---|---|---|---|---|---|---|---|---|---|
| 1. | **Research Institution 1-WA.** | 1947 | Infectious tropical diseases | 1104 | 301 | 27.3 | MRC, UK | CORE training is given to all fieldworkers with three level, including basic, intermediary and advanced. Everybody who gets employed as a fieldworker must go through core training. | Successful completion of fieldworker straining is automatically awarded one salary grade promotion and allows fieldworkers to move through a career progression from A2 A3, B1 B2 B3, C1 C2 C3, D1 D2 D3 and E1 E2 E3. Some fieldworkers move up to C2 after the training because of the change in job description and responsibilities. |
| 2. | **Research Institution 2-SA.** | 1997 | Health and Population, HIV studies, | 800 | 52 | 5.4 | WT, NIH & EGPAF | A mass training is done for 5 weeks: 4 weeks course work and 1 week practical. The test is done to assess skills gained and certificate issued upon passing the exams. Those who pass are put on a waiting list and employed directly to studies when a vacancy arises. | A professional development process involving all staff is available. It is not specific to fieldworkers. |
| 3. | **Research Institution 3-WA.** | 1983 | Anthropology, Demography, Health Economics, Clinical trials | 153 | 40 | 26.1 | NIH and USAID, WHO & EGAD, | A centrally managed training coordinated by a social scientist who takes through the fieldworkers on how to approach the community is usually given | Fieldworkers are usually outsourced. No scheme of service is available |
| 4. | **Research Institution 4-EA.** | 1970 | Infectious diseases, prevention and control | 120 | 40 | 33.3 | NORAD, SIDA, EDCTP, WT, BMGF, WHO, TDR, GLOBVAC, Swiss TPH, | New staff are trained on the specific project and other training such as GCP, Data Management and Ethics. | There is no career ladder but if they improve their educational qualification, they get an opportunity to apply for other higher posts |
| 5. | **Research Institution 5-SA.** | 1996 | Malaria, HIV AIDS, Vaccine Trials, Pneumonia, Bacterial infection | 200 | 100 | 50.0 | AECID, MOH & EDPTP | All fieldworkers undergo project-related training, GCP, Data Management and Ethics upon being employed | None |
| 6. | **Research Institution 6-EA** | 1989 | Immunology, TB, Bacteremia, HIV, Social Science, Health Economics, | 750 | 300 | 40 | WT, IAVI, NIH | Fieldworkers training is centrally coordinated. All new fieldworkers undergo training in communication skills, research ethics, introduction to medical research, GCP and phlebotomy before being allowed to work in the field | A career progression framework for fieldworkers exists, which stipulate the entry point and career progression subject to attainment of job experience, in-house training as well as external training which could either be self or programme sponsored |
| 7. | **Research Institution 7-EA** | 1998 | Health system research, Maternal and Newborn Health & HIV | 700 | 135 | 19.3 | DFID, INDEPTH, MOH, IDRC, DFID, RF, BMGF, FP7, USAID & CDC-RBM- | Field enumerators who work in the DSS go through a refresher training every six months to share the challenges from the previous round and train them on new issues, discuss the challenges and how to address them we also remind the fieldworkers on the ethical issues for example about their own responsibilities | An interviewer and field staff can start as a field interviewer and then be a supervisor and then sometimes field manager. HR have their own arrangement but the structure is not formal |
| 8. | **Research Institution 8-EA** | 1996 | Malaria, HIV, Health and Poverty, TB | 800 | 183 | 22.9 | MOH, Swiss Institute, BMG, EU | FWs are given intensive training about how to work in the DSS and the procedures of doing the DSS activities. We also train them about ethics and their responsibilities. At the same time, other related issues concerning DSS activities are varied. | The training unit deals with all the training needs and capacity building. So a fieldworker can even be appointed to be a field supervisor or a field manager if they express interest. A structure is available |
| 9. | **Research Institution 9-EA** | 2011 | Health system research, Reproductive health | 250 | 100 | 75.6 | Wellcome Trust | New fieldworkers are paired with more experienced fieldworkers for a given period of time. They are also required to attend mandatory meetings to share their experiences. After every DSS round, a three days' refresher training is organized to recap on what they have done and solve any problems. | Training and capacity building is available for senior research staff but not for fieldworkers |
| 10. | **Research Institution 10-WA.** | 2005 | social scientist and clinical research | 90 | 50 | 50.0 | MOH & LSHTM | Training for field workers is coordinated by the leader of the study team so for every study, we have a team responsible for coordinating the fieldworkers training, who train them about field practices, community entry law and so on. | There is no formal structure for fieldworkers' career development. Only those with a degree and Masters qualification can be promoted to senior positions |
| 11. | **Research Institution 11-WA.** | 1994 | Malaria, TB, HIV, and Maternal and child health | 200 | 100 | 50.0 | LSHTM, The Kwame Nkrumah University of science and technology, University of Ghana | FWs receive training retraining on the forms they use in the field, at the end of the round and at the end of the year. Every week, they meet with their supervisors in the field to discuss any emerging issues. | Fieldworkers can be supported to undertake an advanced training in nursing or laboratory but there is no guarantee that their position would be reserved when they complete the course. A formal structure is not available |
| 12. | **Research Institution 12-EA.** | 1984 | Malaria, TB, HIV and emerging infection | 1500 | 630 | 42.0 | CDC, EDCTP and other agencies | Fieldworkers are trained on ethics, which entails Good Clinical Practice then they are paired up with more experienced ones and then trained on relevant processes and specific study document, tools and protocol training, general orientation, confidentiality issues and after this, they are left to | There are promotions on a yearly basis when a staff is being evaluated and other conditions which the staff should meet before the supervisor would recommend they get a promotion. No career progression framework for fieldworkers |
| 13. | **Research Institution 13-EA** | 1988 | Maternal and Newborn health | 380 | 105 | 27.6 | MRC UK | All fieldworkers undergo a GCP training and then trained on their specific study protocol | Career progression depends on studies. Different studies have different requirement for a career, some of them may directly be organized by the program for a certain cadre to go for training. We don't have a structure of career progression for fieldworkers |



For peer review only - http://bmjopen.bmj.com/site/about/guidelines.xhtml

| | | | | | | | | | |
|---|---|---|---|---|---|---|---|---|---|
| 14. | **Research Institution 14-SA** | 1995 | HIV and AIDS, Malaria, Non-communicable diseases | 300 | 73 | 24.3 | WT & EU | There is no standard structure. Once employed and taken through HR induction and study protocol, they are ready to go and work. There is no process to build FWs capacity to address practical and ethical challenges FWs face. | No structure available but if there is a new position, a fieldworker who has the appropriate qualification can apply. There is no formal approach to supporting fieldworkers. Sponsorship is competitive against all staff members |
| 15. | **Research Institution 15-SA** | 1979 | TB, HIV, NCD, Clinical and epidemiological research | 184 | 73 | 39.7 | WT, LSHTM, BMGF & EU | Fieldworkers get trained on demographic surveillance its basic outline and principles because most studies are linked to our Continuous Registration System (CRS). They are also trained on HIV counselling and testing and data entry. | No formal career path exists |
| 16. | **Research Institution 16-WA** | 1992 | Vaccine and Drug trials, fundamental sciences, Mosquitoes genetics, Parasites genetics, Drug Resistance (PCR, in Vitro tests…), Malaria Immunology, Malaria and Pregnancy trial | 45 | 30 | 66.7 | NIH, MOH and EDCTP | Fieldworkers are given protocol-specific training and told about their responsibilities and objectives of the study. | Training/Career Development Program is in place to increase the number of qualified scientific and technical personnel capable of conducting independent medical research. This includes post-doctoral training-PhD levels, Parasitology, Cellular Biology, Pharmacology, Immunology; Maters of Sciences: Clinical research, Clinical Pharmacology, Public Health, Parasitology, Entomology, Nutrition. No specific framework for fieldworkers |
| 17. | **Research Institution 17-WA.** | 1998 | Maternal and child health, tropical diseases; malaria, Reproductive Health, Communicable diseases; Non-communicable diseases; human genetics; Health systems research, Social and Behavioural Studies, Poverty and Equity Studies | 263 | 193 | 73 | Ghana Health Service, Rockefeller Foundation, Population Council- USA, USAID | Fieldworkers training depends on the nature of the project, so for instance, if you are doing a survey on the health system, you will be taken through that and the overview of the overall Centre, community engagement and ethics. The project staff and the PI undertake the training | If a junior/frontline staff goes for further studies and has worked for 10 or more years and the field of study is relevant to their line of work, then the Centre, they may be supported. There is no formal structure to support fieldworkers. |
| 18. | **Research Institution 18-WA.** | 2007 | Molecular biology, immunology, clinical biology and bioinformatics | Staff are contracted from other organizations | FWs are contracted through other organization on a need basis then laid off at the end of a study | Depends on the project | National government, UNI-AID, Liverpool University, NIH and Fogarty foundation | New fieldworkers are usually given a Standard Operational Procedure then shown what they are going to do after that, they can be allowed to work in the field. | FWs are contracted through other organization on a need basis then laid off at the end of a study |
| 19. | ***Note: A discussion with MRC-UK and the INDEPTH secretariat in Ghana was also held with a view to crosscheck the information gathered from the interviews held** | | | | | | | | |
| **Total- 7 Research Institution from West Africa, 4 Research Institution from Southern Africa and 7 Research Institution from East Africa and 1 regional research coordination institution from Africa and 1 UK research funding institution** | | | | | | | | | |



For peer review only - http://bmjopen.bmj.com/site/about/guidelines.xhtml

### 3.2 **Data collection and analysis**

#### ***3.2.1 Phase 1: Telephone Interviews with managers and fieldworkers***

Out of the 18 directors contacted, each sent names of two individuals (1 senior fieldworker and 1 field manager) to be interviewed. We then contacted the proposed individuals and sought their individual consent to participate in a semi-structured interview, using telephone or Voice Over Internet Protocol (VOIP) communication. Interviews were conducted using an interviewed guide (Appendix A), that was developed in consultation with members of the study team and pre-tested prior to being used. In total, the 18 institutions that participated in the study employed a total workforce of 7839 staff, with 2505 (32.0%) being fieldworkers. Table 1 shows all the institutions that participated in the study, marked in dark blue (those that attended the workshop) and light blue (those that did not attend the workshop), with their names anonymized.

In total, 7 Research Institutions from West Africa, 4 from Southern Africa and 7 from East Africa participated in the interviews. In addition, individuals from 1 regional research coordination institution from Africa and 1 UK research funding organization were also interviewed. The Institutions conducted research in various fields including but not limited to Infectious tropical and bacterial diseases (9), Population Health and HIV studies (7), Social science and health systems research (9), Clinical Research/Drug and Vaccine trials (5), Epidemiology-Prevention and control of Malaria (9), Maternal and Newborn Health (6), Non-communicable diseases (3) fundamental sciences (1), human genetics (1), Molecular biology (1) and Clinical biology and bioinformatics (1). All the institutions received funding from external donors, mainly from the USA, United Kingdom (UK) and Canada. The number of staff employed per institution ranged from 45 to 1500 (mean 461), with the number of fieldworker ranging from 30 to 630 (mean 147).

Each telephone interview lasted for approximately one and a half hours. During the interviews, we sought to develop an understanding of current institutional practices and experiences in relation to training, support supervision and performance management of fieldworkers. Interviews with field managers explored issues related to processes of recruiting fieldworkers, the minimum qualification for fieldworkers, and the requirements for professional training as well as training given to fieldworkers. Additionally, issues around capacity building, support supervision and professional development for fieldworkers were discussed. Interviews with senior fieldworkers focused on their perceptions of the roles they play in research, challenges and mechanisms for addressing these challenges, career development opportunities and other existing support systems. The first author



For peer review only - http://bmjopen.bmj.com/site/about/guidelines.xhtml

conducted the interviews, which were digitally voice recorded, transcribed and immediately read through for verification and filling of any gaps by the interviewer before analysis.

.

### *3.2.2 Phase 2: The workshop with field managers*

A total of 25 managers from fifteen of the eighteen research Centres that took part in the phase one telephone interviews attended the 2 ½ days' workshop to further discuss the phase one findings. In total, the fifteen institutions represented at the workshop employed a total of 6702 staff, with 2136 (31.9%) being fieldworkers. The institutions came from nine different African countries. To attend the workshop, invitations were sent directly to the individuals who participated in phase one interviews and requested to seek clearance from their line managers before attending the workshop.

The workshop agenda was developed collaboratively between the study team and an independent Kenyan consultant who acted as the main workshop facilitator, and who had been selected based on his in-depth experience of participatory workshop methods and prior familiarity with research institutions in Africa. Overall, the workshop combined information sharing and large group discussions on day one with a series of participatory deliberative and consensus building activities over the following days. On day one, two sets of presentations were made and discussed in plenary. The first presentation described Phase one findings, with discussions on the range, strengths and challenges of fieldworkers' employment and support structures across institutions. The second presentation highlighted findings from earlier social science research in KWTRP and other research sites in Africa on the complex nature of fieldwork, including practical, social and ethical challenges that fieldworkers often face as part of their routine work, particularly within low-income communities and householders. Discussions that followed the presentations focused on the relevance and importance of these challenges across sites.

## 4. DATA ANALYSIS

Data analysis in this study followed a Framework Analysis approach (13). Transcripts were independently read and discussed in-depth by two members of the study team (FK and VM) to develop an initial coding framework, drawing on the main questions raised during interviews (see Appendix A) and themes that emerged during discussions. FK used Nvivo 10 software to organize data across the transcripts under these codes, adding codes for new issues emerging from the data. Data analysis charts were developed by FK and VM to account for the codes under themes by the participants, and support interpretation of patterns within the responses, drawing on the social science and research ethics literature.



For peer review only - http://bmjopen.bmj.com/site/about/guidelines.xhtml

Preliminary analysis of the telephone survey data was used to inform about the type and range of fieldworkers' support supervision and training practices as well as performance management strategies. The themes generated from this initial analysis were explored further and discussed in detail during the consultative workshop, with a view for consensus building and checking for similarities and variations across different research institutions. Emerging new themes were also discussed and explored using a combination of deliberative approaches, including large and small group discussions, round table and moderated discussions.

Moderators were careful to discuss ideas in detail without influencing the direction and outcome of the discussion. During the analysis, we compared and contrasted the fieldworkers' and field managers' views based on specific themes. Through this, we realized there were issues that fieldworkers and field managers shared the same views and others that they had different views. In this paper, we have tried to present these similarities and contracts together, rather than separately. We believe this will be much easier for our readers to follow than it would have been if we separated the views of fieldworkers from those of the field managers. Finally, although our participants are classified as fieldworkers and field managers, it is important to note that the same interview guide was used to interview both of them. This makes it logical to analyze and present these data together as shown in the next section

### 4.1 Data availability statement

Data are available in the form of transcripts that are currently archived in password protected computers. All the transcripts are anonymized, to avoid identification of the participants who were interviewed. Permission was obtained to use the data for purposes of this study only. No permission was granted to use the data for future research.

## 5. FINDINGS AND DISCUSSION

In this section, we describe and discuss findings from the telephone survey and where applicable, the highlights of the workshop, across three inter-related themes that were explored or emerged as important across this study. These themes are A) The current institutional structures and systems that support fieldworkers across institutions involved in the study; B) Participants' perceptions of work-related challenges for field workers and their relationship to support structures and; C) Views on the



For peer review only - http://bmjopen.bmj.com/site/about/guidelines.xhtml

ways in which challenges for fieldworkers might be better addressed, including agreement on institutional and wider recommendations.

### 5.1 Current Institutional structures and systems for fieldworkers

Across interviews and the subsequent workshop, it became clear that there is a large amount of diversity in the names, tasks and institutional structures that govern and support the way that fieldworkers operate in the institutions involved. For example, during interviews, senior fieldworkers described the existence of many different tasks and titles, including fieldworker, field enumerator, field officer, tracer, community technician and field assistant, which were often used interchangeably, within and across different centres. Similarly, the way that fieldworkers were recruited, contracted, trained and supported in their work was highly variable, again, between projects within an institution and between institutions. Table 1 describes these parameters across the research institutions involved.

As Table 1 shows, some form of in-house fieldworker training was common across institutions, with training mainly focused on the skills needed to undertake study-specific tasks, such as using particular data collection tools. In some cases, training in Good Clinical Practice (GCP) was also offered, particularly to support fieldworkers undertaking informed consent processes. This training was generally the responsibility of individual study teams within institutions. Four out of the fifteen institutions (27%) confirmed having a centrally coordinated training approach, three of which covered a more comprehensive set of topics including data collection skills, communication skills, informed consent processes, GCP and research ethics more generally. The other sites described conducting some kind of *ad hoc* training based on a specific study requirement or had no training plans for fieldworkers.

One area of fieldworker performance management which was rarely discussed and generally under-recognized across institutions was the concept of fieldworkers' professional career progression or continuing professional development. While such measures were largely routine for other cadres of research staff within institutions, they had not been applied to fieldworkers. Many felt this would be an important step towards recognizing the important role fieldworkers play, and in motivating them to maintain a high professional standard of work, but challenges were seen in sustaining such an initiative given the typically short-term contracts offered to fieldworkers, based on similarly short research funding cycles.

> *"It is good to establish a professional development scheme for fieldworkers. However, such a system may not be sustainable, given the dynamic employment terms of fieldworkers and*



For peer review only - http://bmjopen.bmj.com/site/about/guidelines.xhtml

*researcher in general…So although it is a good concept, it may be difficult to implement it [the scheme] within the context of research that relies on funding that is not always guaranteed"* (FWM, SA)

## 5.2 Participants' perceptions of work-related challenges for fieldworkers and their relationship to support structures

Across the following paragraphs, we describe the forms of challenges that fieldworkers and those working closely with them described during interviews, and that were taken forward for discussion at the workshop. In describing these, we highlight the types of institutional support that influence these challenges, including structural influences from human resource management strategies and research funding cycles, and more *ad hoc* often individual efforts to manage complex challenges on the ground.

### *5.2.1 Using different 'labels' for fieldworkers*

Discussions during interviews and at the workshop drew out differences in opinions on the importance of the 'labels' that were used to describe the work we describe here as 'fieldwork'. At a most pragmatic level, some fieldworkers felt that the most important issue was having a job, rather than being concerned about the title that was associated with that job. Practically, this attitude seems to hint at potentially problematic power imbalances for fieldworkers in relation to the institutions in which they were employed. Other fieldworkers felt that using different 'labels', particularly within one institution, could generate confusion about roles and lead to employment conditions being less transparent:

> *"It [roles taken by field workers] is not different. These are the organisation's terms…community technician, field worker, follow up staff, field technician…I mean it is just the same thing…it's just their [the organisation] way of making us look different, but we are the same"*. (SFW, KN)

Many field managers recognised the phenomenon of multiple labels as being used for slightly different forms of 'fieldwork'. There was, however, an initial divergence of opinion about the value of harmonising job titles. Some felt that this variation underlined important, even if small differences, in fieldworkers' current roles and prior experience or qualifications, which might not be obvious to all team members. Others felt there was little need for multiple titles, given the potential for confusion and conflict this might present. As will be described in the later section on recommendations, developing clear and strategic approaches to fieldworkers' roles within an institution emerged as an important feature of good practice for fieldworkers' employment. For many, harmonizing these structures and processes across institutions was also seen as valuable. Field managers felt harmonizing fieldworker employment terms, training and skills would promote cross-study and institution transfer



For peer review only - http://bmjopen.bmj.com/site/about/guidelines.xhtml

of fieldworkers and address the challenge associated with high turnover of fieldworkers due to short-term contracts.

### *5.2.2 Seeing fieldwork as a short-term activity*

A core tension for fieldworkers that was strongly expressed in interviews and the workshop was a general lack of recognition for the work that fieldworkers undertake within research institutions. Fieldworkers themselves saw their work as important, and as fundamentally contributing to answering important research questions and the reputation of institutions and careers of scientists and other cadres of research staff. There was, however, some frustration and perceived unfairness around the lack of opportunities for fieldworkers to progress within their area of work, or achieve recognition over sometimes periods of many years working in this role. Experiences were shared in which scientists who joined research centres were able to move ahead to higher positions through focused capacity building strategies, while fieldworkers' positions remained static. Some fieldworkers described working for an institution for more than ten years without more than basic training or promotion. Perhaps not surprisingly, given the way that 'fieldwork' has traditionally been viewed within research institutions (that is, as short-term and relatively non-technical work), the institutions involved in this study had not developed strategic approaches to fieldworkers' career development.

In response to these frustrations, some fieldworkers had taken up self-funded part-time training courses outside their existing jobs in the hope that this would allow them to progress within the institution. This signified the importance fieldworkers valued education and training as an important element of their career advancement. In practice, however, institutions did not have structures that would allow fieldworkers to move into roles in which any such new skills could be used. Against this background, most fieldworkers reported feeling demoralized and demotivated, including a feeling of low self-esteem and disillusionment:

> *"Once you are employed as a fieldworker, you will always be a fieldworker* [regardless of your qualifications]*". (SFW, GN)*

While some field managers supported this view, some argued that career development was a matter of personal motivation and determination. They explained how they [managers] had moved through the career ladder to become field managers, often starting their careers as junior fieldworkers and gaining extensive fieldwork experience along their career progression to their current positions, with the same limited training and support. Most managers had therefore been part of the system for a long time and were able to share their views both from the perspective of fieldworkers as well as field



For peer review only - http://bmjopen.bmj.com/site/about/guidelines.xhtml

managers hence extending the spectrum of experiences adduced and provide deeper insights to the issues that were raised

Given these challenges, which workshop participants saw as fundamentally unfair and as potentially importantly undermining the quality of research at its roots, participants agreed that fieldworkers should have greater recognition for the role they play within research institutions. They also agreed that it was important for institutions to support some form of career progression, for example, within a specialised category of staff supporting fieldwork. Providing fieldworkers with generic knowledge and skills on core research areas as part of such a scheme would enable them to transfer between studies at the end of a funding cycle. This would enable research centres to retain experienced fieldworkers, a policy that was thought likely to promote high scientific and ethical standards; and give fieldworkers more stability, skills and recognition.

### 5.2.3 Responding to complex challenges on the ground

Across this study, both during interviews and the subsequent workshop, fieldworkers and managers described often very difficult challenges that seem to be implicit in the nature of much field work. Some of these were practical, including the physical burdens of fieldwork, such as the experience of long working days with long distances to cover on foot, and risks of injury through dog or snake bites, and physical assault in less secure settings. In addition, many described emotional burdens, including anxiety, fear, sadness and sometimes helplessness associated with the nature of their work. This latter form of burden was described as being part of the nature of fieldwork, given common interactions with individuals and families who are facing often severe social and economic hardship. Within the community, fieldworkers were described as being seen as 'fellow community members' and, at the same time, the 'face' of the institutions that employ them, generating expectations of support from them on the part of community members visited (14).

These challenges have been well described in the literature on global health ethics, including from social science research at KWTRP, and our findings in this study underline the existence of these important challenges (1, 15-18). The fact that our study draws on experiences from 18 major research Centres in Africa and found similar challenges is an indication of how common and important those challenges are. Within this literature, there is increasing recognition of the moral work that fieldworkers are involved in during their everyday work (3, 19-21) which can directly impact the science and ethics of the studies of which they are part of (5, 22). For example, a key issue in the literature concerns the negotiation of informed consent processes, where fieldworkers balance perceived pressures to recruit participants according to targets set by managers and the extended period of time that may be needed to explain complex and often unfamiliar topics within consent



For peer review only - http://bmjopen.bmj.com/site/about/guidelines.xhtml

forms to householders;  and community members may feel that by agreeing to participate in studies, they will develop some leverage to access greater institutional support, including through their relationships with fieldworkers (4, 5, 23-25). Similar ethical challenges experienced by fieldworkers have been reported in high income settings, including the United States of America (USA), with a specific focus on how fieldworkers' and study coordinators' practices can affect protection of research participants (26). Although it could be argued that these settings are contextually different, it would be interesting to examine why fieldworkers in High Income Countries (HIC) experience the same ethical challenges as those in Africa, despite them being better trained, educated and resourced than their counterparts.(2, 26)

A particularly difficult situation that fieldworkers involved in routine Demographic Surveillance System (DSS) activities in this study described was the need to directly interact with bereaved families and seek information about deceased relatives as part of conducting verbal autopsies (interviews held with close family members of a recently deceased person to try to establish the cause of death)(27, 28) and other study-specific follow-ups. Such emotionally challenging interactions generated distress and worry for fieldworkers and could chronically undermine performance, leading to 'burnout':

> *"Yeah but you see what, after maybe going through these challenges sometimes they affect you or they affect your output" [SFW, SA]*
>
> *I felt bad, and you know the following day I was to go back to work, and there is no supportive counselling or something like that… So as fieldworkers sometimes we just burn out and sometimes with this kind of trauma, it becomes difficult…we go through a lot*" [SFW, KE]

During workshop discussions about support mechanisms to help fieldworkers address these emotional challenges, field managers and the fieldworkers were not able to identify existing strategies. Some managers described generally relying on fieldworkers' own personal judgements in handling these difficult situations.

> *"It is difficult to anticipate what challenges fieldworkers are likely to face…and even if they were to share them [the challenges] some of them are too complex to resolve. It is therefore easier to just let them [fieldworkers] solve them spontaneously and trust that they use enough wisdom to do the right thing"* [FWM, KE]

Experiences shared by fieldworkers revealed that the issues were usually quite complex and intertwined with cultural, structural and emotional sensitivities associated with the need to balance community and cultural expectation against the policies and guidelines of research institutions. As such, fieldworkers were often expected to strike a delicate balance, without knowledge of how



For peer review only - http://bmjopen.bmj.com/site/about/guidelines.xhtml

acceptable their actions were. Despite these actions having the potential to directly affect the scientific and ethical standards of data fieldworkers collect, structural support and referral systems were reportedly not available. This was well captured by one senior fieldworker who said.

> *"…. sometimes, you get that huruma [pity], you go into your pocket and give out something…if you have a human heart, sometimes there are some situations that are touching"* [SFW, SA].

During interviews and workshop discussions, there were no accounts of existing internal or external institutional mechanisms to support fieldworkers to specifically deal with these types of emotional challenges. Although effective support supervision and sharing these challenges during team meeting was identified as a potential avenue to identify strategies to deal with these challenges, the approaches used to supervise fieldworkers were noted to be significantly different across institutions, and not centrally coordinated within institutions, making it difficult to reflect on their effectiveness. Workshop participants therefore generally felt there was a gap in fieldworkers' support structures that should be addressed. These challenges were seen as occurring across all the institutions involved in the study.

### 5.3 Ways of strengthening institutional support

Based on the outputs of discussions described so far, two core institutional fieldworker support strategies were raised, discussed and agreed upon over the course of the workshop. The first concerned adapting the *content of skill building initiatives* to support fieldworkers to manage their interactions with community members and householders in ways that could support good scientific and ethical practice, seen from multiple perspectives. The second strategic support mechanism addressed the wider question of how professional support within institutions could support fieldworkers more *structurally* towards these aims, including aspects of performance management that commonly exist for other groups of research staff. Workshop participants agreed that 'performance management' in this respect would include aspects of recruitment, training, supervision, support, assessment and career or professional development. Specifically, delegates emphasized the need to strengthen fieldworkers' soft skills training to ensure they are well equipped to deal with cross-cutting issues around communication, community engagement, building trust and respect, which are fundamental in consenting and quality data collection. Fieldworkers themselves believed that more support would improve their skills, and accordingly the quality of the data collection. The need to develop a harmonized training curriculum, with core training modules across Africa was identified.

In this study, we noted a mismatch related to lack of appropriate training for fieldworkers; fieldworkers' limited level of education and the complexity of the tasks they are expected to



For peer review only - http://bmjopen.bmj.com/site/about/guidelines.xhtml

undertake, including but not limited to translating study information to study participants, understanding institutional and international ethical guidelines and applying these when interacting with study participants. These findings support the need for research institutions in Africa to invest in training and building the capacity of fieldworkers, in order to enhance the standards of the work fieldworkers undertake and provide optimum support for research.

### 5.4 Agreement on recommendations

Given the issues discussed in the preceding paragraphs around institutional fieldworkers' support structures, participants at the workshop agreed there was an urgent need to introduce more standardized and comprehensive approaches, focused on important areas that affect fieldworkers' performance and practices, including career progression, training/capacity building and management of emotional and ethical challenges. It was felt that strategies could build on the positive experiences of research institutions that had already begun to institutionalize such approaches. Participants recommended a pragmatic approach, aimed at empowering and building the knowledge and skills of fieldworkers, including processes to support continuous learning through the regular reflection of the practical and ethical issues they face in everyday experience. In this way, an important element of training and support supervision was described as enhancing skills to recognise and respond to these ethical issues. The specific recommendations made are described and discussed in the following paragraphs.

#### *5.4.1 Creating space for experience sharing and problem-solving*

One challenge identified during the survey was lack of psychological support after experiencing difficult and sometimes traumatic experiences, such as some interviewees breaking down during verbal-autopsy interviews, losing a study participant when close relationships had been built and even being physically attacked by emotional/aggrieved participants during interviews. Long working days with long distances to cover on foot, risks of injury through dog or snake bites, and physical assault in less secure settings were also said to be emotionally overburdening to fieldworkers. Documented evidence shows that stressful and traumatic experiences can alter the way people view themselves, the world around them, and alter how they process information and the way they behave and respond to the environment around them(29). A strategy described in the literature to address this challenge is the use of regular de-brief sessions, where fieldworkers are given opportunities to share experiences and consider the best approach to address challenges. During the workshop, participants recommended that field managers should create such opportunities for fieldworkers to talk about distressing or worrying issues encountered at work, through forms of feedback and reflection. This approach also requires a support supervision mechanism that is sensitive to the complex nature of the



For peer review only - http://bmjopen.bmj.com/site/about/guidelines.xhtml

experiences fieldworkers face. This supports Galas True's (2011) arguments that having access to colleagues to discuss issues and having effective support supervision acted as a buffering factor for research front-line staff who did not engage in ethical misconduct, despite facing the same pressures to meet recruitment goals and other stressors of conducting community-based research (30). Combining different ways of knowing and learning will enable fieldworkers to standardize the way they handle practical and ethical issues they encounter, even where these present as dilemmas with limited information on the best course of action. At the same time, in some cases, more traditional approaches to managing emotional distress, such as staff counselling, might also be needed.

#### *5.4.2 Increasing attention on soft skills for fieldworkers*

As reported by both fieldworkers and field managers, advancement has been made in the area of technical training for research staff, including fieldworkers. Good Clinical Practice, Health and Safety, Good Clinical Laboratory Practice (GCLP) training have become a standard approach for clinical study site preparation. While these trainings enable studies to adhere to international GCP/GCLP standards, they rarely equip fieldworkers with the necessary skills to enable them to deal with practical local challenges they face on the ground. There is, therefore, a need for fieldworkers to undergo more generic soft skills training, such as communication skills, basic psychology and counselling and community engagement technics to enable them to appreciate the practical local situations and develop skills to deal with the ground challenges.

#### *5.4.3 Having career development guidelines that are responsive to fieldworkers*

In a previous commentary that reported on the views of field managers who attended a deliberative workshop as part of this study, we presented important recommendations the managers made, including the need to i) increase institutional recognition of fieldworker's roles and the need for systematic and comprehensive capacity building to ensure adequate resources are allocated and that capacity building activities are well described in research proposals and grant applications; ii) develop common areas of a core curriculum for field worker capacity building to enhance quality of training processes and build their knowledge in basic biology, research approaches and methods, research ethics and research regulatory frameworks as well as data collection and documentation; respectful communication, and being aware of and managing ethical challenges and issues in practice; and iii) increase emphasis on fieldworkers' career development including developing regionally accredited training to support fieldworkers' professional development to increase individual motivation and enhance capacity for employment across different research organizations (12). These recommendations should be considered in order to address the challenges presented in this paper and



For peer review only - http://bmjopen.bmj.com/site/about/guidelines.xhtml

enhance the overall quality and integrity of research in Africa. Similar strategies have been proposed by Gala et al, to address challenges faced by community members employed as research staff in the USA (2)

## 6. CONCLUSIONS

The important role fieldworkers play in health research can be enhanced if fieldworkers are given adequate support. The challenges they face require a combination of knowledge, skills, attitude change and ethical values that can be enhanced through structured institutional support and instilling a sense of professional responsibility and moral values to fieldworkers; through ongoing support supervision, soft skills training and creating space for deliberative discussions and experience sharing. As stated by Alexander and Richman, strengthening the capacity of fieldworkers has the potential to increase the protection of research participants, enhance research integrity and promote the collection of valid data (31). Efforts to empower fieldworkers to make them better able to deal with these challenges should therefore continue. We realize that the results of this study may not be generalizable, as research institutions were selected based on specific context commonalities and association with the INDEPTH network and KWTRP. However, they do offer important insights on fieldworkers and field managers' views and experiences of fieldworkers' performance managing practices for institutions that come from similar settings as those that participated in this study. Although some of the proposed approaches such as soft-skills training, centralized coordination of fieldworkers training, fieldworkers' scheme of service and regular discussions to address fieldwork challenges have been ongoing in institutions such as the KWRTP, in Kenya, lack of documented evidence makes it difficult to say how effective and sustainable such approaches are. More research, therefore, needs to be done to explore the feasibility of the proposed strategies and generalizability of these study findings within a wider context in Africa. Finally, the proposed solutions may not solve all the challenges fieldworkers face, but they are certainly an important step towards improving the scientific and ethical standards of health research output from the African continent. The value of this study is not that every research centre in Africa can identify with every issue raised here, this would be difficult due to the variety of research centres in the continent, rather, we hope that by raising the issues highlighted here, even those centre not involved in the study will be provoked to examine their fieldworkers support system and see if any issues raised here apply to their setting. Furthermore, empirical studies should be done to identify which approaches are most effective in enabling research fieldworkers to deal with the myriad challenges they face. Such research will go a long way to improving the scientific and ethical standards research, not only in Africa but internationally, given the critical global role of Africa in research.



For peer review only - http://bmjopen.bmj.com/site/about/guidelines.xhtml

**7. REFERENCES**


1. Kamuya DM, Theobald SJ, Munywoki PK, Koech D, Geissler WP, Molyneux SC. Evolving friendships and shifting ethical dilemmas: fieldworkers' experiences in a short term community based study in Kenya. Dev World Bioeth. 2013;13(1):1-9.
2. True G, Alexander LB, Fisher CB. Supporting the role of community members employed as research staff: Perspectives of community researchers working in addiction research. Social Science & Medicine. 2017;187:67-75.
3. Kamuya DM, Theobald SJ, Marsh V, Parker M, Geissler WP, Molyneux SC. "The one who chases you away does not tell you go": silent refusals and complex power relations in research consent processes in Coastal Kenya. PloS one. 2015;10(5):e0126671.
4. Chantler T, Otewa F, Onyango P, Okoth B, Odhiambo F, Parker M, et al. Ethical Challenges that Arise at the Community Interface of Health R esearch: Village R eporters' Experiences in Western K enya. Developing world bioethics. 2013;13(1):30-7.
5. Kingori P. Experiencing everyday ethics in context: Frontline data collectors perspectives and practices of bioethics. Social science & medicine. 2013;98:361-70.
6. Madiega PA, Jones G, Prince RJ, Geissler PW. 'She's my sister-in-law, my visitor, my friend'– challenges of staff identity in home follow-up in an HIV trial in Western Kenya. Developing world bioethics. 2013;13(1):21-9.
7. Marshall PA, Marshall PL. Ethical challenges in study design and informed consent for health research in resource-poor settings: World Health Organization; 2007.
8. Richman KA, Alexander LB, True G. Proximity, ethical dilemmas, and community research workers. AJOB Primary Research. 2012;3(4):19-29.
9. Lansang MA, Dennis R. Building capacity in health research in the developing world. Bulletin of the World Health Organization. 2004;82(10):764-70.
10. Glickman SW, McHutchison JG, Peterson ED, Cairns CB, Harrington RA, Califf RM, et al. Ethical and scientific implications of the globalization of clinical research. New England Journal of Medicine. 2009;360(8):816-23.
11. Participants of an International Workshop in Kenya on the Role of Frontline Staff in Biomedical Research J, Kombe F. Enhancing quality and integrity in biomedical research in Africa: an international call for greater focus, investment and standardisation in capacity strengthening for frontline staff. BMC Med Ethics. 2015;16(1):77.
12. Kombe F. Enhancing quality and integrity in biomedical research in Africa: an international call for greater focus, investment and standardisation in capacity strengthening for frontline staff. BMC medical ethics. 2015;16(1):77.
13. Srivastava A, Thomson SB. Framework analysis: a qualitative methodology for applied policy research. 2009.
14. Molyneux S, Kamuya D, Madiega PA, Chantler T, Angwenyi V, Geissler PW. Field workers at the interface. Developing world bioethics. 2013;13(1):ii.
15. Kingori. Experiencing everyday ethics in context: frontline data collectors perspectives and practices of bioethics. Soc Sci Med. 2013;98:361-70.
16. Molyneux S, Kamuya D, Madiega PA, Chantler T, Angwenyi V, Geissler PW. Field workers at the interface. Developing world bioethics. 2013;13(1):ii-iv.
17. Chantler T, Otewa F, Onyango P, Okoth B, Odhiambo F, Parker M, et al. Ethical challenges that arise at the community interface of health research: village reporters' experiences in Western Kenya. Dev World Bioeth. 2013;13(1):30-7.
18. Angwenyi V, Kamuya D, Mwachiro D, Marsh V, Njuguna P, Molyneux S. Working with Community Health Workers as 'volunteers' in a vaccine trial: practical and ethical experiences and implications. Developing world bioethics. 2013;13(1):38-47.
19. Kamuya DM, Theobald SJ, Munywoki PK, Koech D, Geissler WP, Molyneux SC. Evolving Friendships and Shifting Ethical Dilemmas: Fieldworkers' Experiences in a Short Term Community Based Study in K enya. Developing world bioethics. 2013;13(1):1-9.




For peer review only - http://bmjopen.bmj.com/site/about/guidelines.xhtml

20. Wax ML. On fieldworkers and those exposed to fieldwork: Federal regulations and moral issues. Human Organization. 1977;36(3):321.

21. Zinn MB. Field research in minority communities: Ethical, methodological and political observations by an insider. Social problems. 1979;27(2):209-19.

22. Kombe F, Anunobi EN, Tshifugula NP, Wassenaar D, Njadingwe D, Mwalukore S, et al. Promoting Research Integrity in A frica: An African Voice of Concern on Research Misconduct and the Way Forward. Developing world bioethics. 2014;14(3):158-66.

23. Molyneux S, Kamuya D, Marsh V. Community members employed on research projects face crucial, often under-recognized, ethical dilemmas. The American Journal of Bioethics. 2010;10(3):24-6.

24. Mosavel M, Ahmed R, Daniels D, Simon C. Community researchers conducting health disparities research: Ethical and other insights from fieldwork journaling. Social science & medicine. 2011;73(1):145-52.

25. Simon C, Mosavel M. Community members as recruiters of human subjects: Ethical considerations. The American Journal of Bioethics. 2010;10(3):3-11.

26. Davis AM, Hull SC, Grady C, Wilfond BS, Henderson GE. The invisible hand in clinical research: The study coordinator's critical role in human subjects protection. The Journal of Law, Medicine & Ethics. 2002;30(3):411-9.

27. Zahr CA, Pinchuk M. Verbal autopsy standards: ascertaining and attributing cause of death: World Health Organization; 2007.

28. Fottrell E, Byass P. Verbal autopsy: methods in transition. Epidemiologic reviews. 2010;32(1):38-55.

29. Steele W. Trauma's Impact on Learning. Trauma and Loss: Research and Interventions. 2002.

30. True G, Alexander LB, Richman KA. Misbehaviors of front-line research personnel and the integrity of community-based research. J Empir Res Hum Res Ethics. 2011;6(2):3-12.

31. Alexander LB, Richman KA. Ethical Dilemmas in Evaluations Using Indigenous Research Workers. American Journal of Evaluation. 2008;29(1):73-85.

For peer review only



For peer review only - http://bmjopen.bmj.com/site/about/guidelines.xhtml

## Contributor and guarantor information

All authors were involved in conceptualizing the idea and contributed in writing the manuscript. Mr Francis Kombe (MPH), a male senior community facilitator with a wealth of experience in health research ethics, community engagement and communication skills, who worked as a fieldworker himself, before taking a managerial role at KWTRP, contributed to the planning, conduct, and reporting of the work described in the article and is responsible for the overall content. Prof Vicki Marsh (PhD), a female senior social scientist at KWTRP and Oxford University and Ms Dorothy Ikamba (Msc), a female career development officer at KWTRP, and career counsellor, assisted with reviewing the transcripts and the development of themes and sub-themes during the analysis. Prof Sassy Molyneux (PhD), a female senior social scientist at KWTRP and Oxford University, Dr Dorcas Kamuya (PhD), a female social scientist at KWTRP and Wellcome Trust Society & Ethics Fellow, and Dr Samson Kinyanju, a male immunologist and head of training at the KWTRP, contributed in designing the initial research ideas, reviewed the data collected and were involved in drafting the initial manuscript and edited subsequent versions. All authors reviewed and approved the final manuscript for publication. Francis Kombe accepts full responsibility for the work and the conduct of the study. He had full access to the data, and controlled the decision to publish. Francis Kombe attests that all listed authors meet authorship criteria and that no others meeting the criteria have been omitted.

## Copyright/license for publication

Francis Kombe has the right to grant on behalf of all authors and does grant on behalf of all authors, a worldwide license to the Publishers and its licensees in perpetuity, in all forms, formats and media (whether known now or created in the future), to i) publish, reproduce, distribute, display and store the Contribution, ii) translate the Contribution into other languages, create adaptations, reprints, include within collections and create summaries, extracts and/or, abstracts of the Contribution, iii) create any other derivative work(s) based on the Contribution, iv) to exploit all subsidiary rights in the Contribution, v) the inclusion of electronic links from the Contribution to third party material where-ever it may be located; and, vi) license any third party to do any or all of the above



For peer review only - http://bmjopen.bmj.com/site/about/guidelines.xhtml

## Transparency statement

Francis Kombe affirms that the manuscript is an honest, accurate, and transparent account of the study being reported; that no important aspects of the study have been omitted; and that any discrepancies from the study as originally planned have been explained.

## Competing interests

The views on this paper are the authors own viewpoints and do not represent the position of their organizations. The authors declare no conflict of interests.
The authors declared that they have no competing interests.

## Ethical approval

The paper is published with the permission of the Centre director, KEMRI. All the participant who participate in the telephone survey as well as those who attended the consultative workshop gave individual consent to participate in the study and the workshop, and permitted the publication of this manuscript. Permission to conduct this study was granted by a review conducted by the Centre for Geographic Medicine Research, Coast (CGMRC)- Centre Specific Committee and a final Ethical approval given by the Research and Scientific Review Unit (SERU) at the Kenya Medical Research Institute (KEMRI).

For peer review only



For peer review only - http://bmjopen.bmj.com/site/about/guidelines.xhtml

**DISCUSSION GUIDE FOR RESEARCH INSTITUTIONS WORKING WITH FIELDWORKERS (FWs) IN SSA**

**TITLE: STRENGTHENING TRAINING OF FRONT LINE (FWs) STAFF AS A MEANS TO ENHANCE COMMUNITY ENGAGEMENT**

**INFORMATION SHEET FOR MANAGERS AND SENIOR FRONTLINE STAFF**

## What is this survey all about?

This survey will be conducted through telephone/ teleconference discussion. One front line staff manager (people who have a direct role of providing support supervision to frontline staff) and one senior frontline staff member from a total of 20 health research institutions in Sub-Saharan Africa will be invited to participate in the discussion.

The aim of the discussion is to identify practical and ethical challenges experienced by frontline staff in their day to day activities and the available support supervision, performance management and Continuing Professional Development (CPD) practices within these institutions. Later, representatives from a few of the 20 institutions will be invited to a consultative workshop to follow up and discuss these issues in detail. The report generated from the survey and workshop will be shared with all institutes for their own use. In the long term, the report could be used to inform further discussions on the potential of developing a generic curriculum for frontline staff training.

## What is expected of you if you take part in this discussion?

If you agree to take part in this discussion, we will request you to select a date and time when you would prefer the moderator to call and have the discussion with you. We will then call and ask a few questions with regards to your perceptions of the roles of frontline staff and the institutional support supervision, monitoring and evaluation and general CPD practices. These questions are shown on the attached discussion guide for your attention. The discussion is expected to take a maximum of 30 minutes.

Participation in this interview is voluntary. While it is important to answer all the questions, feel free to skip any question that you do not feel comfortable to answer and the moderator will continue asking the next question. You can also decide to terminate the discussion at any time if you decide not to continue with the discussion for any reason.

## Is there any individual benefit for taking part in this discussion?

There is no individual benefit in taking part in this discussion. However, by sharing your experiences in working with frontline staff, you will assist in contributing to knowledge that could improve the way frontline staff deal with their practical and ethical challenges as well as their support supervision, performance and general CPD management practices in future.

## If you have any question

If you have any question about this survey, please contact any of the following people through the email addresses provided below

| | | |
|---|---|---|
| Francis Kombe<br>KWTRP<br>P.O BOX 230, KILIFI<br>Fkombe@kemri-wellcome.org | Sam Kinyanjui<br>KWTRP<br>P.O BOX 230, KILIFI<br>Skinyajui@kemri-wellcome.org | Dorcas Kamuya<br>KWTRP/ Ethox-Nuffield Department of Population Science, Oxford University<br>P.O BOX 230, KILIFI<br>Dkamuya@kemri-wellcome.org |

For peer review only - http://bmjopen.bmj.com/site/about/guidelines.xhtml

**DISCUSSION GUIDE FOR RESEARCH INSTITUTIONS WORKING WITH FIELDWORKERS (FWs) IN SSA**

**TITLE: STRENGTHENING TRAINING OF FRONT LINE (FWs) STAFF AS A MEANS TO ENHANCE COMMUNITY ENGAGEMENT**

## SECTION A: Institutional Background

1. Name of organization ________________________________________
2. Vision ________________________________________
3. Mission ________________________________________
4. Motto ________________________________________
5. Year organization was established [__][__][__] [__]
6. Source of main funding for the organization ________________________________________
7. Other funding agencies and collaborators ________________________________________
8. Types of research conducted at the organization: ________________________________________

## SECTION B: Frontline staff Managers' Perspective[1]

*This section explores managers' perceptions of the roles of and practical and ethical challenges faced by interface staff when discharging their duties and the institutional support supervision, performance management and Continuing Professional Development (CPD) practices*

1. What names are given to your frontline staff whose primary role involves recruiting and consenting research participants into studies?

2. What key roles/activities do they undertake in your organization?

3. In your institution, how many staff does it have (in total) and how many of these are employed as frontline staff?

4. What is the range of qualifications for frontline staff and what is their minimum level of education/qualification?

5. What is the nature of their employment e.g. are they in contract? What is the average contract period? Are the contracts renewable and what is considered during renewal? Any systems that ensure retention of frontline staff in the institutions?

6. What systems are in place to coordinate the way frontline staff are employed and retained across different studies in the institutions? What is the role of PI (if any) in retaining frontline staff?

7. What formal and informal processes are in place to induct new frontline staff when they first arrive, and to train them for their roles?

---

[1] Manager is the person whose primary responsibility involves working directly with front line staff

For peer review only

**DISCUSSION GUIDE FOR RESEARCH INSTITUTIONS WORKING WITH FIELDWORKERS (FWs) IN SSA**

**TITLE: STRENGTHENING TRAINING OF FRONT LINE (FWs) STAFF AS A MEANS TO ENHANCE COMMUNITY ENGAGEMENT**

8. How is the training for interface staff organized? Are there any refresher trainings/skills improvement activities? What are the *training approach eg frequency and objectives of the training,*

9. How is the frontline staff training managed? Describe funding, design, whether centralized or decentralized, and monitoring and evaluation of the training

10. Describe any practices, experiences and studies (if any) that have informed the current training eg feedback mechanisms; availability of a community engagement strategy, community Knowledge Attitude and Perception (KAP) surveys etc

11. How do you ensure skills learned are assimilated into the frontline's day to day activities? Are there any support supervision mechanisms, performance appraisals etc ? How are these implemented?

12. Describe any career progression scheme used by your organization for these staff groups

13. What challenges do you experience in working with and managing frontline staff?

## SECTION C: Frontline staffs' Experience-Staff perspective

*This section explores frontline staff's practical and ethical challenges faced as they discharge their duties and their perceptions of the institutional support in training and CPD*

1. What are roles and responsibilities of frontline staff?

2. What challenges do frontline staff face when interacting with community members? *Practical and ethical challenges/dilemmas faced, e.g. issues of confidentiality?*

3. What type of in-house and external training is given to frontline staff in your institution?
   - ***Which of these do you perceive to be most appreciated and for what reason?***

4. What are the perceived institutional challenges in supporting and capacity building of frontline staff?
   - ***How do frontline members of staff address the above challenges?***

## SECTION D: Planned Workshop

At the end of this survey, we hope to invite a selected number of the institutions to a 2 day workshop. Kindly indicate if you would like to be considered to attend this workshop.

For peer review only

# COREQ (COnsolidated criteria for REporting Qualitative research) Checklist

A checklist of items that should be included in reports of qualitative research. You must report the page number in your manuscript where you consider each of the items listed in this checklist. If you have not included this information, either revise your manuscript accordingly before submitting or note N/A.

| Topic | Item No. | Guide Questions/Description | Reported on Page No. |
|---|---|---|---|
| **Domain 1: Research team and reflexivity** | | | |
| *Personal characteristics* | | | |
| Interviewer/facilitator | 1 | Which author/s conducted the interview or focus group? | 22 |
| Credentials | 2 | What were the researcher's credentials? E.g. PhD, MD | 22 |
| Occupation | 3 | What was their occupation at the time of the study? | 22 |
| Gender | 4 | Was the researcher male or female? | 22 |
| Experience and training | 5 | What experience or training did the researcher have? | 22 |
| *Relationship with participants* | | | |
| Relationship established | 6 | Was a relationship established prior to study commencement? | 6 |
| Participant knowledge of the interviewer | 7 | What did the participants know about the researcher? e.g. personal goals, reasons for doing the research | 6 |
| Interviewer characteristics | 8 | What characteristics were reported about the inter viewer/facilitator? e.g. Bias, assumptions, reasons and interests in the research topic | 3, 10, 22 |
| **Domain 2: Study design** | | | |
| *Theoretical framework* | | | |
| Methodological orientation and Theory | 9 | What methodological orientation was stated to underpin the study? e.g. grounded theory, discourse analysis, ethnography, phenomenology, content analysis | 5 |
| *Participant selection* | | | |
| Sampling | 10 | How were participants selected? e.g. purposive, convenience, consecutive, snowball | 5 |
| Method of approach | 11 | How were participants approached? e.g. face-to-face, telephone, mail, email | 5 |
| Sample size | 12 | How many participants were in the study? | 5 |
| Non-participation | 13 | How many people refused to participate or dropped out? Reasons? | 5 |
| *Setting* | | | |
| Setting of data collection | 14 | Where was the data collected? e.g. home, clinic, workplace | 8 |
| Presence of non-participants | 15 | Was anyone else present besides the participants and researchers? | 5 |
| Description of sample | 16 | What are the important characteristics of the sample? e.g. demographic data, date | 6 |
| *Data collection* | | | |
| Interview guide | 17 | Were questions, prompts, guides provided by the authors? Was it pilot tested? | 8 |
| Repeat interviews | 18 | Were repeat inter views carried out? If yes, how many? | 8-10 |
| Audio/visual recording | 19 | Did the research use audio or visual recording to collect the data? | 8 |
| Field notes | 20 | Were field notes made during and/or after the inter view or focus group? | 8 |
| Duration | 21 | What was the duration of the inter views or focus group? | 8 |
| Data saturation | 22 | Was data saturation discussed? | |
| Transcripts returned | 23 | Were transcripts returned to participants for comment and/or | |

For peer review only

| Topic | Item No. | Guide Questions/Description | Reported on Page No. |
|---|---|---|---|
| | | correction? | |
| **Domain 3: analysis and findings** | | | |
| *Data analysis* | | | |
| Number of data coders | 24 | How many data coders coded the data? | 9 |
| Description of the coding tree | 25 | Did authors provide a description of the coding tree? | |
| Derivation of themes | 26 | Were themes identified in advance or derived from the data? | 9-10 |
| Software | 27 | What software, if applicable, was used to manage the data? | 9 |
| Participant checking | 28 | Did participants provide feedback on the findings? | 9 |
| *Reporting* | | | |
| Quotations presented | 29 | Were participant quotations presented to illustrate the themes/findings? Was each quotation identified? e.g. participant number | 10-16 |
| Data and findings consistent | 30 | Was there consistency between the data presented and the findings? | |
| Clarity of major themes | 31 | Were major themes clearly presented in the findings? | 10-16 |
| Clarity of minor themes | 32 | Is there a description of diverse cases or discussion of minor themes? | 10-16 |

Developed from: Tong A, Sainsbury P, Craig J. Consolidated criteria for reporting qualitative research (COREQ): a 32-item checklist for interviews and focus groups. *International Journal for Quality in Health Care*. 2007. Volume 19, Number 6: pp. 349 – 357

**Once you have completed this checklist, please save a copy and upload it as part of your submission. DO NOT include this checklist as part of the main manuscript document. It must be uploaded as a separate file.**

For peer review only

BMJ Open

# Enhancing Fieldworkers' Performance Management Support in Health Research: An exploratory study on the views of Field managers and Fieldworkers from major research centres in Africa

| | |
|---|---|
| Journal: | *BMJ Open* |
| Manuscript ID | bmjopen-2018-028453.R2 |
| Article Type: | Original research |
| Date Submitted by the Author: | 12-Aug-2019 |
| Complete List of Authors: | Kombe, Francis; Centre for Geographic Medicine Research Coast; African Research Integrity Network<br>Marsh, V; Centre for Geographic Medicine Research Coast; Centre for Tropical Medicine and Global Health, Nuffield Department of Medicine, Oxford University, UK<br>Molyneux, Sassy ; Centre for Geographic Medicine Research Coast; Centre for Tropical Medicine and Global Health, Nuffield Department of Medicine, Oxford University, UK<br>Kamuya, Dorcas; KEMRI Wellcome Trust Research Programme; Ethox Centre, Nuffield Department of Population Health, Oxford University, UK<br>Ikamba, Dorothy; KEMRI Wellcome Trust Research Programme<br>Kinyanjui, Samson; Centre for Geographic Medicine Research Coast; Centre for Tropical Medicine and Global Health, Nuffield Department of Medicine, Oxford University, UK |
| <b>Primary Subject Heading</b>: | Ethics |
| Secondary Subject Heading: | Ethics, Qualitative research |
| Keywords: | Fieldworkers, Africa, Capacity Building, Health Research, Research Ethics, Research Integrity |
| | |

SCHOLARONE™
Manuscripts

# Enhancing Fieldworkers' Performance Management Support in Health Research: An exploratory study on the *views of Field managers and Fieldworkers from major research centres in Africa*

Francis Kombe[1], Vicki Marsh[1,3], Sassy Molyneux[1,3], Dorcas Kamuya[1,2], Dorothy Ikamba[1], Kinyanjui Samson Muchina[1,3]

[1]KEMRI Wellcome Trust Research Programme, Kenya; [2]Ethox Centre, Nuffield Department of Population Health, Oxford University, UK; [3]Centre for Tropical Medicine and Global Health, Nuffield Department of Medicine, Oxford University, UK.

**Corresponding author**: Francis Kombe, KEMRI Wellcome Trust Research Programme, PO Box 230, Kilifi 80108, Kenya. Mobile +254 722459045; Email-Kajoleh@gmail.com

**Acknowledgements**: We acknowledge and greatly appreciate the support of all research institutions' Directors who allowed their field managers and field workers to take part in this project, and all the field managers and field workers who participated. We are extremely indebted to Prof Mike Parker, the Director of the Wellcome Centre for Ethics and Humanities and Director of Ethox Centre at the University of Oxford for his support and guidance during the project; and all the senior field managers and delegates who attended the consultative workshop. The manuscript is published with the support of the Director, KEMRI.

**Funding**:

This project was funded by Global Health Bioethics Network-UK (Wellcome Trust grant no. OXF-GHB02) and supplemented by KWTRP Capacity Building Strategic Award



For peer review only

For peer review only - http://bmjopen.bmj.com/site/about/guidelines.xhtml

(Wellcome Trust grant no 084538). The research and capacity building activities of the Global Health Bioethics Network are supported by a Wellcome Trust Strategic Award (096527)

**Keywords:** Africa, Fieldworkers, Research Integrity, Research Ethics, Capacity Building.

**Word Count: 7497**

For peer review only

**ABSTRACT**

**Introduction**

Fieldworkers are part of the system that promotes scientific and ethical standards in research, through data collection, seeking consent and providing support in research, due to their insider cultural knowledge and fluency in local languages. The credibility and integrity of health research therefore rely heavily on how this cadre of staff adheres to institutional and research procedures and guidelines.

**Study objectives**

This study mapped out existing practices in training, support and performance management of fieldworkers in Africa, described fieldworkers and their managers' experiences, and lessons learned. Consultative process, involving field managers from 15 international health research institutions, was used to identify appropriate ways of addressing the challenges fieldworkers working in Africa face.

**Methods**

**Phase one-** We conducted Thirty-two telephone interviews with Twenty field managers and Twelve senior fieldworkers from Eighteen major research Centres in Africa, MRC-UK and the INDEPTH Network Secretariat.

**Phase two-** We held a two-and-a-half days workshop involving Twenty-five delegates including Eighteen from the institutions that were involved in Phase One; and Seven additional stakeholders from the KWTRP. Data transcribed from the interviews and workshop proceedings were analyzed thematically using Nvivo 10 software.

**Results**

Most institutions employed fieldworkers, usually with Twelve years of formal education and residing within the geographic areas of research, to support studies. Although their roles were common, there were marked differences in the type of training, professional development schemes and fieldworkers support. Fieldworkers faced various challenges, with the potential to affect their ethical and scientific practices.

**Discussion**

Fieldworkers undertake vital tasks that promote the quality of data and ethical practice in research studies. There is, therefore, a need for research institutions to develop a structured support system, provide fieldworkers with interpersonal-skills training and provide space for




For peer review only - http://bmjopen.bmj.com/site/about/guidelines.xhtml

discussion, reflection and experience-sharing to help fieldworkers tackle the practical and ethical challenges they face.

For peer review only



For peer review only - http://bmjopen.bmj.com/site/about/guidelines.xhtml

**Strengths and limitations of this study**

**Strengths**

- Findings of this study add to a growing body of literature on how to enhance the quality and integrity of data collected with the support of fieldworkers; an important, but largely under supported and understudied cadre of staff, in international research settings in Africa. The fact that participants of this study were drawn from West, South and East Africa makes the findings of this study rich with an extended spectrum of experiences adduced, which provide deeper insights to the issues that were discussed.
- The use of consultative approaches, that allowed issues raised during telephone interviews to be followed up and discussed further in a workshop, provided a unique opportunity to cross-check data from fieldworkers with their managers, reflect on and unpack important issues, with a view to identifying relevant and applicable strategies for addressing challenges faced.

**Limitations**

- All interviews were done by phone. As such, important interview dynamics may have been missed. Face to face interviews would have been able to pick important non-verbal cues, which was not possible through telephone interviews. In addition, because our interviews focused on fieldworkers' challenges and potential opportunities, this could also have raised the fieldworkers' expectation to receive direct support to address the challenges they faced and could have contributed to overreporting of the challenges they experienced. Since the fieldworkers' managers reported similar challenges, we think this supports the validity of the fieldworkers data.
- The interviewer was a senior community facilitator at KWTRP and had worked as a fieldworker prior to being promoted to this position. It is possible that his personal experiences influenced his interpretation of the data. However, this was addressed through continuous discussions with two of the co-authors (VM and DK), including discussing the emerging themes from the data and reflecting on his positionality in the research process. This enabled him to reflect on his potential influence, in the data collection process, analysis and interpretation.
- Interviewees were nominated by Centre Directors, who were the primary contact people. Although individual consent was obtained from those nominated by the Directors prior to the interviews, it is possible that interviewees could have reported what they believed would be acceptable to their directors. During the interviews, we assured our participants about the confidentiality of the data they would share, which we think enabled them to share information sincerely and transparently. However, there is a potential that the power



For peer review only - http://bmjopen.bmj.com/site/about/guidelines.xhtml

differentials between the participants (field managers and fieldworkers), and the Centre Directors could have influenced their views and perceptions of the discussions held. Despite fieldworkers being hierarchically lower compared to field managers, and having different powers and authority within the research system, we could not identify any strong divergence in the views they shared. Furthermore, the institutions that participated in this study were selected on the basis of existing relationship with the INDEPTH Network and the KWTRP. It is possible that other institutions outside these networks had different experiences from those that participated in this study. We appreciate that this is an exploratory study. We believe the institutions involved shared a wide range of experiences and that findings from this study offer important insights for further research, especially for international health research institutions that employ fieldworkers.

For peer review only



For peer review only - http://bmjopen.bmj.com/site/about/guidelines.xhtml

## 1. BACKGROUND

High-quality data is a critical output of all science worldwide. Thus, research institutions put significant emphasis on generating data that are of the highest scientific and ethical standards. In many research programmes in low and middle-income countries, much of the data is collected by frontline staff referred to as "fieldworkers". This cadre of staff is also often responsible for recruiting potential research participants, administering consent forms and following up participants during studies (1). As such, beyond ensuring data integrity, fieldworkers play a key role in ensuring the ethical implementation of research protocols. The role of fieldworkers is particularly critical in international collaborative programmes with researchers drawn from outside local communities. In such settings, fieldworkers, who are normally from the local communities, can serve as the face of research programmes with potential to significantly influence communities' trust in research.

In carrying out these complex roles, fieldworkers face practical and ethical dilemmas that require the balancing of institutional roles and guidelines against community expectations and fieldworkers' personal interests (2). Hence, fieldworkers continuously make independent (often unconscious) decisions on how to apply 'ethical principles' on the ground. For example fieldworkers reported challenges such as dealing with 'silent refusals' (3) (where participants avoid key study procedures but do not openly refuse), and with many requests for health and other social- economic support that are not included in the study protocol (4-8).

### 1.1 Capacity Building and Investiment In Research

Over the years there has been an increasing interest and investment in developing research capacity in Africa and other low and middle income regions through long-term initiatives such as the WHO- Tropical Diseases Research Programme (TDR) (9), the European and Developing Countries Clinical Trials Initiative Partnership (EDCTP) and more recently, The Wellcome Trust-DFID funded "Developing Excellence in Leadership, Training and Science "scheme (DELTAS) Africa. Efforts to strengthen research capacity are likely to continue, as the trend towards greater cross-border data sharing, globalization of clinical research and comparability of data quality at the global level continues (10).

Given that fieldworkers play such a vital role in health research, it would be expected that research programmes, in addition to training scientists, would also focus on building fieldworkers' capacity to carry out their duties in the most effective manner. However, fieldworkers' capacity and skill sets have received relatively little attention. The mechanisms and processes by which research institutions in Africa support fieldworkers in undertaking their duties remain unclear. Apart from the Good Clinical Practice guidelines, which focus primarily on clinical trials, no other guidelines are available that provide a framework for the content and



For peer review only - http://bmjopen.bmj.com/site/about/guidelines.xhtml

approach for supporting the Continuing Professional Development and career growth of fieldworkers.

Understanding the challenges that fieldworkers face and the type of systems, and level of support research institutions in Africa provide, can enable us to identify opportunities for strengthening fieldworkers' scientific and ethical practices.

In this paper, we draw on our findings from a two-phase qualitative exploratory study on the perceptions, experiences and views of field managers and fieldworkers on the structural and performance management support extended to health research fieldworkers with the aim of informing institutional policies that promote research integrity and ethical practices for this critical cadre of staff (11).

## 2. STUDY OBJECTIVES

This study mapped out existing practices in relation to training, support and performance management of fieldworkers in Africa, fieldworkers' and their managers' experiences, and lessons learned. In addition, field managers from international health research institutions were involved in a consultative workshop to identify appropriate ways of addressing the challenges faced by fieldworkers working in research Centres in Africa.

## 3. METHODS

The study was planned over two phases, and focused on understanding structural and performance management practices and their implications within institutions. In Phase One, which was implemented in February 2014, we conducted semi-structured telephone interviews with Thirty-two fieldworker managers and experienced fieldworkers across Eighteen health research institutions in Fourteen countries in Africa and the United Kingdom. In Phase two, which was implemented in July 2014, we held a two and a half days workshop involving Twenty-five delegates. Eighteen of these delegates were from the institutions that were involved in phase I (i.e. one per institution). The workshop also included the Director of Training, three senior social scientists with long term experience in managing fieldworkers (also Co-PIs of the project), the Head of community engagement and two senior fieldworkers' supervisor from the KWTRP (12). Fieldworkers were not involved in the second phase.

This paper focuses on the results from Phase 1 (telephone interviews). Issues that were identified during Phase One were further discussed in more detail in the consultative workshop in order to build consensus, and identify common recommendations and strategies for addressing the reported challenges. Thus, there are certain sections of the paper that inevitably refer to views shared during the workshop. Results of the consultative workshop have already been published (12).



For peer review only - http://bmjopen.bmj.com/site/about/guidelines.xhtml

### 3.1 Patient and public involvement

Patients and the public were not involved in the design and conception of this study

### 3.2 Study site and selection of research institutions and interview participants.

This study was led by a team based at the Kenya Medical Research Institute (KEMRI) Wellcome Trust Research Programme (KWTRP) in Kenya. The KWTRP is a long-standing international health research institution with a wide range of existing scientific and capacity building collaborations with other research institutes in sub-Saharan Africa (see http://kemri-wellcome.org/). During the first phase, our sampling strategy for research institutions, and relevant individuals within these, focused on African institutions that manage Health and Demographic Surveillance Systems, given their employment of significant numbers of fieldworkers. We identified these institutions from our existing scientific and capacity building linkages, including the INDEPTH Network (http://www.indepth-network.org), an international research network for health and demographic surveillance sites in Africa which encompasses all research centers in sub-Saharan Africa that have Health Demographic Surveillance Systems. From the original list of 34 institutions obtained from the KWTP training department and INDEPTH site, we sent an email to the Directors, with an information sheet about the study, to invite them to participate in the study. A total of eighteen African international research institutions (see table 1) and the MRC-UK and the INDEPTH Network Secretariat in Ghana responded positively. A total of Sixteen Directors did not reply to our email and could not therefore determine why they did not respond. These were dropped after three reminders. The Eighteen African research institutions included a mix of institutions with varied years of operations that employed a diverse number of fieldworkers, engaged in a range of local and international collaborations and specialized in different research programmes/thematic areas. This mixture of characteristics was important in maximizing shared learning and experiences across institutions. In each institution, we wrote to the Director and sought permission to interview one senior fieldworker and one field manager. In the letter, we particularly underscored the need to select a manager whose primary responsibility involved working directly with and managing fieldworkers, including offering support supervision, training and other forms of performance management.

### 3.2 Data collection and analysis



For peer review only - http://bmjopen.bmj.com/site/about/guidelines.xhtml

#### 3.2.1 *Phase One: Telephone Interviews with managers and fieldworkers*

Out of the 34 Directors contacted, 18 sent names of two individuals (1 senior fieldworker and 1 field manager) to be interviewed. We then contacted the proposed individuals and sought their individual consent to participate in a semi-structured interview, using telephone or Voice Over Internet Protocol (VOIP) communication. Interviews were conducted using an interview guide (Appendix A), that was developed in consultation with members of the study team and pre-tested prior to being used. In total, the 18 institutions that participated in the study employed a total workforce of 7839 staff, with 2505 (32.0%) being fieldworkers. Table 1 shows all the institutions that participated in the study, marked in dark blue (those that attended the workshop) and light blue (those that did not attend the workshop), with their names anonymized.

Seven of the Research Institutions that participated in the interviews were from West Africa, four were from Southern Africa and Seven were from East Africa. In addition, individuals from one regional research coordination institution from Africa and one UK research funding organization were also interviewed. The Institutions conducted research in various fields including but not limited to Infectious tropical and bacterial diseases (n=9), Population Health and HIV studies (n=7), Social science and health systems research (n=9), Clinical Research/Drug and Vaccine trials (n=5) Epidemiology-Prevention and control of Malaria (n=9), Maternal and Newborn Health (n=6), Non-communicable diseases (n=3) fundamental sciences (n=1), human genetics (n=1), Molecular biology (n=1) and Clinical biology and bioinformatics (n=1). All the institutions received funding from external donors, mainly from the USA, United Kingdom (UK) and Canada. The number of staff employed per institution ranged from 45 to 1500 (mean 461), with the number of fieldworkers ranging from 30 to 630 (mean 147).



For peer review only - http://bmjopen.bmj.com/site/about/guidelines.xhtml

# Table 1: Main characteristics of Institutions Involved in the project

| No | Name of Institution | Year Established | The main type of research | Number of Staff | Number of fieldworkers | % of FWs | Source of Funding | Description of training given to Fieldworkers | Description of career progressing system used |
|---|---|---|---|---|---|---|---|---|---|
| 1. | **Research Institution 1-WA.** | 1947 | Infectious tropical diseases | 1104 | 301 | 27.3 | MRC, UK | CORE training is given to all fieldworkers with three level, including basic, intermediary and advanced. Everybody who gets employed as a fieldworker must go through core training. | Successful completion of fieldworker straining is automatically awarded one salary grade promotion and allows fieldworkers to move through a career progression from A2 A3, B1 B2 B3, C1 C2 C3, D1 D2 D3 and E1 E2 E3. Some fieldworkers move up to C2 after the training because of the change in job description and responsibilities. |
| 2. | **Research Institution 2-SA.** | 1997 | Health and Population, HIV studies, | 800 | 52 | 5.4 | WT, NIH & EGPAF | A mass training is done for 5 weeks: 4 weeks course work and 1 week practical. The test is done to assess skills gained and certificate issued upon passing the exams. Those who pass are put on a waiting list and employed directly to studies when a vacancy arises. | A professional development process involving all staff is available. It is not specific to fieldworkers. |
| 3. | **Research Institution 3-WA.** | 1983 | Anthropology, Demography, Health Economics, Clinical trials | 153 | 40 | 26.1 | NIH and USAID, WHO & EGAD, | A centrally managed training coordinated by a social scientist who takes through the fieldworkers on how to approach the community is usually given | Fieldworkers are usually outsourced. No scheme of service is available |
| 4. | **Research Institution 4-EA.** | 1970 | Infectious diseases, prevention and control | 120 | 40 | 33.3 | NORAD, SIDA, EDCTP, WT, BMGF, WHO, TDR, GLOBVAC, Swiss TPH, | New staff are trained on the specific project and other training such as GCP, Data Management and Ethics. | There is no career ladder but if they improve their educational qualification, they get an opportunity to apply for other higher posts |
| 5. | **Research Institution 5-SA.** | 1996 | Malaria, HIV AIDS, Vaccine Trials, Pneumonia, Bacterial infection | 200 | 100 | 50.0 | AECID, MOH & EDPTP | All fieldworkers undergo project-related training, GCP, Data Management and Ethics upon being employed | None |
| 6. | **Research Institution 6-EA** | 1989 | Immunology, TB, Bacteremia, HIV, Social Science, Health Economics, | 750 | 300 | 40 | WT, IAVI, NIH | Fieldworkers training is centrally coordinated. All new fieldworkers undergo training in communication skills, research ethics, introduction to medical research, GCP and phlebotomy before being allowed to work in the field | A career progression framework for fieldworkers exists, which stipulate the entry point and career progression subject to attainment of job experience, in-house training as well as external training which could either be self or programme sponsored |
| 7. | **Research Institution 7-EA** | 1998 | Health system research, Maternal and Newborn Health & HIV | 700 | 135 | 19.3 | DFID, INDEPTH, MOH, IDRC, DFID, RF, BMGF, FP7, USAID & CDC-RBM- | Field enumerators who work in the DSS go through a refresher training every six months to share the challenges from the previous round and train them on new issues, discuss the challenges and how to address them we also remind the fieldworkers on the ethical issues for example about their own responsibilities | An interviewer and field staff can start as a field interviewer and then be a supervisor and then sometimes field manager. HR have their own arrangement but the structure is not formal |
| 8. | **Research Institution 8-EA** | 1996 | Malaria, HIV, Health and Poverty, TB | 800 | 183 | 22.9 | MOH, Swiss Institute, BMG, EU | FWs are given intensive training about how to work in the DSS and the procedures of doing the DSS activities. We also train them about ethics and their responsibilities. At the same time, other related issues concerning DSS activities are varied. | The training unit deals with all the training needs and capacity building. So a fieldworker can even be appointed to be a field supervisor or a field manager if they express interest. A structure is available |
| 9. | **Research Institution 9-EA** | 2011 | Health system research, Reproductive health | 250 | 100 | 75.6 | Wellcome Trust | New fieldworkers are paired with more experienced fieldworkers for a given period of time. They are also required to attend mandatory meetings to share their experiences. After every DSS round, a three days' refresher training is organized to recap on what they have done and solve any problems. | Training and capacity building is available for senior research staff but not for fieldworkers |
| 10. | **Research Institution 10-WA.** | 2005 | social scientist and clinical research | 90 | 50 | 50.0 | MOH & LSHTM | Training for field workers is coordinated by the leader of the study team so for every study, we have a team responsible for coordinating the fieldworkers training, who train them about field practices, community entry law and so on. | There is no formal structure for fieldworkers' career development. Only those with a degree and Masters qualification can be promoted to senior positions |
| 11. | **Research Institution 11-WA.** | 1994 | Malaria, TB, HIV, and Maternal and child health | 200 | 100 | 50.0 | LSHTM, The Kwame Nkrumah University of science and technology, University of Ghana | FWs receive training retraining on the forms they use in the field, at the end of the round and at the end of the year. Every week, they meet with their supervisors in the field to discuss any emerging issues. | Fieldworkers can be supported to undertake an advanced training in nursing or laboratory but there is no guarantee that their position would be reserved when they complete the course. A formal structure is not available |
| 12. | **Research Institution 12-EA.** | 1984 | Malaria, TB, HIV and emerging infection | 1500 | 630 | 42.0 | CDC, EDCTP and other agencies | Fieldworkers are trained on ethics, which entails Good Clinical Practice then they are paired up with more experienced ones and then trained on relevant processes and specific study document, tools and protocol training, general orientation, confidentiality issues and after this, they are left to | There are promotions on a yearly basis when a staff is being evaluated and other conditions which the staff should meet before the supervisor would recommend they get a promotion. No career progression framework for fieldworkers |
| 13. | **Research Institution 13-EA** | 1988 | Maternal and Newborn health | 380 | 105 | 27.6 | MRC UK | All fieldworkers undergo a GCP training and then trained on their specific study protocol | Career progression depends on studies. Different studies have different requirement for a career, some of them may directly be organized by the program for a certain cadre to go for training. We don't have a structure of career progression for fieldworkers |
| 14. | **Research Institution 14-SA** | 1995 | HIV and AIDS, Malaria, Non-communicable diseases | 300 | 73 | 24.3 | WT & EU | There is no standard structure. Once employed and taken through HR induction and study protocol, they are ready to go and work. There is no | No structure available but if there is a new position, a fieldworker who has the appropriate qualification can apply. There is no formal approach to supporting |

| | | | | | | | | | |
|---|---|---|---|---|---|---|---|---|---|
| | | | | | | | | process to build FWs capacity to address practical and ethical challenges FWs face. | fieldworkers. Sponsorship is competitive against all staff members |
| 15. | **Research Institution 15-SA** | 1979 | TB, HIV, NCD, Clinical and epidemiological research | 184 | 73 | 39.7 | WT, LSHTM, BMGF & EU | Fieldworkers get trained on demographic surveillance its basic outline and principles because most studies are linked to our Continuous Registration System (CRS). They are also trained on HIV counselling and testing and data entry. | No formal career path exists |
| 16. | **Research Institution 16-WA** | 1992 | Vaccine and Drug trials, fundamental sciences, Mosquitoes genetics, Parasites genetics, Drug Resistance (PCR, in Vitro tests…), Malaria Immunology, Malaria and Pregnancy trial | 45 | 30 | 66.7 | NIH, MOH and EDCTP | Fieldworkers are given protocol-specific training and told about their responsibilities and objectives of the study. | Training/Career Development Program is in place to increase the number of qualified scientific and technical personnel capable of conducting independent medical research. This includes post-doctoral training-PhD levels, Parasitology, Cellular Biology, Pharmacology, Immunology; Maters of Sciences: Clinical research, Clinical Pharmacology, Public Health, Parasitology, Entomology, Nutrition. No specific framework for fieldworkers |
| 17. | **Research Institution 17-WA.** | 1998 | Maternal and child health, tropical diseases; malaria, Reproductive Health, Communicable diseases; Non-communicable diseases; human genetics; Health systems research, Social and Behavioural Studies, Poverty and Equity Studies | 263 | 193 | 73 | Ghana Health Service, Rockefeller Foundation, Population Council- USA, USAID | Fieldworkers training depends on the nature of the project, so for instance, if you are doing a survey on the health system, you will be taken through that and the overview of the overall Centre, community engagement and ethics. The project staff and the PI undertake the training | If a junior/frontline staff goes for further studies and has worked for 10 or more years and the field of study is relevant to their line of work, then the Centre, they may be supported. There is no formal structure to support fieldworkers. |
| 18. | **Research Institution 18-WA.** | 2007 | Molecular biology, immunology, clinical biology and bioinformatics | Staff are contracted from other organizations | FWs are contracted through other organization on a need basis then laid off at the end of a study | Depends on the project | National government, UNI-AID, Liverpool University, NIH and Fogarty foundation | New fieldworkers are usually given a Standard Operational Procedure then shown what they are going to do after that, they can be allowed to work in the field. | FWs are contracted through other organization on a need basis then laid off at the end of a study |
| 19. | ***Note: A discussion with MRC-UK and the INDEPTH secretariat in Ghana was also held with a view to crosscheck the information gathered from the interviews held** | | | | | | | | |
| **Total- 7 Research Institution from West Africa, 4 Research Institution from Southern Africa and 7 Research Institution from East Africa and 1 regional research coordination institution from Africa and 1 UK research funding institution** | | | | | | | | | |



For peer review only - http://bmjopen.bmj.com/site/about/guidelines.xhtml

Each telephone interview lasted for approximately one and a half hours. During the interviews, we sought to develop an understanding of current institutional practices and experiences in relation to training, support supervision and performance management of fieldworkers. Interviews with field managers explored issues related to processes of recruiting fieldworkers, the minimum qualification for fieldworkers, and the requirements for professional training as well as internal and external training given to fieldworkers. Additionally, issues around capacity building, support supervision and professional development for fieldworkers were discussed. Interviews with senior fieldworkers focused on their perceptions of the roles they play in research, challenges and mechanisms for addressing these challenges, career development opportunities and other existing support systems. The first author conducted the interviews, which were voice recorded, transcribed and immediately read through for verification and filling of any gaps by the interviewer before analysis.

### *3.2.2 Phase 2: The workshop with field managers*

A total of Twenty-five managers from fifteen of the eighteen research Centres that took part in the Phase One telephone interviews attended the Two and a half days' workshop to further discuss the Phase One findings. In total, the fifteen institutions represented at the workshop employed a total of 6702 staff, with 2136 (31.9%) being fieldworkers. The institutions came from nine different African countries. To attend the workshop, invitations were sent directly to the individuals who participated in Phase One interviews and requested to seek clearance from their line managers before attending the workshop.

The workshop agenda was developed collaboratively between the study team and an independent Kenyan consultant who acted as the main workshop facilitator, and who had been selected based on his in-depth experience of participatory methods and prior familiarity with research institutions in Africa. Overall, the workshop combined information sharing and large group discussions on day one with a series of participatory consultative and consensus building activities over the following days. On day one, two sets of presentations were made and discussed in plenary. The first presentation described Phase One findings, with discussions on the range, strengths and challenges of fieldworkers' employment and support structures across institutions. The second presentation highlighted findings from earlier social science research in KWTRP and other research sites in Africa on the complex nature of fieldwork, including practical, social and ethical challenges that fieldworkers often face as part of their routine work,



For peer review only - http://bmjopen.bmj.com/site/about/guidelines.xhtml

particularly within low-income communities and householders. Discussions that followed the presentations focused on the relevance and importance of these challenges across sites.

For peer review only



For peer review only - http://bmjopen.bmj.com/site/about/guidelines.xhtml

# 4. DATA ANALYSIS

Data analysis in this study followed a Framework Analysis approach (13). Transcripts were independently read and discussed in-depth by two members of the study team (FK and VM) to develop an initial coding framework, drawing on the main questions raised during interviews (see Appendix A) and themes that emerged during discussions. FK used Nvivo 10 software to organize data across the transcripts under these codes, adding codes for new issues emerging from the data. Data analysis charts were developed by FK and VM to account for the codes under themes by the participants, and support interpretation of patterns within the responses, drawing on the social science and research ethics literature.

Preliminary analysis of the telephone survey data was used to inform about the type and range of fieldworkers' support supervision and training practices as well as performance management strategies. The themes generated from this initial analysis were explored further and discussed in detail during the consultative workshop, with a view for consensus building and checking for similarities and variations across different research institutions. Emerging new themes were also discussed and explored using a combination of qualitative participatory approaches, including large and small group discussions, round table and moderated discussions.

Moderators were careful to discuss ideas in detail without influencing the direction and outcome of the discussion. During the analysis, we compared and contrasted the fieldworkers' and field managers' views based on specific themes. Through this, we realized there were issues that fieldworkers and field managers shared the same views and others that they had different views. In this paper, we have tried to present these similarities and contracts together, rather than separately. We believe this will be much easier for our readers to follow than it would have been if we separated the views of fieldworkers from those of the field managers. Finally, although our participants are classified as fieldworkers and field managers, it is important to note that questions in the interview guides that were used to interview them were very similar. This makes it logical to analyze and present these data together as shown in the next section

# 5. FINDINGS

In this section, we describe and discuss findings from the telephone survey and where applicable, the highlights of the workshop, across three inter-related themes that were explored or emerged as important across this study. These themes are A) The current institutional structures and systems that support fieldworkers across the institutions involved in the study;



For peer review only - http://bmjopen.bmj.com/site/about/guidelines.xhtml

B) Participants' perceptions of work-related challenges for fieldworkers and their relationship to support structures and; C) Views on the ways in which challenges for fieldworkers might be addressed, including agreement on institutional and wider recommendations.

**5.1 Current Institutional structures and systems for fieldworkers**

Across interviews and the subsequent workshop, it became clear that there is a large amount of diversity in the names, tasks and institutional structures that govern and support the way that fieldworkers operate in the institutions involved. For example, during interviews, senior fieldworkers described the existence of many different tasks and titles, including fieldworker, field enumerator, field officer, tracer, community technician and field assistant, which were often used interchangeably, within and across different Centres. Similarly, the way that fieldworkers were recruited, contracted, trained and supported in their work was highly variable, between projects within an institution and between institutions. Table 1 describes these parameters across the research institutions involved.

As Table 1 shows, some form of in-house fieldworkers training was common across the institutions, with training mainly focused on the skills needed to undertake study-specific tasks, such as using particular data collection tools. In some cases, training in Good Clinical Practice (GCP) was also offered, particularly to support fieldworkers undertaking informed consent processes. Often, such training was generally the responsibility of individual study teams within institutions. Four out of the fifteen institutions (27%) confirmed having a centrally coordinated training approach, three of which covered a more comprehensive set of topics including data collection skills, communication skills, informed consent processes, GCP and research ethics more generally. The other sites described conducting some kind of *ad hoc* training based on a specific study requirement or had no training plans for fieldworkers.

One area of fieldworker performance management which was rarely discussed and generally under-recognized across institutions was the concept of fieldworkers' professional career progression or continuing professional development. While such measures were largely routine for other cadres of research staff within institutions, they had not been applied to fieldworkers. Many felt this would be an important step towards recognizing the important role fieldworkers play, and in motivating them to maintain a high professional standard of work, but challenges were seen in sustaining such an initiative given the typically short-term contracts offered to fieldworkers, based on similarly short research funding cycles.



For peer review only - http://bmjopen.bmj.com/site/about/guidelines.xhtml

> *"It is good to establish a professional development scheme for fieldworkers. However, such a system may not be sustainable, given the dynamic employment terms of fieldworkers and researcher in general…So although it is a good concept, it may be difficult to implement it [the scheme] within the context of research that relies on funding that is not always guaranteed"* (FWM, SA)

## 5.2 Participants' perceptions of work-related challenges for fieldworkers and their relationship to support structures

Across the following paragraphs, we describe the forms of challenges that fieldworkers and those working closely with them described during interviews, and that were taken forward for discussion at the workshop. In describing these, we highlight the types of institutional support that influence these challenges, including structural influences from human resource management strategies and research funding cycles, and more *ad hoc* often individual efforts to manage complex challenges on the ground.

### *5.2.1 Using different 'labels' for fieldworkers*

Discussions during interviews and at the workshop drew out differences in opinions on the importance of the 'labels' that were used to describe the work we describe here as 'fieldwork'. At a most pragmatic level, some fieldworkers felt that the most important issue was having a job, rather than being concerned about the title that was associated with that job. Practically, this attitude seems to hint at potentially problematic power imbalances for fieldworkers in relation to the institutions in which they were employed. Other fieldworkers felt that using different 'labels', particularly within one institution, could generate confusion about roles and lead to employment conditions being less transparent:

> *"It [roles taken by field workers] is not different. These are the organisation's terms…community technician, field worker, follow up staff, field technician…I mean it is just the same thing…it's just their [the organisation] way of making us look different, but we are the same"*. (SFW, KN)

Many field managers recognised the phenomenon of multiple labels as being used for slightly different forms of 'fieldwork'. There was, however, an initial divergence of opinion about the value of harmonising job titles. Some felt that this variation underlined important, even if small differences, in fieldworkers' current roles and prior experience or qualifications, which might not be obvious to all team members. Others felt there was little need for multiple titles, given



For peer review only - http://bmjopen.bmj.com/site/about/guidelines.xhtml

the potential for confusion and conflict this might present. As will be described in the later section on recommendations, developing clear and strategic approaches to fieldworkers' roles within an institution emerged as an important feature of good practice for fieldworkers' employment. For many, harmonizing these structures and processes across institutions was also seen as valuable. Field managers felt harmonizing fieldworker employment terms, training and skills would promote cross-study and institution transfer of fieldworkers and address the challenge associated with high turnover of fieldworkers due to short-term contracts.

### *5.2.2 Seeing fieldwork as a short-term activity*

A core tension that was strongly expressed in interviews and the workshop was a general lack of recognition for the work that fieldworkers undertake within research institutions. Fieldworkers themselves saw their work as important, and as fundamentally contributing to answering important research questions, promoting the reputation of institutions, careers of scientists and other cadres of research staff. There was, however, some frustration and perceived unfairness around the lack of opportunities for fieldworkers to progress within their area of work, or achieve recognition over sometimes periods of many years working in this role. Experiences were shared in which scientists who joined research centres were able to move ahead to higher positions through focused capacity building strategies, while fieldworkers' positions remained static. Some fieldworkers described working for an institution for more than ten years without more than basic training or promotion. Perhaps not surprising, given the way that 'fieldwork' has traditionally been viewed within research institutions (that is, as short-term and relatively non-technical work), the institutions involved in this study had not developed strategic approaches for fieldworkers' career development.

In response to these frustrations, some fieldworkers had taken up self-funded part-time training courses outside their existing jobs in the hope that this would allow them to progress within the institution. This signified the importance fieldworkers valued education and training as an important element of their career advancement. In practice, however, institutions did not have structures that would allow fieldworkers to move into roles in which such new skills could be used. Against this background, most fieldworkers reported feeling demotivated, including a feeling of low self-esteem and disillusionment:

> *"Once you are employed as a fieldworker, you will always be a fieldworker* [regardless of your qualifications]". *(SFW, GN)*

While some field managers supported this view, some argued that career development was a matter of personal motivation and determination. They explained how they [managers] had moved through the career ladder to become field managers, often starting their careers as junior fieldworkers and gaining extensive fieldwork experience along their career progression to their



For peer review only - http://bmjopen.bmj.com/site/about/guidelines.xhtml

current positions, with the same limited training and support. Most managers had therefore been part of the system for a long time and were able to share their views both from the perspective of fieldworkers as well as field managers hence extending the spectrum of experiences adduced and provide deeper insights to the issues that were raised

Given these challenges, which workshop participants saw as fundamentally unfair and as potentially importantly undermining the quality of research at its roots, participants agreed that fieldworkers should have greater recognition for the role they play within research institutions. They also agreed that it was important for institutions to support some form of career progression, for example, within a specialised category of staff supporting fieldwork. Providing fieldworkers with generic knowledge and skills on core research areas as part of such a scheme would enable them to transfer between studies at the end of a funding cycle. This would enable research centres to retain experienced fieldworkers, a policy that was thought likely to promote high scientific and ethical standards; and give fieldworkers more stability, skills and recognition.

### 5.2.3 Responding to complex challenges on the ground

In both the telephone interviews and the subsequent workshop, fieldworkers and managers described facing often very difficult challenges that seem to be implicit in the nature of much field work. Some of these were practical, including the physical burdens of fieldwork, such as the experience of long working days with long distances to cover on foot, risks of injury through dog or snake bites, and physical assault in less secure settings. Many also described emotional burdens including anxiety, fear, sadness and sometimes helplessness when interacting with individuals and families facing severe social and economic hardship. Within the community, fieldworkers said they were perceived as 'fellow community members' and, at the same time, the 'face' of the institutions that employ them, generating expectations of support that were beyond their mandates from the community members they visited (14).

These challenges have been well described in the literature on global health ethics, including from social science research at KWTRP, and our findings in this study underline the existence of these important challenges (1, 15-18). The fact that our study draws on experiences from 18 major research Centres in Africa and found similar challenges is an indication of how common and important those challenges are. Within this literature, there is increasing recognition of the moral work that fieldworkers are involved in during their everyday work (3, 19-21) which can directly impact the science and ethics of the studies they work for (5, 22). For example, a key issue in the literature concerns the negotiation of informed consent processes, where fieldworkers balance perceived pressures to recruit participants according to targets set by managers and the extended period of time that may be needed to explain complex and often



For peer review only - http://bmjopen.bmj.com/site/about/guidelines.xhtml

unfamiliar topics within consent forms to household members. In addition, community members may feel that by agreeing to participate in studies, they will develop some leverage to access greater institutional support, including through their relationships with fieldworkers (4, 5, 23-25). Similar ethical challenges experienced by fieldworkers have been reported in high income settings, including the United States of America (USA), with a specific focus on how fieldworkers' and study coordinators' practices can affect protection of research participants (26). Although it could be argued that these settings are contextually different, it would be interesting to examine why fieldworkers in High Income Countries (HIC) experience the same ethical challenges as those in Africa, despite them being better trained, educated and resourced than their counterparts.(2, 26)

A particularly difficult situation that fieldworkers involved in routine Demographic Surveillance System (DSS) activities in this study described was the need to directly interact with bereaved families and seek information about deceased relatives as part of conducting verbal autopsies (interviews held with close family members of a recently deceased person to try to establish the cause of death)(27, 28). Such emotionally challenging interactions generated distress and worry for fieldworkers and could chronically undermine performance, leading to 'burnout':

> *"Yeah but you see what, after maybe going through these challenges sometimes they affect you or they affect your output" [SFW, SA]*
>
> *I felt bad, and you know the following day I was to go back to work, and there is no supportive counselling or something like that… So as fieldworkers sometimes we just burn out and sometimes with this kind of trauma, it becomes difficult…we go through a lot*" [SFW, KE]

During workshop discussions about support mechanisms to help fieldworkers address these emotional challenges, field managers were not able to identify existing strategies; as was also reported by fieldworkers during the telephone interviews. Some managers described generally relying on fieldworkers' own personal judgements in handling these difficult situations.

> *"It is difficult to anticipate what challenges fieldworkers are likely to face…and even if they were to share them [the challenges] some of them are too complex to resolve. It is therefore easier to just let them [fieldworkers] solve them spontaneously and trust that they use enough wisdom to do the right thing"* [FWM, KE]

Experiences shared by fieldworkers revealed that the issues were usually quite complex and intertwined with cultural, structural and emotional sensitivities associated with the need to balance community and cultural expectation against the policies and guidelines of research institutions. Thus, fieldworkers were often expected to strike a delicate balance, sometimes



For peer review only - http://bmjopen.bmj.com/site/about/guidelines.xhtml

without knowledge of how acceptable their actions were. Despite these actions having the potential to directly affect the scientific and ethical standards of data that fieldworkers collect, structural support and referral systems were reportedly not available. This was well captured by one senior fieldworker who said.

> "*…. sometimes, you get that huruma [pity], you go into your pocket and give out something…if you have a human heart, sometimes there are some situations that are touching"* [SFW, SA].

During interviews and workshop discussions, there were no accounts of existing internal or external institutional mechanisms to support fieldworkers to specifically deal with these types of emotional challenges. Although effective support supervision and sharing these challenges during team meeting was identified as a potential avenue to identify strategies to deal with these challenges, the approaches used to supervise fieldworkers were noted to be significantly different across institutions, and not centrally coordinated within institutions, making it difficult to reflect on their effectiveness. Workshop participants therefore generally felt there was a gap in fieldworkers' support structures that should be addressed. These challenges were seen as occurring across all the institutions involved in the study.

**5.3 Ways of strengthening institutional support**

Based on the outputs of discussions described so far, two core institutional fieldworker support strategies were raised, discussed and agreed upon over the course of the workshop. The first concerned adapting the *content of skill building initiatives* to support fieldworkers to manage their interactions with community members and household members in ways that could support good scientific and ethical practice, seen from multiple perspectives. The second strategic support mechanism addressed the wider question of how professional support within institutions could support fieldworkers more *structurally* towards these aims, including aspects of performance management that commonly exist for other groups of research staff. Workshop participants agreed that 'performance management' in this respect would include aspects of recruitment, training, supervision, support, assessment and career or professional development. Specifically, delegates emphasized the need to strengthen fieldworkers' interpersonal skills training to ensure they are well equipped to deal with cross-cutting issues around communication, community engagement, building trust and respect, which are fundamental in consenting and quality data collection. Fieldworkers themselves believed that more support would improve their skills, and accordingly the quality of the data they collect. The need to develop a harmonized training curriculum, with core training modules across Africa was identified.



For peer review only - http://bmjopen.bmj.com/site/about/guidelines.xhtml

In this study, we noted a mismatch related to lack of appropriate training for fieldworkers; fieldworkers' limited level of education and the complexity of the tasks they are expected to undertake, including but not limited to translating study information to study participants, understanding institutional and international ethical guidelines and applying these when interacting with study participants. These findings support the need for research institutions in Africa to invest in training and building the capacity of fieldworkers, in order to enhance the standards of the work fieldworkers undertake and provide optimum support for research.

### 5.4 Agreement on recommendations

Given the issues discussed in the preceding paragraphs around institutional fieldworkers' support structures, participants at the workshop agreed there was an urgent need to introduce more standardized and comprehensive approaches, focused on important areas that affect fieldworkers' performance and practices, including career progression, training/capacity building and management of emotional and ethical challenges. It was felt that strategies could build on the positive experiences of research institutions that had already begun to institutionalize such approaches. Participants recommended a pragmatic approach, aimed at empowering and building the knowledge and skills of fieldworkers, including processes to support continuous learning through the regular reflection of the practical and ethical issues they face in everyday experience. In this way, an important element of training and support supervision was described as enhancing skills to recognise and respond to these ethical issues. The specific recommendations made are described and discussed in the following paragraphs.

#### *5.4.1 Creating space for experience sharing and problem-solving*

One challenge identified during the survey was lack of psychological support after experiencing difficult and sometimes traumatic experiences, such as some interviewees breaking down during verbal-autopsy interviews, losing a study participant when close relationships had been built and even being physically attacked by emotional/aggrieved participants during interviews. Long working days with long distances to cover on foot, risks of injury through dog or snake bites, and physical assault in less secure settings were also said to be emotionally overburdening to fieldworkers. Documented evidence shows that stressful and traumatic experiences can alter the way people view themselves, the world around them, and alter how they process information and the way they behave and respond to the environment around them(29). A strategy described in the literature to address this challenge is the use of regular de-brief sessions, where fieldworkers are given opportunities to share experiences and consider the best approach to address challenges. During the workshop, participants recommended that field managers should create such opportunities for fieldworkers to talk about distressing or worrying issues encountered at work, through forms of feedback and



For peer review only - http://bmjopen.bmj.com/site/about/guidelines.xhtml

reflection. This approach also requires a support supervision mechanism that is sensitive to the complex nature of the experiences fieldworkers face. This supports True's (2011) arguments that having access to colleagues to discuss issues and having effective support supervision acted as a buffering factor for research front-line staff who did not engage in ethical misconduct, despite facing the same pressures to meet recruitment goals and other stressors of conducting community-based research (30). Combining different ways of knowing and learning will enable fieldworkers to standardize the way they handle practical and ethical issues they encounter, even where these present as dilemmas with limited information on the best course of action. At the same time, in some cases, more traditional approaches to managing emotional distress, such as staff counselling, might also be needed.

### *5.4.2 Increasing attention on soft skills for fieldworkers*

As reported by both fieldworkers and field managers, advancement has been made in the area of technical training for research staff, including fieldworkers. Good Clinical Practice, Health and Safety, Good Clinical Laboratory Practice (GCLP) training have become a standard approach for clinical study site preparation. While these trainings enable studies to adhere to international GCP/GCLP standards, they rarely equip fieldworkers with the necessary skills to enable them to deal with practical local challenges they face on the ground. There is, therefore, a need for fieldworkers to undergo more generic interpersonal skills training, such as communication skills, basic psychology and counselling and community engagement technics to enable them to appreciate the practical local situations and develop skills to deal with the ground challenges.

### *5.4.3 Having career development guidelines that are responsive to fieldworkers*

In a previous commentary by Kombe et al (2015), that reported the views of field managers who attended a consultative workshop as part of this study, important recommendations made by the managers were presented, including the need to i) increase institutional recognition of fieldworker's roles and the need for systematic and comprehensive capacity building to ensure adequate resources are allocated and that capacity building activities are well described in research proposals and grant applications; ii) develop common areas of a core curriculum for fieldworkers capacity building to enhance quality of training processes and build their knowledge in basic biology, research approaches and methods, research ethics and research regulatory frameworks as well as data collection and documentation; respectful communication, and being aware of and managing ethical challenges and issues in practice; and iii) increase emphasis on fieldworkers' career development including developing regionally



For peer review only - http://bmjopen.bmj.com/site/about/guidelines.xhtml

accredited training to support fieldworkers' professional development to increase individual motivation and enhance capacity for employment across different research organizations (12). These recommendations should be considered in order to address the challenges presented in this paper and enhance the overall quality and integrity of research in Africa. Similar strategies have been proposed by True et al, to address challenges faced by community members employed as research staff in the USA (2)

## 6. DISCUSSION

The important role fieldworkers play in health research can be enhanced if fieldworkers are given adequate support. The challenges they face require a combination of knowledge, skills, attitude change and ethical values that can be enhanced through structured institutional support and instilling a sense of professional responsibility and moral values to fieldworkers; through ongoing support supervision, soft skills training and creating space for consultative discussions and experience sharing.

In this study, fieldworkers were reported to provide important support for research. From the institutions that participated in the study, fieldworkers were employed to undertake diverse tasks and were given a range of different titles. All participants that were interviewed acknowledged the fact that the tasks undertaken by fieldworkers were critical in supporting health research. On the other hand, Fieldworkers reported experiencing a myriad of challenges, a fact that was supported by the fieldworker managers interviewed, and strongly deliberated upon during the workshop, during the second phase of this study.

Despite the above perceived importance of the tasks fieldworkers undertake in supporting health research, there were no centrally and institutionally coordinated systems or structures that were reported to have been put in place to provide the much-needed support for fieldworkers in most of the institutions involved in the study. Fieldworkers coordination and support were largely left in the hands of study specific field managers and supervisors, who often focused on the outputs of the specific studies they coordinated, with much less focus on generic support and supervision systems. Institutional systems to support fieldworkers to undertake their tasks effectively were reported to be limited or often unavailable. Furthermore, in spite of the myriad of challenges fieldworkers reported to experience in their day to day activities, which were confirmed by their field managers during the telephone interviews and the consultative workshop, no institutional systems were reported to be in place to support fieldworkers in addressing such challenges. Fieldworkers were therefore, essentially expected to use their discretion, cultural competence and moral reasoning to navigate through the many practical and ethical challenges they faced. Given the limited support, education and ethics



For peer review only - http://bmjopen.bmj.com/site/about/guidelines.xhtml

For peer review only

knowledge and competence fieldworkers possessed, this situation, points to serious implications on the quality of the data fieldworkers collect, their ethical practice and the overall integrity of the health research enterprise fieldworkers are expected to support.

This study interviewed participants via telephone and skype. Although this could not have necessarily affected the quality of the data collected, important qualitative interview dynamics, which can provide important insights on the data analysis process could not be identified and considered. However, the triangulation of different study approaches, including the consultative workshop with field managers, provided an important approach for validating the data collected and strengthening the study design.

The results from this study underscores the need to establish and strengthen institutional support for health research fieldworkers. Although a lot of studies have been done to understand the moral and ethical challenges and dilemmas faced by fieldworkers, very little has been done to explore which fieldworkers' institutional support systems exist and what implications such systems have on fieldworkers' scientific and ethical practices. There is therefore a need for further research, in order to develop a deeper understanding around this area. This study has highlighted important gaps related to the management and coordination of fieldworkers, that require further assessment and strategic consideration. Furthermore, the strategic solutions and recommendations identified in this study call for the need for health research institutions in Africa and funders of health research to strongly consider developing institutional systems and structures to facilitate centralized coordination and management of fieldworkers by health research institutions in Africa. As stated by Alexander and Richman, strengthening the capacity of fieldworkers has the potential to increase the protection of research participants, enhance research integrity and promote the collection of valid data (31). Efforts to empower fieldworkers to make them better able to deal with these challenges should therefore continue.

This was an exploratory study hence the results are not generalizable. However, they do offer important insights on fieldworkers and field managers' views and experiences of fieldworkers' performance managing practices for institutions that come from similar settings as those that participated in this study. Although some of the proposed approaches such as interpersonal skills training, centralized coordination of fieldworkers training, fieldworkers' scheme of service and regular discussions to address fieldwork challenges have been ongoing in institutions such as the KWRTP, in Kenya, lack of documented evidence makes it difficult to say how effective and sustainable such approaches are. More research, therefore, needs to be done to explore the feasibility of the proposed strategies and generalizability of these study findings within a wider context in Africa. Finally, the proposed solutions may not solve all the



For peer review only - http://bmjopen.bmj.com/site/about/guidelines.xhtml

challenges fieldworkers face, but they are certainly an important step towards improving the scientific and ethical standards of health research output from the African continent. The value of this study is not that every research centre in Africa can identify with every issue raised here, this would be difficult due to the variety of research centres in the continent, rather, we hope that by raising the issues highlighted here, even those centre that are not involved in the study will be provoked to examine their fieldworkers support system and see if any issues raised here apply to their setting. Furthermore, empirical studies should be done to identify which approaches are most effective in enabling research fieldworkers to deal with the myriad of challenges they face. Such research will go a long way in improving the scientific and ethical standards research, not only in Africa but internationally, given the critical global role of Africa in research.

## 7. DATA AVAILABILITY STATEMENT

Data are available in the form of transcripts that are currently archived in password protected computers. All the transcripts are anonymized, to avoid identification of the participants who were interviewed. Permission was obtained to use the data for purposes of this study only. No permission was granted to use the data for future research.



For peer review only - http://bmjopen.bmj.com/site/about/guidelines.xhtml

## 8. REFERENCES


1. Kamuya DM, Theobald SJ, Munywoki PK, Koech D, Geissler WP, Molyneux SC. Evolving friendships and shifting ethical dilemmas: fieldworkers' experiences in a short term community based study in Kenya. Dev World Bioeth. 2013;13(1):1-9.
2. True G, Alexander LB, Fisher CB. Supporting the role of community members employed as research staff: Perspectives of community researchers working in addiction research. Social Science & Medicine. 2017;187:67-75.
3. Kamuya DM, Theobald SJ, Marsh V, Parker M, Geissler WP, Molyneux SC. "The one who chases you away does not tell you go": silent refusals and complex power relations in research consent processes in Coastal Kenya. PloS one. 2015;10(5):e0126671.
4. Chantler T, Otewa F, Onyango P, Okoth B, Odhiambo F, Parker M, et al. Ethical Challenges that Arise at the Community Interface of Health R esearch: Village R eporters' Experiences in Western K enya. Developing world bioethics. 2013;13(1):30-7.
5. Kingori P. Experiencing everyday ethics in context: Frontline data collectors perspectives and practices of bioethics. Social science & medicine. 2013;98:361-70.
6. Madiega PA, Jones G, Prince RJ, Geissler PW. 'She's my sister-in-law, my visitor, my friend'–challenges of staff identity in home follow-up in an HIV trial in Western Kenya. Developing world bioethics. 2013;13(1):21-9.
7. Marshall PA, Marshall PL. Ethical challenges in study design and informed consent for health research in resource-poor settings: World Health Organization; 2007.
8. Richman KA, Alexander LB, True G. Proximity, ethical dilemmas, and community research workers. AJOB Primary Research. 2012;3(4):19-29.
9. Lansang MA, Dennis R. Building capacity in health research in the developing world. Bulletin of the World Health Organization. 2004;82(10):764-70.
10. Glickman SW, McHutchison JG, Peterson ED, Cairns CB, Harrington RA, Califf RM, et al. Ethical and scientific implications of the globalization of clinical research. New England Journal of Medicine. 2009;360(8):816-23.
11. Participants of an International Workshop in Kenya on the Role of Frontline Staff in Biomedical Research J, Kombe F. Enhancing quality and integrity in biomedical research in Africa: an international call for greater focus, investment and standardisation in capacity strengthening for frontline staff. BMC Med Ethics. 2015;16(1):77.
12. Kombe F. Enhancing quality and integrity in biomedical research in Africa: an international call for greater focus, investment and standardisation in capacity strengthening for frontline staff. BMC medical ethics. 2015;16(1):77.
13. Srivastava A, Thomson SB. Framework analysis: a qualitative methodology for applied policy research. 2009.
14. Molyneux S, Kamuya D, Madiega PA, Chantler T, Angwenyi V, Geissler PW. Field workers at the interface. Developing world bioethics. 2013;13(1):ii.
15. Kingori P. Experiencing everyday ethics in context: frontline data collectors perspectives and practices of bioethics. Social science & medicine. 2013;98:361-70.
16. Molyneux S, Kamuya D, Madiega PA, Chantler T, Angwenyi V, Geissler PW. Field workers at the interface. Dev World Bioeth. 2013;13(1):ii-iv.
17. Chantler T, Otewa F, Onyango P, Okoth B, Odhiambo F, Parker M, et al. Ethical challenges that arise at the community interface of health research: village reporters' experiences in Western Kenya. Dev World Bioeth. 2013;13(1):30-7.
18. Angwenyi V, Kamuya D, Mwachiro D, Marsh V, Njuguna P, Molyneux S. Working with Community Health Workers as 'volunteers' in a vaccine trial: practical and ethical experiences and implications. Dev World Bioeth. 2013;13(1):38-47.
19. Kamuya DM, Theobald SJ, Munywoki PK, Koech D, Geissler WP, Molyneux SC. Evolving Friendships and Shifting Ethical Dilemmas: Fieldworkers' Experiences in a Short Term Community Based Study in K enya. Developing world bioethics. 2013;13(1):1-9.




For peer review only - http://bmjopen.bmj.com/site/about/guidelines.xhtml

20. Wax ML. On fieldworkers and those exposed to fieldwork: Federal regulations and moral issues. Human Organization. 1977;36(3):321.
21. Zinn MB. Field research in minority communities: Ethical, methodological and political observations by an insider. Social problems. 1979;27(2):209-19.
22. Kombe F, Anunobi EN, Tshifugula NP, Wassenaar D, Njadingwe D, Mwalukore S, et al. Promoting Research Integrity in A frica: An African Voice of Concern on Research Misconduct and the Way Forward. Developing world bioethics. 2014;14(3):158-66.
23. Molyneux S, Kamuya D, Marsh V. Community members employed on research projects face crucial, often under-recognized, ethical dilemmas. The American Journal of Bioethics. 2010;10(3):24-6.
24. Mosavel M, Ahmed R, Daniels D, Simon C. Community researchers conducting health disparities research: Ethical and other insights from fieldwork journaling. Social science & medicine. 2011;73(1):145-52.
25. Simon C, Mosavel M. Community members as recruiters of human subjects: Ethical considerations. The American Journal of Bioethics. 2010;10(3):3-11.
26. Davis AM, Hull SC, Grady C, Wilfond BS, Henderson GE. The invisible hand in clinical research: The study coordinator's critical role in human subjects protection. The Journal of Law, Medicine & Ethics. 2002;30(3):411-9.
27. Zahr CA, Pinchuk M. Verbal autopsy standards: ascertaining and attributing cause of death: World Health Organization; 2007.
28. Fottrell E, Byass P. Verbal autopsy: methods in transition. Epidemiologic reviews. 2010;32(1):38-55.
29. Steele W. Trauma's Impact on Learning. Trauma and Loss: Research and Interventions. 2002.
30. True G, Alexander LB, Richman KA. Misbehaviors of front-line research personnel and the integrity of community-based research. J Empir Res Hum Res Ethics. 2011;6(2):3-12.
31. Alexander LB, Richman KA. Ethical Dilemmas in Evaluations Using Indigenous Research Workers. American Journal of Evaluation. 2008;29(1):73-85.

For peer review only



For peer review only - http://bmjopen.bmj.com/site/about/guidelines.xhtml

## Contributor and guarantor information

All authors were involved in conceptualizing the idea and contributed in writing the manuscript. Mr Francis Kombe (MPH), a male senior community facilitator with a wealth of experience in health research ethics, community engagement and communication skills, who worked as a fieldworker himself, before taking a managerial role at KWTRP, contributed to the planning, conduct, and reporting of the work described in the article and is responsible for the overall content. Prof Vicki Marsh (PhD), a female senior social scientist at KWTRP and Oxford University and Ms Dorothy Ikamba (Msc), a female career development officer at KWTRP, and career counsellor, assisted with reviewing the transcripts and the development of themes and sub-themes during the analysis. Prof Sassy Molyneux (PhD), a female senior social scientist at KWTRP and Oxford University, Dr Dorcas Kamuya (PhD), a female social scientist at KWTRP and Wellcome Trust Society & Ethics Fellow, and Dr Samson Kinyanju, a male immunologist and head of training at the KWTRP, contributed in designing the initial research ideas, reviewed the data collected and were involved in drafting the initial manuscript and edited subsequent versions. All authors reviewed and approved the final manuscript for publication. Francis Kombe accepts full responsibility for the work and the conduct of the study. He had full access to the data, and controlled the decision to publish. Francis Kombe attests that all listed authors meet authorship criteria and that no others meeting the criteria have been omitted.

## Copyright/license for publication

Francis Kombe has the right to grant on behalf of all authors and does grant on behalf of all authors, a worldwide license to the Publishers and its licensees in perpetuity, in all forms, formats and media (whether known now or created in the future), to i) publish, reproduce, distribute, display and store the Contribution, ii) translate the Contribution into other languages, create adaptations, reprints, include within collections and create summaries, extracts and/or, abstracts of the Contribution, iii) create any other derivative work(s) based on the Contribution, iv) to exploit all subsidiary rights in the Contribution, v) the inclusion of electronic links from the Contribution to third party material where-ever it may be located; and, vi) license any third party to do any or all of the above

For peer review only

## Transparency statement

Francis Kombe affirms that the manuscript is an honest, accurate, and transparent account of the study being reported; that no important aspects of the study have been omitted; and that any discrepancies from the study as originally planned have been explained.

## Competing interests

The views on this paper are the authors own viewpoints and do not represent the position of their organizations. The authors declare no conflict of interests.
The authors declared that they have no competing interests.

## Ethical approval

The paper is published with the permission of the Centre director, KEMRI. All the participant who participate in the telephone survey as well as those who attended the consultative workshop gave individual consent to participate in the study and the workshop, and permitted the publication of this manuscript. Permission to conduct this study was granted by a review conducted by the Centre for Geographic Medicine Research, Coast (CGMRC)- Centre Specific Committee and a final Ethical approval given by the Research and Scientific Review Unit (SERU) at the Kenya Medical Research Institute (KEMRI).

For peer review only



For peer review only - http://bmjopen.bmj.com/site/about/guidelines.xhtml

**DISCUSSION GUIDE FOR RESEARCH INSTITUTIONS WORKING WITH FIELDWORKERS (FWs) IN SSA**

**TITLE: STRENGTHENING TRAINING OF FRONT LINE (FWs) STAFF AS A MEANS TO ENHANCE COMMUNITY ENGAGEMENT**

**INFORMATION SHEET FOR MANAGERS AND SENIOR FRONTLINE STAFF**

## What is this survey all about?

This survey will be conducted through telephone/ teleconference discussion. One front line staff manager (people who have a direct role of providing support supervision to frontline staff) and one senior frontline staff member from a total of 20 health research institutions in Sub-Saharan Africa will be invited to participate in the discussion.

The aim of the discussion is to identify practical and ethical challenges experienced by frontline staff in their day to day activities and the available support supervision, performance management and Continuing Professional Development (CPD) practices within these institutions. Later, representatives from a few of the 20 institutions will be invited to a consultative workshop to follow up and discuss these issues in detail. The report generated from the survey and workshop will be shared with all institutes for their own use. In the long term, the report could be used to inform further discussions on the potential of developing a generic curriculum for frontline staff training.

## What is expected of you if you take part in this discussion?

If you agree to take part in this discussion, we will request you to select a date and time when you would prefer the moderator to call and have the discussion with you. We will then call and ask a few questions with regards to your perceptions of the roles of frontline staff and the institutional support supervision, monitoring and evaluation and general CPD practices. These questions are shown on the attached discussion guide for your attention. The discussion is expected to take a maximum of 30 minutes.

Participation in this interview is voluntary. While it is important to answer all the questions, feel free to skip any question that you do not feel comfortable to answer and the moderator will continue asking the next question. You can also decide to terminate the discussion at any time if you decide not to continue with the discussion for any reason.

## Is there any individual benefit for taking part in this discussion?

There is no individual benefit in taking part in this discussion. However, by sharing your experiences in working with frontline staff, you will assist in contributing to knowledge that could improve the way frontline staff deal with their practical and ethical challenges as well as their support supervision, performance and general CPD management practices in future.

## If you have any question

If you have any question about this survey, please contact any of the following people through the email addresses provided below

| | | |
|---|---|---|
| Francis Kombe<br>KWTRP<br>P.O BOX 230, KILIFI<br>Fkombe@kemri-wellcome.org | Sam Kinyanjui<br>KWTRP<br>P.O BOX 230, KILIFI<br>Skinyajui@kemri-wellcome.org | Dorcas Kamuya<br>KWTRP/ Ethox-Nuffield Department of Population Science, Oxford University<br>P.O BOX 230, KILIFI<br>Dkamuya@kemri-wellcome.org |

For peer review only

**DISCUSSION GUIDE FOR RESEARCH INSTITUTIONS WORKING WITH FIELDWORKERS (FWs) IN SSA**

**TITLE: STRENGTHENING TRAINING OF FRONT LINE (FWs) STAFF AS A MEANS TO ENHANCE COMMUNITY ENGAGEMENT**

## SECTION A: Institutional Background

1. Name of organization ___________________________________________
2. Vision ___________________________________________
3. Mission ___________________________________________
4. Motto ___________________________________
5. Year organization was established [__][__][__] [__]
6. Source of main funding for the organization ___________________________________
7. Other funding agencies and collaborators ___________________________________
8. Types of research conducted at the organization: _________________________________

## SECTION B: Frontline staff Managers' Perspective[1]

*This section explores managers' perceptions of the roles of and practical and ethical challenges faced by interface staff when discharging their duties and the institutional support supervision, performance management and Continuing Professional Development (CPD) practices*

1. What names are given to your frontline staff whose primary role involves recruiting and consenting research participants into studies?

2. What key roles/activities do they undertake in your organization?

3. In your institution, how many staff does it have (in total) and how many of these are employed as frontline staff?

4. What is the range of qualifications for frontline staff and what is their minimum level of education/qualification?

5. What is the nature of their employment e.g. are they in contract? What is the average contract period? Are the contracts renewable and what is considered during renewal? Any systems that ensure retention of frontline staff in the institutions?

6. What systems are in place to coordinate the way frontline staff are employed and retained across different studies in the institutions? What is the role of PI (if any) in retaining frontline staff?

7. What formal and informal processes are in place to induct new frontline staff when they first arrive, and to train them for their roles?

---

[1] Manager is the person whose primary responsibility involves working directly with front line staff

For peer review only

**DISCUSSION GUIDE FOR RESEARCH INSTITUTIONS WORKING WITH FIELDWORKERS (FWs) IN SSA**

**TITLE: STRENGTHENING TRAINING OF FRONT LINE (FWs) STAFF AS A MEANS TO ENHANCE COMMUNITY ENGAGEMENT**

---

*8.* How is the training for interface staff organized? Are there any refresher trainings/skills improvement activities? What are the *training approach eg frequency and objectives of the training,*

*9.* How is the frontline staff training managed? Describe funding, design, whether centralized or decentralized, and monitoring and evaluation of the training

*10.* Describe any practices, experiences and studies (if any) that have informed the current training eg feedback mechanisms; availability of a community engagement strategy, community Knowledge Attitude and Perception (KAP) surveys etc

*11.* How do you ensure skills learned are assimilated into the frontline's day to day activities? Are there any support supervision mechanisms, performance appraisals etc ? How are these implemented?

*12.* Describe any career progression scheme used by your organization for these staff groups

*13.* What challenges do you experience in working with and managing frontline staff?

## SECTION C: Frontline staffs' Experience-Staff perspective

*This section explores frontline staff's practical and ethical challenges faced as they discharge their duties and their perceptions of the institutional support in training and CPD*

1. What are roles and responsibilities of frontline staff?

2. What challenges do frontline staff face when interacting with community members? *Practical and ethical challenges/dilemmas faced, e.g. issues of confidentiality?*

3. What type of in-house and external training is given to frontline staff in your institution?
   - ***Which of these do you perceive to be most appreciated and for what reason?***

4. What are the perceived institutional challenges in supporting and capacity building of frontline staff?
   - ***How do frontline members of staff address the above challenges?***

## SECTION D: Planned Workshop

At the end of this survey, we hope to invite a selected number of the institutions to a 2 day workshop. Kindly indicate if you would like to be considered to attend this workshop.

For peer review only

# COREQ (COnsolidated criteria for REporting Qualitative research) Checklist

A checklist of items that should be included in reports of qualitative research. You must report the page number in your manuscript where you consider each of the items listed in this checklist. If you have not included this information, either revise your manuscript accordingly before submitting or note N/A.

| Topic | Item No. | Guide Questions/Description | Reported on Page No. |
|---|---|---|---|
| **Domain 1: Research team and reflexivity** | | | |
| *Personal characteristics* | | | |
| Interviewer/facilitator | 1 | Which author/s conducted the interview or focus group? | 22 |
| Credentials | 2 | What were the researcher's credentials? E.g. PhD, MD | 22 |
| Occupation | 3 | What was their occupation at the time of the study? | 22 |
| Gender | 4 | Was the researcher male or female? | 22 |
| Experience and training | 5 | What experience or training did the researcher have? | 22 |
| *Relationship with participants* | | | |
| Relationship established | 6 | Was a relationship established prior to study commencement? | 6 |
| Participant knowledge of the interviewer | 7 | What did the participants know about the researcher? e.g. personal goals, reasons for doing the research | 6 |
| Interviewer characteristics | 8 | What characteristics were reported about the inter viewer/facilitator? e.g. Bias, assumptions, reasons and interests in the research topic | 3, 10, 22 |
| **Domain 2: Study design** | | | |
| *Theoretical framework* | | | |
| Methodological orientation and Theory | 9 | What methodological orientation was stated to underpin the study? e.g. grounded theory, discourse analysis, ethnography, phenomenology, content analysis | 5 |
| *Participant selection* | | | |
| Sampling | 10 | How were participants selected? e.g. purposive, convenience, consecutive, snowball | 5 |
| Method of approach | 11 | How were participants approached? e.g. face-to-face, telephone, mail, email | 5 |
| Sample size | 12 | How many participants were in the study? | 5 |
| Non-participation | 13 | How many people refused to participate or dropped out? Reasons? | 5 |
| *Setting* | | | |
| Setting of data collection | 14 | Where was the data collected? e.g. home, clinic, workplace | 8 |
| Presence of non-participants | 15 | Was anyone else present besides the participants and researchers? | 5 |
| Description of sample | 16 | What are the important characteristics of the sample? e.g. demographic data, date | 6 |
| *Data collection* | | | |
| Interview guide | 17 | Were questions, prompts, guides provided by the authors? Was it pilot tested? | 8 |
| Repeat interviews | 18 | Were repeat inter views carried out? If yes, how many? | 8-10 |
| Audio/visual recording | 19 | Did the research use audio or visual recording to collect the data? | 8 |
| Field notes | 20 | Were field notes made during and/or after the inter view or focus group? | 8 |
| Duration | 21 | What was the duration of the inter views or focus group? | 8 |
| Data saturation | 22 | Was data saturation discussed? | |
| Transcripts returned | 23 | Were transcripts returned to participants for comment and/or | |

| Topic | Item No. | Guide Questions/Description | Reported on Page No. |
|---|---|---|---|
| | | correction? | |
| **Domain 3: analysis and findings** | | | |
| *Data analysis* | | | |
| Number of data coders | 24 | How many data coders coded the data? | 9 |
| Description of the coding tree | 25 | Did authors provide a description of the coding tree? | |
| Derivation of themes | 26 | Were themes identified in advance or derived from the data? | 9-10 |
| Software | 27 | What software, if applicable, was used to manage the data? | 9 |
| Participant checking | 28 | Did participants provide feedback on the findings? | 9 |
| *Reporting* | | | |
| Quotations presented | 29 | Were participant quotations presented to illustrate the themes/findings? Was each quotation identified? e.g. participant number | 10-16 |
| Data and findings consistent | 30 | Was there consistency between the data presented and the findings? | |
| Clarity of major themes | 31 | Were major themes clearly presented in the findings? | 10-16 |
| Clarity of minor themes | 32 | Is there a description of diverse cases or discussion of minor themes? | 10-16 |

Developed from: Tong A, Sainsbury P, Craig J. Consolidated criteria for reporting qualitative research (COREQ): a 32-item checklist for interviews and focus groups. *International Journal for Quality in Health Care*. 2007. Volume 19, Number 6: pp. 349 – 357

**Once you have completed this checklist, please save a copy and upload it as part of your submission. DO NOT include this checklist as part of the main manuscript document. It must be uploaded as a separate file.**

For peer review only

BMJ Open

# Enhancing Fieldworkers' Performance Management Support in Health Research: An exploratory study on the views of Field managers and Fieldworkers from major research centres in Africa

| | |
|---|---|
| Journal: | *BMJ Open* |
| Manuscript ID | bmjopen-2018-028453.R3 |
| Article Type: | Original research |
| Date Submitted by the Author: | 28-Oct-2019 |
| Complete List of Authors: | Kombe, Francis; Centre for Geographic Medicine Research Coast; African Research Integrity Network<br>Marsh, V; Centre for Geographic Medicine Research Coast; Centre for Tropical Medicine and Global Health, Nuffield Department of Medicine, Oxford University, UK<br>Molyneux, Sassy ; Centre for Geographic Medicine Research Coast; Centre for Tropical Medicine and Global Health, Nuffield Department of Medicine, Oxford University, UK<br>Kamuya, Dorcas; KEMRI Wellcome Trust Research Programme; Ethox Centre, Nuffield Department of Population Health, Oxford University, UK<br>Ikamba, Dorothy; KEMRI Wellcome Trust Research Programme<br>Kinyanjui, Samson; Centre for Geographic Medicine Research Coast; Centre for Tropical Medicine and Global Health, Nuffield Department of Medicine, Oxford University, UK |
| <b>Primary Subject Heading</b>: | Ethics |
| Secondary Subject Heading: | Ethics, Qualitative research |
| Keywords: | Fieldworkers, Africa, Capacity Building, Health Research, Research Ethics, Research Integrity |
| | |

SCHOLARONE™
Manuscripts

**Enhancing Fieldworkers' Performance Management Support in Health Research: An exploratory study on the *views of Field managers and Fieldworkers from major research centres in Africa***

Francis Kombe[1], Vicki Marsh[1,3], Sassy Molyneux[1,3], Dorcas Kamuya[1,2], Dorothy Ikamba[1], Kinyanjui Samson Muchina[1,3]

[1]KEMRI Wellcome Trust Research Programme, Kenya; [2]Ethox Centre, Nuffield Department of Population Health, Oxford University, UK; [3]Centre for Tropical Medicine and Global Health, Nuffield Department of Medicine, Oxford University, UK.

**Corresponding author**: Francis Kombe, KEMRI Wellcome Trust Research Programme, PO Box 230, Kilifi 80108, Kenya. Mobile +254 722459045; Email-Kajoleh@gmail.com

**Acknowledgements**: We acknowledge and greatly appreciate the support of all research institutions' Directors who allowed their field managers and field workers to take part in this project, and all the field managers and field workers who participated. We are extremely indebted to Prof Mike Parker, the Director of the Wellcome Centre for Ethics and Humanities and Director of Ethox Centre at the University of Oxford for his support and guidance during the project; and all the senior field managers and delegates who attended the consultative workshop. The manuscript is published with the support of the Director, KEMRI.

**Funding**:

This project was funded by Global Health Bioethics Network-UK (Wellcome Trust grant no. OXF-GHB02) and supplemented by KWTRP Capacity Building Strategic Award



For peer review only - http://bmjopen.bmj.com/site/about/guidelines.xhtml

(Wellcome Trust grant no 084538). The research and capacity building activities of the Global Health Bioethics Network are supported by a Wellcome Trust Strategic Award (096527)

**Keywords:** Africa, Fieldworkers, Research Integrity, Research Ethics, Capacity Building.

**Word Count: 7497**

For peer review only

**ABSTRACT**

**Introduction**

Fieldworkers are part of the system that promotes scientific and ethical standards in research, through data collection, consenting and supporting research, due to their insider cultural knowledge and fluency in local languages. The credibility and integrity of health research, therefore, rely on how fieldworkers adhere to institutional and research procedures and guidelines.

**Study objectives**

This study mapped out existing practices in training, support and performance management of fieldworkers in Africa, described fieldworkers and their managers' experiences, and lessons learned. Consultative process, involving field managers from 15 international health research institutions, was used to identify appropriate ways of addressing the challenges fieldworkers face.

**Methods**

**Phase one-** We conducted Thirty-two telephone interviews with Twenty field managers and Twelve senior fieldworkers from Eighteen major research Centres in Africa, MRC-UK and the INDEPTH Network Secretariat.

**Phase two-** We held a two-and-a-half days workshop involving Twenty-five delegates including Eighteen field managers from the institutions that were involved in Phase One; and Seven additional stakeholders from the KWTRP. An earlier report from phase one was published in BMC Med Ethics in 2015. Data transcribed from the interviews and workshop proceedings were analyzed thematically using Nvivo 10 software.

**Results**

Most institutions employed fieldworkers, usually with Twelve years of formal education and residing within the geographic areas of research, to support studies. Although their roles were common, there were marked differences in the type of training, professional development schemes and fieldworkers support. Fieldworkers faced various challenges, with the potential to affect their ethical and scientific practices.

**Discussion**

Fieldworkers undertake vital tasks that promote data quality and ethical practice in research. There is a need for research institutions to develop a structured support system, provide



For peer review only - http://bmjopen.bmj.com/site/about/guidelines.xhtml

fieldworkers with interpersonal-skills training and provide space for discussion, reflection and experience-sharing to help fieldworkers tackle the practical and ethical challenges they face.

**Strengths and limitations of this study**

**Strengths**

- Findings of this study add to a growing body of literature on how to enhance the quality and integrity of data collected with the support of fieldworkers; an important, but largely under supported and understudied cadre of staff, in international research settings in Africa. The fact that participants of this study were drawn from West, South and East Africa makes the findings of this study rich with an extended spectrum of experiences adduced, which provide deeper insights to the issues that were discussed.
- The use of consultative approaches, that allowed issues raised during telephone interviews to be followed up and discussed further in a workshop, provided a unique opportunity to cross-check data from fieldworkers with their managers, reflect on and unpack important issues, with a view to identifying relevant and applicable strategies for addressing challenges faced.

**Limitations**

- All interviews were done by phone. As such, important interview dynamics may have been missed. Face to face interviews would have been able to pick important non-verbal cues, which was not possible through telephone interviews. In addition, because our interviews focused on fieldworkers' challenges and potential opportunities, this could also have raised the fieldworkers' expectation to receive direct support to address the challenges they faced and could have contributed to overreporting of the challenges they experienced. Since the fieldworkers' managers reported similar challenges, we think this may provide some support for the validity of the fieldworkers data.
- The interviewer was a senior community facilitator at KWTRP and had worked as a fieldworker prior to being promoted to this position. It is possible that his personal experiences influenced his interpretation of the data. However, this was addressed through continuous discussions with two of the co-authors (VM and DK), including discussing the emerging themes from the data and reflecting on his positionality in the research process. This enabled him to reflect on his potential influence, in the data collection process, analysis and interpretation.
- Interviewees were nominated by Centre Directors, who were the primary contact people. Although individual consent was obtained from those nominated by the Directors prior to the interviews, it is possible that interviewees could have reported what they believed



For peer review only - http://bmjopen.bmj.com/site/about/guidelines.xhtml

would be acceptable to their directors. During the interviews, we assured our participants about the confidentiality of the data they would share, which we think enabled them to share information sincerely and transparently. However, there is a potential that the power differentials between the participants (field managers and fieldworkers), and the Centre Directors could have influenced their views and perceptions of the discussions held. Despite fieldworkers being hierarchically lower compared to field managers, and having different powers and authority within the research system, we could not identify any strong divergence in the views they shared. Furthermore, the institutions that participated in this study were selected on the basis of existing relationship with the INDEPTH Network and the KWTRP. It is possible that other institutions outside these networks had different experiences from those that participated in this study. We appreciate that this is an exploratory study. We believe the institutions involved shared a wide range of experiences and that findings from this study offer important insights for further research, especially for international health research institutions that employ fieldworkers.

For peer review only



For peer review only - http://bmjopen.bmj.com/site/about/guidelines.xhtml

# 1. BACKGROUND

High-quality data is a critical output of all science worldwide. Thus, research institutions put significant emphasis on generating data that are of the highest scientific and ethical standards. In many research programmes in low and middle-income countries, much of the data is collected by frontline staff referred to as “fieldworkers”. This cadre of staff is also often responsible for recruiting potential research participants, administering consent forms and following up participants during studies (1). As such, beyond ensuring data integrity, fieldworkers play a key role in ensuring the ethical implementation of research protocols. The role of fieldworkers is particularly critical in international collaborative programmes with researchers drawn from outside local communities. In such settings, fieldworkers, who are normally from the local communities, can serve as the face of research programmes with potential to significantly influence communities’ trust in research.

In carrying out these complex roles, fieldworkers face practical and ethical dilemmas that require the balancing of institutional roles and guidelines against community expectations and fieldworkers’ personal interests (2). Hence, fieldworkers continuously make independent (often unconscious) decisions on how to apply ‘ethical principles’ on the ground. For example fieldworkers reported challenges such as dealing with ‘silent refusals’ (3) (where participants avoid key study procedures but do not openly refuse), and with many requests for health and other social- economic support that are not included in the study protocol (4-8).

## 1.1 Capacity Building and Investiment In Research

Over the years there has been an increasing interest and investment in developing research capacity in Africa and other low and middle income regions through long-term initiatives such as the WHO- Tropical Diseases Research Programme (TDR) (9), the European and Developing Countries Clinical Trials Initiative Partnership (EDCTP) and more recently, The Wellcome Trust-DFID funded “Developing Excellence in Leadership, Training and Science “scheme (DELTAS) Africa. Efforts to strengthen research capacity are likely to continue, as the trend towards greater cross-border data sharing, globalization of clinical research and comparability of data quality at the global level continues (10).

Given that fieldworkers play such a vital role in health research, it would be expected that research programmes, in addition to training scientists, would also focus on building fieldworkers’ capacity to carry out their duties in the most effective manner. However, fieldworkers’ capacity and skill sets have received relatively little attention. The mechanisms and processes by which research institutions in Africa support fieldworkers in undertaking their duties remain unclear. Apart from the Good Clinical Practice guidelines, which focus primarily on clinical trials, no other guidelines are available that provide a framework for the content and



For peer review only - http://bmjopen.bmj.com/site/about/guidelines.xhtml

approach for supporting the Continuing Professional Development and career growth of fieldworkers.

Understanding the challenges that fieldworkers face and the type of systems, and level of support research institutions in Africa provide, can enable us to identify opportunities for strengthening fieldworkers' scientific and ethical practices.

In this paper, we draw on our findings from a two-phase qualitative exploratory study on the perceptions, experiences and views of field managers and fieldworkers on the structural and performance management support extended to health research fieldworkers with the aim of informing institutional policies that promote research integrity and ethical practices for this critical cadre of staff (11).

## 2. STUDY OBJECTIVES

This study mapped out existing practices in relation to training, support and performance management of fieldworkers in Africa, fieldworkers' and their managers' experiences, and lessons learned. In addition, field managers from international health research institutions were involved in a consultative workshop to identify appropriate ways of addressing the challenges faced by fieldworkers working in research Centres in Africa.

## 3. METHODS

The study was planned over two phases, and focused on understanding structural and performance management practices and their implications within institutions. In Phase One, which was implemented in February 2014, we conducted semi-structured telephone interviews with Thirty-two fieldworker managers and experienced fieldworkers across Eighteen health research institutions in Fourteen countries in Africa and the United Kingdom. In Phase two, which was implemented in July 2014, we held a two and a half days workshop involving Twenty-five delegates. Eighteen of these delegates were from the institutions that were involved in phase I (i.e. one per institution). The workshop also included the Director of Training, three senior social scientists with long term experience in managing fieldworkers (also Co-PIs of the project), the Head of community engagement and two senior fieldworkers' supervisor from the KWTRP (12). Fieldworkers were not involved in the second phase.

This paper focuses on the results from Phase 1 (telephone interviews). Issues that were identified during Phase One were further discussed in more detail in the consultative workshop in order to build consensus, and identify common recommendations and strategies for addressing the reported challenges. Thus, there are certain sections of the paper that inevitably refer to views shared during the workshop. Results of the consultative workshop have already been published (12).



For peer review only - http://bmjopen.bmj.com/site/about/guidelines.xhtml

**3.1 Patient and public involvement**

Patients and the public were not involved in the design and conception of this study

**3.2 Study site and selection of research institutions and interview participants.**

This study was led by a team based at the Kenya Medical Research Institute (KEMRI) Wellcome Trust Research Programme (KWTRP) in Kenya. The KWTRP is a long-standing international health research institution with a wide range of existing scientific and capacity building collaborations with other research institutes in sub-Saharan Africa (see http://kemri-wellcome.org/). During the first phase, our sampling strategy for research institutions, and relevant individuals within these, focused on African institutions that manage Health and Demographic Surveillance Systems, given their employment of significant numbers of fieldworkers. We identified these institutions from our existing scientific and capacity building linkages, including the INDEPTH Network (http://www.indepth-network.org), an international research network for health and demographic surveillance sites in Africa which encompasses all research centers in sub-Saharan Africa that have Health Demographic Surveillance Systems. From the original list of 34 institutions obtained from the KWTP training department and INDEPTH site, we sent an email to the Directors, with an information sheet about the study, to invite them to participate in the study. A total of eighteen African international research institutions (see table 1) and the MRC-UK and the INDEPTH Network Secretariat in Ghana responded positively. A total of Sixteen Directors did not reply to our email and could not therefore determine why they did not respond; thus the reasons for nonresponse are unknown. These were dropped after three reminders. The Eighteen African research institutions included a mix of institutions with varied years of operations that employed a diverse number of fieldworkers, engaged in a range of local and international collaborations and specialized in different research programmes/thematic areas. This mixture of characteristics was important in maximizing shared learning and experiences across institutions. In each institution, we wrote to the Director and sought permission to interview one senior fieldworker and one field manager. In the letter, we particularly underscored the need to select a manager whose primary responsibility involved working directly with and managing fieldworkers, including offering support supervision, training and other forms of performance management.

3.2 **Data collection and analysis**



For peer review only - http://bmjopen.bmj.com/site/about/guidelines.xhtml

### 3.2.1 *Phase One: Telephone Interviews with managers and fieldworkers*

Out of the 34 Directors contacted, 18 sent names of two individuals (1 senior fieldworker and 1 field manager) to be interviewed. We then contacted the proposed individuals and sought their individual consent to participate in a semi-structured interview, using telephone or Voice Over Internet Protocol (VOIP) communication. Interviews were conducted using an interview guide (Appendix A), that was developed in consultation with members of the study team and pre-tested prior to being used. In total, the 18 institutions that participated in the study employed a total workforce of 7839 staff, with 2505 (32.0%) being fieldworkers. Table 1 shows all the institutions that participated in the study, marked in dark blue (those that attended the workshop) and light blue (those that did not attend the workshop), with their names anonymized.

Seven of the Research Institutions that participated in the interviews were from West Africa, four were from Southern Africa and Seven were from East Africa. In addition, individuals from one regional research coordination institution from Africa and one UK research funding organization were also interviewed. The Institutions conducted research in various fields including but not limited to Infectious tropical and bacterial diseases (n=9), Population Health and HIV studies (n=7), Social science and health systems research (n=9), Clinical Research/Drug and Vaccine trials (n=5) Epidemiology-Prevention and control of Malaria (n=9), Maternal and Newborn Health (n=6), Non-communicable diseases (n=3) fundamental sciences (n=1), human genetics (n=1), Molecular biology (n=1) and Clinical biology and bioinformatics (n=1). All the institutions received funding from external donors, mainly from the USA, United Kingdom (UK) and Canada. The number of staff employed per institution ranged from 45 to 1500 (mean 461), with the number of fieldworkers ranging from 30 to 630 (mean 147).

For peer review only



For peer review only - http://bmjopen.bmj.com/site/about/guidelines.xhtml

**Table 1: Main characteristics of Institutions Involved in the project**

| No | Name of Institution | Year Established | The main type of research | Number of Staff | Number of fieldworkers | % of FWs | Source of Funding | Description of training given to Fieldworkers | Description of career progressing system used |
|---|---|---|---|---|---|---|---|---|---|
| 1. | **Research Institution 1-WA.** | 1947 | Infectious tropical diseases | 1104 | 301 | 27.3 | MRC, UK | CORE training is given to all fieldworkers with three level, including basic, intermediary and advanced. Everybody who gets employed as a fieldworker must go through core training. | Successful completion of fieldworker straining is automatically awarded one salary grade promotion and allows fieldworkers to move through a career progression from A2 A3, B1 B2 B3, C1 C2 C3, D1 D2 D3 and E1 E2 E3. Some fieldworkers move up to C2 after the training because of the change in job description and responsibilities. |
| 2. | **Research Institution 2-SA.** | 1997 | Health and Population, HIV studies, | 800 | 52 | 5.4 | WT, NIH & EGPAF | A mass training is done for 5 weeks: 4 weeks course work and 1 week practical. The test is done to assess skills gained and certificate issued upon passing the exams. Those who pass are put on a waiting list and employed directly to studies when a vacancy arises. | A professional development process involving all staff is available. It is not specific to fieldworkers. |
| 3. | **Research Institution 3-WA.** | 1983 | Anthropology, Demography, Health Economics, Clinical trials | 153 | 40 | 26.1 | NIH and USAID, WHO & EGAD, | A centrally managed training coordinated by a social scientist who takes through the fieldworkers on how to approach the community is usually given | Fieldworkers are usually outsourced. No scheme of service is available |
| 4. | **Research Institution 4-EA.** | 1970 | Infectious diseases, prevention and control | 120 | 40 | 33.3 | NORAD, SIDA, EDCTP, WT, BMGF, WHO, TDR, GLOBVAC, Swiss TPH, | New staff are trained on the specific project and other training such as GCP, Data Management and Ethics. | There is no career ladder but if they improve their educational qualification, they get an opportunity to apply for other higher posts |
| 5. | **Research Institution 5-SA.** | 1996 | Malaria, HIV AIDS, Vaccine Trials, Pneumonia, Bacterial infection | 200 | 100 | 50.0 | AECID, MOH & EDPTP | All fieldworkers undergo project-related training, GCP, Data Management and Ethics upon being employed | None |
| 6. | **Research Institution 6-EA** | 1989 | Immunology, TB, Bacteremia, HIV, Social Science, Health Economics, | 750 | 300 | 40 | WT, IAVI, NIH | Fieldworkers training is centrally coordinated. All new fieldworkers undergo training in communication skills, research ethics, introduction to medical research, GCP and phlebotomy before being allowed to work in the field | A career progression framework for fieldworkers exists, which stipulate the entry point and career progression subject to attainment of job experience, in-house training as well as external training which could either be self or programme sponsored |
| 7. | **Research Institution 7-EA** | 1998 | Health system research, Maternal and Newborn Health & HIV | 700 | 135 | 19.3 | DFID, INDEPTH, MOH, IDRC, DFID, RF, BMGF, FP7, USAID & CDC-RBM- | Field enumerators who work in the DSS go through a refresher training every six months to share the challenges from the previous round and train them on new issues, discuss the challenges and how to address them we also remind the fieldworkers on the ethical issues for example about their own responsibilities | An interviewer and field staff can start as a field interviewer and then be a supervisor and then sometimes field manager. HR have their own arrangement but the structure is not formal |
| 8. | **Research Institution 8-EA** | 1996 | Malaria, HIV, Health and Poverty, TB | 800 | 183 | 22.9 | MOH, Swiss Institute, BMG, EU | FWs are given intensive training about how to work in the DSS and the procedures of doing the DSS activities. We also train them about ethics and their responsibilities. At the same time, other related issues concerning DSS activities are varied. | The training unit deals with all the training needs and capacity building. So a fieldworker can even be appointed to be a field supervisor or a field manager if they express interest. A structure is available |
| 9. | **Research Institution 9-EA** | 2011 | Health system research, Reproductive health | 250 | 100 | 75.6 | Wellcome Trust | New fieldworkers are paired with more experienced fieldworkers for a given period of time. They are also required to attend mandatory meetings to share their experiences. After every DSS round, a three days' refresher training is organized to recap on what they have done and solve any problems. | Training and capacity building is available for senior research staff but not for fieldworkers |
| 10. | **Research Institution 10-WA.** | 2005 | social scientist and clinical research | 90 | 50 | 50.0 | MOH & LSHTM | Training for field workers is coordinated by the leader of the study team so for every study, we have a team responsible for coordinating the fieldworkers training, who train them about field practices, community entry law and so on. | There is no formal structure for fieldworkers' career development. Only those with a degree and Masters qualification can be promoted to senior positions |
| 11. | **Research Institution 11-WA.** | 1994 | Malaria, TB, HIV, and Maternal and child health | 200 | 100 | 50.0 | LSHTM, The Kwame Nkrumah University of science and technology, University of Ghana | FWs receive training retraining on the forms they use in the field, at the end of the round and at the end of the year. Every week, they meet with their supervisors in the field to discuss any emerging issues. | Fieldworkers can be supported to undertake an advanced training in nursing or laboratory but there is no guarantee that their position would be reserved when they complete the course. A formal structure is not available |
| 12. | **Research Institution 12-EA.** | 1984 | Malaria, TB, HIV and emerging infection | 1500 | 630 | 42.0 | CDC, EDCTP and other agencies | Fieldworkers are trained on ethics, which entails Good Clinical Practice then they are paired up with more experienced ones and then trained on relevant processes and specific study document, tools and protocol training, general orientation, confidentiality issues and after this, they are left to | There are promotions on a yearly basis when a staff is being evaluated and other conditions which the staff should meet before the supervisor would recommend they get a promotion. No career progression framework for fieldworkers |
| 13. | **Research Institution 13-EA** | 1988 | Maternal and Newborn health | 380 | 105 | 27.6 | MRC UK | All fieldworkers undergo a GCP training and then trained on their specific study protocol | Career progression depends on studies. Different studies have different requirement for a career, some of them may directly be organized by the program for a certain cadre to go for training. We don't have a structure of career progression for fieldworkers |
| 14. | **Research Institution 14-SA** | 1995 | HIV and AIDS, Malaria, Non-communicable diseases | 300 | 73 | 24.3 | WT & EU | There is no standard structure. Once employed and taken through HR induction and study protocol, they are ready to go and work. There is no | No structure available but if there is a new position, a fieldworker who has the appropriate qualification can apply. There is no formal approach to supporting |

| | | | | | | | | | |
|---|---|---|---|---|---|---|---|---|---|
| | | | | | | | | process to build FWs capacity to address practical and ethical challenges FWs face. | fieldworkers. Sponsorship is competitive against all staff members |
| 15. | **Research Institution 15-SA** | 1979 | TB, HIV, NCD, Clinical and epidemiological research | 184 | 73 | 39.7 | WT, LSHTM, BMGF & EU | Fieldworkers get trained on demographic surveillance its basic outline and principles because most studies are linked to our Continuous Registration System (CRS). They are also trained on HIV counselling and testing and data entry. | No formal career path exists |
| 16. | **Research Institution 16-WA** | 1992 | Vaccine and Drug trials, fundamental sciences, Mosquitoes genetics, Parasites genetics, Drug Resistance (PCR, in Vitro tests…), Malaria Immunology, Malaria and Pregnancy trial | 45 | 30 | 66.7 | NIH, MOH and EDCTP | Fieldworkers are given protocol-specific training and told about their responsibilities and objectives of the study. | Training/Career Development Program is in place to increase the number of qualified scientific and technical personnel capable of conducting independent medical research. This includes post-doctoral training-PhD levels, Parasitology, Cellular Biology, Pharmacology, Immunology; Maters of Sciences: Clinical research, Clinical Pharmacology, Public Health, Parasitology, Entomology, Nutrition. No specific framework for fieldworkers |
| 17. | **Research Institution 17-WA.** | 1998 | Maternal and child health, tropical diseases; malaria, Reproductive Health, Communicable diseases; Non-communicable diseases; human genetics; Health systems research, Social and Behavioural Studies, Poverty and Equity Studies | 263 | 193 | 73 | Ghana Health Service, Rockefeller Foundation, Population Council- USA, USAID | Fieldworkers training depends on the nature of the project, so for instance, if you are doing a survey on the health system, you will be taken through that and the overview of the overall Centre, community engagement and ethics. The project staff and the PI undertake the training | If a junior/frontline staff goes for further studies and has worked for 10 or more years and the field of study is relevant to their line of work, then the Centre, they may be supported. There is no formal structure to support fieldworkers. |
| 18. | **Research Institution 18-WA.** | 2007 | Molecular biology, immunology, clinical biology and bioinformatics | Staff are contracted from other organizations | FWs are contracted through other organization on a need basis then laid off at the end of a study | Depends on the project | National government, UNI-AID, Liverpool University, NIH and Fogarty foundation | New fieldworkers are usually given a Standard Operational Procedure then shown what they are going to do after that, they can be allowed to work in the field. | FWs are contracted through other organization on a need basis then laid off at the end of a study |
| 19. | ***Note: A discussion with MRC-UK and the INDEPTH secretariat in Ghana was also held with a view to crosscheck the information gathered from the interviews held** | | | | | | | | |
| **Total- 7 Research Institution from West Africa, 4 Research Institution from Southern Africa and 7 Research Institution from East Africa and 1 regional research coordination institution from Africa and 1 UK research funding institution** | | | | | | | | | |



For peer review only - http://bmjopen.bmj.com/site/about/guidelines.xhtml

Each telephone interview lasted for approximately one and a half hours. During the interviews, we sought to develop an understanding of current institutional practices and experiences in relation to training, support supervision and performance management of fieldworkers. Interviews with field managers explored issues related to processes of recruiting fieldworkers, the minimum qualification for fieldworkers, and the requirements for professional training as well as internal and external training given to fieldworkers. Additionally, issues around capacity building, support supervision and professional development for fieldworkers were discussed. Interviews with senior fieldworkers focused on their perceptions of the roles they play in research, challenges and mechanisms for addressing these challenges, career development opportunities and other existing support systems. The first author conducted the interviews, which were voice recorded, transcribed and immediately read through for verification and filling of any gaps by the interviewer before analysis.

### *3.2.2 Phase 2: The workshop with field managers*

A total of Twenty-five managers from fifteen of the eighteen research Centres that took part in the Phase One telephone interviews attended the Two and a half days' workshop to further discuss the Phase One findings. In total, the fifteen institutions represented at the workshop employed a total of 6702 staff, with 2136 (31.9%) being fieldworkers. The institutions came from nine different African countries. To attend the workshop, invitations were sent directly to the individuals who participated in Phase One interviews and requested to seek clearance from their line managers before attending the workshop.

The workshop agenda was developed collaboratively between the study team and an independent Kenyan consultant who acted as the main workshop facilitator, and who had been selected based on his in-depth experience of participatory methods and prior familiarity with research institutions in Africa. Overall, the workshop combined information sharing and large group discussions on day one with a series of participatory consultative and consensus building activities over the following days. On day one, two sets of presentations were made and discussed in plenary. The first presentation described Phase One findings, with discussions on the range, strengths and challenges of fieldworkers' employment and support structures across institutions. The second presentation highlighted findings from earlier social science research in KWTRP and other research sites in Africa on the complex nature of fieldwork, including practical, social and ethical challenges that fieldworkers often face as part of their routine work,



For peer review only - http://bmjopen.bmj.com/site/about/guidelines.xhtml

particularly within low-income communities and householders. Discussions that followed the presentations focused on the relevance and importance of these challenges across sites.

For peer review only



For peer review only - http://bmjopen.bmj.com/site/about/guidelines.xhtml

## 4. DATA ANALYSIS

Data analysis in this study followed a Framework Analysis approach (13). Transcripts were independently read and discussed in-depth by two members of the study team (FK and VM) to develop an initial coding framework, drawing on the main questions raised during interviews (see Appendix A) and themes that emerged during discussions. FK used Nvivo 10 software to organize data across the transcripts under these codes, adding codes for new issues emerging from the data. Data analysis charts were developed by FK and VM to account for the codes under themes by the participants, and support interpretation of patterns within the responses, drawing on the social science and research ethics literature.

Preliminary analysis of the telephone survey data was used to inform about the type and range of fieldworkers' support supervision and training practices as well as performance management strategies. The themes generated from this initial analysis were explored further and discussed in detail during the consultative workshop, with a view for consensus building and checking for similarities and variations across different research institutions. Emerging new themes were also discussed and explored using a combination of qualitative participatory approaches, including large and small group discussions, round table and moderated discussions.

Moderators were careful to discuss ideas in detail without influencing the direction and outcome of the discussion. During the analysis, we compared and contrasted the fieldworkers' and field managers' views based on specific themes. Through this, we realized there were issues that fieldworkers and field managers shared the same views and others that they had different views. In this paper, we have tried to present these similarities and contracts together, rather than separately. We believe this will be much easier for our readers to follow than it would have been if we separated the views of fieldworkers from those of the field managers. Finally, although our participants are classified as fieldworkers and field managers, it is important to note that questions in the interview guides that were used to interview them were very similar. Despite the similarity of the interview guide used, the field managers interviews were considerable longer, allowing for a more in-depth exploration of issues. This makes it logical to analyze and present these data together as shown in the next section

## 5. FINDINGS

In this section, we describe and discuss findings from the telephone survey and where applicable, the highlights of the workshop, across three inter-related themes that were explored or emerged as important across this study. These themes are A) The current institutional



For peer review only - http://bmjopen.bmj.com/site/about/guidelines.xhtml

structures and systems that support fieldworkers across the institutions involved in the study; B) Participants' perceptions of work-related challenges for fieldworkers and their relationship to support structures and; C) Views on the ways in which challenges for fieldworkers might be addressed, including agreement on institutional and wider recommendations.

**5.1 Current Institutional structures and systems for fieldworkers**

Across interviews and the subsequent workshop, it became clear that there is a large amount of diversity in the names, tasks and institutional structures that govern and support the way that fieldworkers operate in the institutions involved. For example, during interviews, senior fieldworkers described the existence of many different tasks and titles, including fieldworker, field enumerator, field officer, tracer, community technician and field assistant, which were often used interchangeably, within and across different Centres. Similarly, the way that fieldworkers were recruited, contracted, trained and supported in their work was highly variable, between projects within an institution and between institutions. Table 1 describes these parameters across the research institutions involved.

As Table 1 shows, some form of in-house fieldworkers training was common across the institutions, with training mainly focused on the skills needed to undertake study-specific tasks, such as using particular data collection tools. In some cases, training in Good Clinical Practice (GCP) was also offered, particularly to support fieldworkers undertaking informed consent processes. Often, such training was generally the responsibility of individual study teams within institutions. Four out of the fifteen institutions (27%) confirmed having a centrally coordinated training approach, three of which covered a more comprehensive set of topics including data collection skills, communication skills, informed consent processes, GCP and research ethics more generally. The other sites described conducting some kind of *ad hoc* training based on a specific study requirement or had no training plans for fieldworkers.

One area of fieldworker performance management which was rarely discussed and generally under-recognized across institutions was the concept of fieldworkers' professional career progression or continuing professional development. While such measures were largely routine for other cadres of research staff within institutions, they had not been applied to fieldworkers. Many felt this would be an important step towards recognizing the important role fieldworkers play, and in motivating them to maintain a high professional standard of



For peer review only - http://bmjopen.bmj.com/site/about/guidelines.xhtml

work, but challenges were seen in sustaining such an initiative given the typically short-term contracts offered to fieldworkers, based on similarly short research funding cycles.

> *"It is good to establish a professional development scheme for fieldworkers. However, such a system may not be sustainable, given the dynamic employment terms of fieldworkers and researcher in general…So although it is a good concept, it may be difficult to implement it [the scheme] within the context of research that relies on funding that is not always guaranteed"* (FWM, SA[1])

## 5.2 Participants' perceptions of work-related challenges for fieldworkers and their relationship to support structures

Across the following paragraphs, we describe the forms of challenges that fieldworkers and those working closely with them described during interviews, and that were taken forward for discussion at the workshop. In describing these, we highlight the types of institutional support that influence these challenges, including structural influences from human resource management strategies and research funding cycles, and more *ad hoc* often individual efforts to manage complex challenges on the ground.

### *5.2.1 Using different 'labels' for fieldworkers*

Discussions during interviews and at the workshop drew out differences in opinions on the importance of the 'labels' that were used to describe the work we describe here as 'fieldwork'. At a most pragmatic level, some fieldworkers felt that the most important issue was having a job, rather than being concerned about the title that was associated with that job. Practically, this attitude seems to hint at potentially problematic power imbalances for fieldworkers in relation to the institutions in which they were employed. Other fieldworkers felt that using different 'labels', particularly within one institution, could generate confusion about roles and lead to employment conditions being less transparent:

> *"It [roles taken by field workers] is not different. These are the organisation's terms…community technician, field worker, follow up staff, field technician…I mean it is just the same thing…it's just their [the organisation] way of making us look different, but we are the same"*. (SFW, KE)

---

*[1] First three letters of the initials on the quote stand for the position of the interviewee i.e Senior Field Worker (SFW) and Field Worker Manager (FWM); and the last two letters represent the country eg Kenya (KN), South Africa (SA), Ghana (GN) etc*



For peer review only - http://bmjopen.bmj.com/site/about/guidelines.xhtml

Many field managers recognised the phenomenon of multiple labels as being used for slightly different forms of 'fieldwork'. There was, however, an initial divergence of opinion about the value of harmonising job titles. Some felt that this variation underlined important, even if small differences, in fieldworkers' current roles and prior experience or qualifications, which might not be obvious to all team members. Others felt there was little need for multiple titles, given the potential for confusion and conflict this might present. As will be described in the later section on recommendations, developing clear and strategic approaches to fieldworkers' roles within an institution emerged as an important feature of good practice for fieldworkers' employment. For many, harmonizing these structures and processes across institutions was also seen as valuable. Field managers felt harmonizing fieldworker employment terms, training and skills would promote cross-study and institution transfer of fieldworkers and address the challenge associated with high turnover of fieldworkers due to short-term contracts.

#### *5.2.2 Seeing fieldwork as a short-term activity*

A core tension that was strongly expressed in interviews and the workshop was a general lack of recognition for the work that fieldworkers undertake within research institutions. Fieldworkers themselves saw their work as important, and as fundamentally contributing to answering important research questions, promoting the reputation of institutions, careers of scientists and other cadres of research staff. There was, however, some frustration and perceived unfairness around the lack of opportunities for fieldworkers to progress within their area of work, or achieve recognition over sometimes periods of many years working in this role. Experiences were shared in which scientists who joined research centres were able to move ahead to higher positions through focused capacity building strategies, while fieldworkers' positions remained static. Some fieldworkers described working for an institution for more than ten years without more than basic training or promotion. Perhaps not surprising, given the way that 'fieldwork' has traditionally been viewed within research institutions (that is, as short-term and relatively non-technical work), the institutions involved in this study had not developed strategic approaches for fieldworkers' career development.

In response to these frustrations, some fieldworkers had taken up self-funded part-time training courses outside their existing jobs in the hope that this would allow them to progress within the institution. This signified the importance fieldworkers valued education and training as an important element of their career advancement. In practice, however, institutions did not have structures that would allow fieldworkers to move into roles in which such new skills could be used. Against this background, most fieldworkers reported feeling demotivated, including a feeling of low self-esteem and disillusionment:



For peer review only - http://bmjopen.bmj.com/site/about/guidelines.xhtml

*"Once you are employed as a fieldworker, you will always be a fieldworker* [regardless of your qualifications]". *(SFW, GN)*

While some field managers supported this view, some argued that career development was a matter of personal motivation and determination. They explained how they [managers] had moved through the career ladder to become field managers, often starting their careers as junior fieldworkers and gaining extensive fieldwork experience along their career progression to their current positions, with the same limited training and support. Most managers had therefore been part of the system for a long time and were able to share their views both from the perspective of fieldworkers as well as field managers hence extending the spectrum of experiences adduced and provide deeper insights to the issues that were raised

Given these challenges, which workshop participants saw as fundamentally unfair and as potentially importantly undermining the quality of research at its roots, participants agreed that fieldworkers should have greater recognition for the role they play within research institutions. They also agreed that it was important for institutions to support some form of career progression, for example, within a specialised category of staff supporting fieldwork. Providing fieldworkers with generic knowledge and skills on core research areas as part of such a scheme would enable them to transfer between studies at the end of a funding cycle. This would enable research centres to retain experienced fieldworkers, a policy that was thought likely to promote high scientific and ethical standards; and give fieldworkers more stability, skills and recognition.

### 5.2.3 Responding to complex challenges on the ground

In both the telephone interviews and the subsequent workshop, fieldworkers and managers described facing often very difficult challenges that seem to be implicit in the nature of much field work. Some of these were practical, including the physical burdens of fieldwork, such as the experience of long working days with long distances to cover on foot, risks of injury through dog or snake bites, and physical assault in less secure settings. Many also described emotional burdens including anxiety, fear, sadness and sometimes helplessness when interacting with individuals and families facing severe social and economic hardship. Within the community, fieldworkers said they were perceived as 'fellow community members' and, at the same time, the 'face' of the institutions that employ them, generating expectations of support that were beyond their mandates from the community members they visited (14).

These challenges have been well described in the literature on global health ethics, including from social science research at KWTRP, and our findings in this study underline the existence of these important challenges (1, 15-18). The fact that our study draws on experiences from 18 major research Centres in Africa and found similar challenges is an indication of how common



For peer review only - http://bmjopen.bmj.com/site/about/guidelines.xhtml

and important those challenges are. Within this literature, there is increasing recognition of the moral work that fieldworkers are involved in during their everyday work (3, 19-21) which can directly impact the science and ethics of the studies they work for (5, 22). For example, a key issue in the literature concerns the negotiation of informed consent processes, where fieldworkers balance perceived pressures to recruit participants according to targets set by managers and the extended period of time that may be needed to explain complex and often unfamiliar topics within consent forms to household members. In addition, community members may feel that by agreeing to participate in studies, they will develop some leverage to access greater institutional support, including through their relationships with fieldworkers (4, 5, 23-25). Similar ethical challenges experienced by fieldworkers have been reported in high income settings, including the United States of America (USA), with a specific focus on how fieldworkers' and study coordinators' practices can affect protection of research participants (26). Although it could be argued that these settings are contextually different, it would be interesting to examine why fieldworkers in High Income Countries (HIC) experience the same ethical challenges as those in Africa, despite them being better trained, educated and resourced than their counterparts.(2, 26)

A particularly difficult situation that fieldworkers involved in routine Demographic Surveillance System (DSS) activities in this study described was the need to directly interact with bereaved families and seek information about deceased relatives as part of conducting verbal autopsies (interviews held with close family members of a recently deceased person to try to establish the cause of death)(27, 28). Such emotionally challenging interactions generated distress and worry for fieldworkers and could chronically undermine performance, leading to 'burnout':

> *"Yeah but you see what, after maybe going through these challenges sometimes they affect you or they affect your output" [SFW, SA]*
>
> *I felt bad, and you know the following day I was to go back to work, and there is no supportive counselling or something like that… So as fieldworkers sometimes we just burn out and sometimes with this kind of trauma, it becomes difficult…we go through a lot*" [SFW, KE]

During workshop discussions about support mechanisms to help fieldworkers address these emotional challenges, field managers were not able to identify existing strategies; as was also reported by fieldworkers during the telephone interviews. Some managers described generally relying on fieldworkers' own personal judgements in handling these difficult situations.

> *"It is difficult to anticipate what challenges fieldworkers are likely to face…and even if they were to share them [the challenges] some of them are too complex to resolve. It is*



For peer review only - http://bmjopen.bmj.com/site/about/guidelines.xhtml

*therefore easier to just let them [fieldworkers] solve them spontaneously and trust that they use enough wisdom to do the right thing"* [FWM, KE]

Experiences shared by fieldworkers revealed that the issues were usually quite complex and intertwined with cultural, structural and emotional sensitivities associated with the need to balance community and cultural expectation against the policies and guidelines of research institutions. Thus, fieldworkers were often expected to strike a delicate balance, sometimes without knowledge of how acceptable their actions were. Despite these actions having the potential to directly affect the scientific and ethical standards of data that fieldworkers collect, structural support and referral systems were reportedly not available. This was well captured by one senior fieldworker who said.

"…. *sometimes, you get that huruma [pity], you go into your pocket and give out something…if you have a human heart, sometimes there are some situations that are touching"* [SFW, SA].

During interviews and workshop discussions, there were no accounts of existing internal or external institutional mechanisms to support fieldworkers to specifically deal with these types of emotional challenges. Although effective support supervision and sharing these challenges during team meeting was identified as a potential avenue to identify strategies to deal with these challenges, the approaches used to supervise fieldworkers were noted to be significantly different across institutions, and not centrally coordinated within institutions, making it difficult to reflect on their effectiveness. Workshop participants therefore generally felt there was a gap in fieldworkers' support structures that should be addressed. These challenges were seen as occurring across all the institutions involved in the study.

**5.3 Ways of strengthening institutional support**

Based on the outputs of discussions described so far, two core institutional fieldworker support strategies were raised, discussed and agreed upon over the course of the workshop. The first concerned adapting the *content of skill building initiatives* to support fieldworkers to manage their interactions with community members and household members in ways that could support good scientific and ethical practice, seen from multiple perspectives. The second strategic support mechanism addressed the wider question of how professional support within institutions could support fieldworkers more *structurally* towards these aims, including aspects of performance management that commonly exist for other groups of research staff. Workshop participants agreed that 'performance management' in this respect would include aspects of recruitment, training, supervision, support, assessment and career or professional development. Specifically, delegates emphasized the need to strengthen fieldworkers' interpersonal skills training to ensure they are well equipped to deal with cross-cutting issues around



For peer review only - http://bmjopen.bmj.com/site/about/guidelines.xhtml

communication, community engagement, building trust and respect, which are fundamental in consenting and quality data collection. Fieldworkers themselves believed that more support would improve their skills, and accordingly the quality of the data they collect. The need to develop a harmonized training curriculum, with core training modules across Africa was identified.

In this study, we noted a mismatch related to lack of appropriate training for fieldworkers; fieldworkers' limited level of education and the complexity of the tasks they are expected to undertake, including but not limited to translating study information to study participants, understanding institutional and international ethical guidelines and applying these when interacting with study participants. These findings support the need for research institutions in Africa to invest in training and building the capacity of fieldworkers, in order to enhance the standards of the work fieldworkers undertake and provide optimum support for research.

### 5.4 Agreement on recommendations

Given the issues discussed in the preceding paragraphs around institutional fieldworkers' support structures, participants at the workshop agreed there was an urgent need to introduce more standardized and comprehensive approaches, focused on important areas that affect fieldworkers' performance and practices, including career progression, training/capacity building and management of emotional and ethical challenges. It was felt that strategies could build on the positive experiences of research institutions that had already begun to institutionalize such approaches. Participants recommended a pragmatic approach, aimed at empowering and building the knowledge and skills of fieldworkers, including processes to support continuous learning through the regular reflection of the practical and ethical issues they face in everyday experience. In this way, an important element of training and support supervision was described as enhancing skills to recognise and respond to these ethical issues. The specific recommendations made are described and discussed in the following paragraphs.

#### *5.4.1 Creating space for experience sharing and problem-solving*

One challenge identified during the survey was lack of psychological support after experiencing difficult and sometimes traumatic experiences, such as some interviewees breaking down during verbal-autopsy interviews, losing a study participant when close relationships had been built and even being physically attacked by emotional/aggrieved participants during interviews. Long working days with long distances to cover on foot, risks of injury through dog or snake bites, and physical assault in less secure settings were also said to be emotionally overburdening to fieldworkers. Documented evidence shows that stressful and traumatic experiences can alter the way people view themselves, the world around them, and alter how they process information and the way they behave and respond to the environment



For peer review only - http://bmjopen.bmj.com/site/about/guidelines.xhtml

around them(29). A strategy described in the literature to address this challenge is the use of regular de-brief sessions, where fieldworkers are given opportunities to share experiences and consider the best approach to address challenges. During the workshop, participants recommended that field managers should create such opportunities for fieldworkers to talk about distressing or worrying issues encountered at work, through forms of feedback and reflection. This approach also requires a support supervision mechanism that is sensitive to the complex nature of the experiences fieldworkers face. This supports True's (2011) arguments that having access to colleagues to discuss issues and having effective support supervision acted as a buffering factor for research front-line staff who did not engage in ethical misconduct, despite facing the same pressures to meet recruitment goals and other stressors of conducting community-based research (30). Combining different ways of knowing and learning will enable fieldworkers to standardize the way they handle practical and ethical issues they encounter, even where these present as dilemmas with limited information on the best course of action. At the same time, in some cases, more traditional approaches to managing emotional distress, such as staff counselling, might also be needed.

#### *5.4.2 Increasing attention on soft skills for fieldworkers*

As reported by both fieldworkers and field managers, advancement has been made in the area of technical training for research staff, including fieldworkers. Good Clinical Practice, Health and Safety, Good Clinical Laboratory Practice (GCLP) training have become a standard approach for clinical study site preparation. While these trainings enable studies to adhere to international GCP/GCLP standards, they rarely equip fieldworkers with the necessary skills to enable them to deal with practical local challenges they face on the ground. There is, therefore, a need for fieldworkers to undergo more generic interpersonal skills training, such as communication skills, basic psychology and counselling and community engagement technics to enable them to appreciate the practical local situations and develop skills to deal with the ground challenges.

#### *5.4.3 Having career development guidelines that are responsive to fieldworkers*

In a previous commentary by Kombe et al (2015), that reported the views of field managers who attended a consultative workshop as part of this study, important recommendations made by the managers were presented, including the need to i) increase institutional recognition of fieldworker's roles and the need for systematic and comprehensive capacity building to ensure adequate resources are allocated and that capacity building activities are well described in research proposals and grant applications; ii) develop common areas of a core curriculum for



For peer review only - http://bmjopen.bmj.com/site/about/guidelines.xhtml

fieldworkers capacity building to enhance quality of training processes and build their knowledge in basic biology, research approaches and methods, research ethics and research regulatory frameworks as well as data collection and documentation; respectful communication, and being aware of and managing ethical challenges and issues in practice; and iii) increase emphasis on fieldworkers' career development including developing regionally accredited training to support fieldworkers' professional development to increase individual motivation and enhance capacity for employment across different research organizations (12). These recommendations should be considered in order to address the challenges presented in this paper and enhance the overall quality and integrity of research in Africa. Similar strategies have been proposed by True et al, to address challenges faced by community members employed as research staff in the USA (2)

## 6. DISCUSSION

The important role fieldworkers play in health research can be enhanced if fieldworkers are given adequate support. The challenges they face require a combination of knowledge, skills, attitude change and ethical values that can be enhanced through structured institutional support and instilling a sense of professional responsibility and moral values to fieldworkers; through ongoing support supervision, soft skills training and creating space for consultative discussions and experience sharing.

In this study, fieldworkers were reported to provide important support for research. From the institutions that participated in the study, fieldworkers were employed to undertake diverse tasks and were given a range of different titles. All participants that were interviewed acknowledged the fact that the tasks undertaken by fieldworkers were critical in supporting health research. On the other hand, Fieldworkers reported experiencing a myriad of challenges, a fact that was supported by the fieldworker managers interviewed, and strongly deliberated upon during the workshop, during the second phase of this study.

Despite the above perceived importance of the tasks fieldworkers undertake in supporting health research, there were no centrally and institutionally coordinated systems or structures that were reported to have been put in place to provide the much-needed support for fieldworkers in most of the institutions involved in the study. Fieldworkers coordination and support were largely left in the hands of study specific field managers and supervisors, who often focused on the outputs of the specific studies they coordinated, with much less focus on generic support and supervision systems. Institutional systems to support fieldworkers to undertake their tasks effectively were reported to be limited or often unavailable. Furthermore, in spite of the myriad of challenges fieldworkers reported to experience in their day to day



For peer review only - http://bmjopen.bmj.com/site/about/guidelines.xhtml

activities, which were confirmed by their field managers during the telephone interviews and the consultative workshop, no institutional systems were reported to be in place to support fieldworkers in addressing such challenges. Fieldworkers were therefore, essentially expected to use their discretion, cultural competence and moral reasoning to navigate through the many practical and ethical challenges they faced. Given the limited support, education and ethics knowledge and competence fieldworkers possessed, this situation, points to serious implications on the quality of the data fieldworkers collect, their ethical practice and the overall integrity of the health research enterprise fieldworkers are expected to support.

This study interviewed participants via telephone. Although this could not have necessarily affected the quality of the data collected, important qualitative interview dynamics, which can provide important insights on the data analysis process could not be identified and considered. However, the triangulation of different study approaches, including the consultative workshop with field managers, provided an important approach for validating the data collected and strengthening the study design.

The results from this study underscores the need to establish and strengthen institutional support for health research fieldworkers. Although a lot of studies have been done to understand the moral and ethical challenges and dilemmas faced by fieldworkers, very little has been done to explore which fieldworkers' institutional support systems exist and what implications such systems have on fieldworkers' scientific and ethical practices. There is therefore a need for further research, in order to develop a deeper understanding around this area. This study has highlighted important gaps related to the management and coordination of fieldworkers, that require further assessment and strategic consideration. Furthermore, the strategic solutions and recommendations identified in this study call for the need for health research institutions in Africa and funders of health research to strongly consider developing institutional systems and structures to facilitate centralized coordination and management of fieldworkers by health research institutions in Africa. As stated by Alexander and Richman, strengthening the capacity of fieldworkers has the potential to increase the protection of research participants, enhance research integrity and promote the collection of valid data (31). Efforts to empower fieldworkers to make them better able to deal with these challenges should therefore continue.

This was an exploratory study hence the results are not generalizable. However, they do offer important insights on fieldworkers and field managers' views and experiences of fieldworkers' performance managing practices for institutions that come from similar settings as those that participated in this study. Although some of the proposed approaches such as interpersonal skills training, centralized coordination of fieldworkers training, fieldworkers' scheme of



For peer review only - http://bmjopen.bmj.com/site/about/guidelines.xhtml

service and regular discussions to address fieldwork challenges have been ongoing in institutions such as the KWRTP, in Kenya, lack of documented evidence makes it difficult to say how effective and sustainable such approaches are. More research, therefore, needs to be done to explore the feasibility of the proposed strategies and generalizability of these study findings within a wider context in Africa. Finally, the proposed solutions may not solve all the challenges fieldworkers face, but they are certainly an important step towards improving the scientific and ethical standards of health research output from the African continent. The value of this study is not that every research centre in Africa can identify with every issue raised here, this would be difficult due to the variety of research centres in the continent, rather, we hope that by raising the issues highlighted here, even those centre that are not involved in the study will be provoked to examine their fieldworkers support system and see if any issues raised here apply to their setting. Furthermore, empirical studies should be done to identify which approaches are most effective in enabling research fieldworkers to deal with the myriad of challenges they face. Such research will go a long way in improving the scientific and ethical standards research, not only in Africa but internationally, given the critical global role of Africa in research.

## 7. DATA AVAILABILITY STATEMENT

Data are available in the form of transcripts that are currently archived in password protected computers. All the transcripts are anonymized, to avoid identification of the participants who were interviewed. Permission was obtained to use the data for purposes of this study only. No permission was granted to use the data for future research.

For peer review only



For peer review only - http://bmjopen.bmj.com/site/about/guidelines.xhtml

## 8. REFERENCES

1. Kamuya DM, Theobald SJ, Munywoki PK, Koech D, Geissler WP, Molyneux SC. Evolving friendships and shifting ethical dilemmas: fieldworkers' experiences in a short term community based study in Kenya. Dev World Bioeth. 2013;13(1):1-9.
2. True G, Alexander LB, Fisher CB. Supporting the role of community members employed as research staff: Perspectives of community researchers working in addiction research. Social Science & Medicine. 2017;187:67-75.
3. Kamuya DM, Theobald SJ, Marsh V, Parker M, Geissler WP, Molyneux SC. “The one who chases you away does not tell you go”: silent refusals and complex power relations in research consent processes in Coastal Kenya. PloS one. 2015;10(5):e0126671.
4. Chantler T, Otewa F, Onyango P, Okoth B, Odhiambo F, Parker M, et al. Ethical Challenges that Arise at the Community Interface of Health R esearch: Village R eporters’ Experiences in Western K enya. Developing world bioethics. 2013;13(1):30-7.
5. Kingori P. Experiencing everyday ethics in context: Frontline data collectors perspectives and practices of bioethics. Social science & medicine. 2013;98:361-70.
6. Madiega PA, Jones G, Prince RJ, Geissler PW. ‘She's my sister-in-law, my visitor, my friend’–challenges of staff identity in home follow-up in an HIV trial in Western Kenya. Developing world bioethics. 2013;13(1):21-9.
7. Marshall PA, Marshall PL. Ethical challenges in study design and informed consent for health research in resource-poor settings: World Health Organization; 2007.
8. Richman KA, Alexander LB, True G. Proximity, ethical dilemmas, and community research workers. AJOB Primary Research. 2012;3(4):19-29.
9. Lansang MA, Dennis R. Building capacity in health research in the developing world. Bulletin of the World Health Organization. 2004;82(10):764-70.
10. Glickman SW, McHutchison JG, Peterson ED, Cairns CB, Harrington RA, Califf RM, et al. Ethical and scientific implications of the globalization of clinical research. New England Journal of Medicine. 2009;360(8):816-23.
11. Participants of an International Workshop in Kenya on the Role of Frontline Staff in Biomedical Research J, Kombe F. Enhancing quality and integrity in biomedical research in Africa: an international call for greater focus, investment and standardisation in capacity strengthening for frontline staff. BMC Med Ethics. 2015;16(1):77.
12. Kombe F. Enhancing quality and integrity in biomedical research in Africa: an international call for greater focus, investment and standardisation in capacity strengthening for frontline staff. BMC medical ethics. 2015;16(1):77.
13. Srivastava A, Thomson SB. Framework analysis: a qualitative methodology for applied policy research. 2009.
14. Molyneux S, Kamuya D, Madiega PA, Chantler T, Angwenyi V, Geissler PW. Field workers at the interface. Developing world bioethics. 2013;13(1):ii.
15. Kingori P. Experiencing everyday ethics in context: frontline data collectors perspectives and practices of bioethics. Social science & medicine. 2013;98:361-70.
16. Molyneux S, Kamuya D, Madiega PA, Chantler T, Angwenyi V, Geissler PW. Field workers at the interface. Dev World Bioeth. 2013;13(1):ii-iv.
17. Chantler T, Otewa F, Onyango P, Okoth B, Odhiambo F, Parker M, et al. Ethical challenges that arise at the community interface of health research: village reporters' experiences in Western Kenya. Dev World Bioeth. 2013;13(1):30-7.
18. Angwenyi V, Kamuya D, Mwachiro D, Marsh V, Njuguna P, Molyneux S. Working with Community Health Workers as 'volunteers' in a vaccine trial: practical and ethical experiences and implications. Dev World Bioeth. 2013;13(1):38-47.
19. Kamuya DM, Theobald SJ, Munywoki PK, Koech D, Geissler WP, Molyneux SC. Evolving Friendships and Shifting Ethical Dilemmas: Fieldworkers’ Experiences in a Short Term Community Based Study in K enya. Developing world bioethics. 2013;13(1):1-9.



For peer review only - http://bmjopen.bmj.com/site/about/guidelines.xhtml

20. Wax ML. On fieldworkers and those exposed to fieldwork: Federal regulations and moral issues. Human Organization. 1977;36(3):321.
21. Zinn MB. Field research in minority communities: Ethical, methodological and political observations by an insider. Social problems. 1979;27(2):209-19.
22. Kombe F, Anunobi EN, Tshifugula NP, Wassenaar D, Njadingwe D, Mwalukore S, et al. Promoting Research Integrity in A frica: An African Voice of Concern on Research Misconduct and the Way Forward. Developing world bioethics. 2014;14(3):158-66.
23. Molyneux S, Kamuya D, Marsh V. Community members employed on research projects face crucial, often under-recognized, ethical dilemmas. The American Journal of Bioethics. 2010;10(3):24-6.
24. Mosavel M, Ahmed R, Daniels D, Simon C. Community researchers conducting health disparities research: Ethical and other insights from fieldwork journaling. Social science & medicine. 2011;73(1):145-52.
25. Simon C, Mosavel M. Community members as recruiters of human subjects: Ethical considerations. The American Journal of Bioethics. 2010;10(3):3-11.
26. Davis AM, Hull SC, Grady C, Wilfond BS, Henderson GE. The invisible hand in clinical research: The study coordinator's critical role in human subjects protection. The Journal of Law, Medicine & Ethics. 2002;30(3):411-9.
27. Zahr CA, Pinchuk M. Verbal autopsy standards: ascertaining and attributing cause of death: World Health Organization; 2007.
28. Fottrell E, Byass P. Verbal autopsy: methods in transition. Epidemiologic reviews. 2010;32(1):38-55.
29. Steele W. Trauma's Impact on Learning. Trauma and Loss: Research and Interventions. 2002.
30. True G, Alexander LB, Richman KA. Misbehaviors of front-line research personnel and the integrity of community-based research. J Empir Res Hum Res Ethics. 2011;6(2):3-12.
31. Alexander LB, Richman KA. Ethical Dilemmas in Evaluations Using Indigenous Research Workers. American Journal of Evaluation. 2008;29(1):73-85.

For peer review only



For peer review only - http://bmjopen.bmj.com/site/about/guidelines.xhtml

## Contributor and guarantor information

All authors were involved in conceptualizing the idea and contributed in writing the manuscript. Mr Francis Kombe (MPH), a male senior community facilitator with a wealth of experience in health research ethics, community engagement and communication skills, who worked as a fieldworker himself, before taking a managerial role at KWTRP, contributed to the planning, conduct, and reporting of the work described in the article and is responsible for the overall content. Prof Vicki Marsh (PhD), a female senior social scientist at KWTRP and Oxford University and Ms Dorothy Ikamba (Msc), a female career development officer at KWTRP, and career counsellor, assisted with reviewing the transcripts and the development of themes and sub-themes during the analysis. Prof Sassy Molyneux (PhD), a female senior social scientist at KWTRP and Oxford University, Dr Dorcas Kamuya (PhD), a female social scientist at KWTRP and Wellcome Trust Society & Ethics Fellow, and Dr Samson Kinyanju, a male immunologist and head of training at the KWTRP, contributed in designing the initial research ideas, reviewed the data collected and were involved in drafting the initial manuscript and edited subsequent versions. All authors reviewed and approved the final manuscript for publication. Francis Kombe accepts full responsibility for the work and the conduct of the study. He had full access to the data, and controlled the decision to publish. Francis Kombe attests that all listed authors meet authorship criteria and that no others meeting the criteria have been omitted.

## Copyright/license for publication

Francis Kombe has the right to grant on behalf of all authors and does grant on behalf of all authors, a worldwide license to the Publishers and its licensees in perpetuity, in all forms, formats and media (whether known now or created in the future), to i) publish, reproduce, distribute, display and store the Contribution, ii) translate the Contribution into other languages, create adaptations, reprints, include within collections and create summaries, extracts and/or, abstracts of the Contribution, iii) create any other derivative work(s) based on the Contribution, iv) to exploit all subsidiary rights in the Contribution, v) the inclusion of electronic links from the Contribution to third party material where-ever it may be located; and, vi) license any third party to do any or all of the above



For peer review only - http://bmjopen.bmj.com/site/about/guidelines.xhtml

## Transparency statement

Francis Kombe affirms that the manuscript is an honest, accurate, and transparent account of the study being reported; that no important aspects of the study have been omitted; and that any discrepancies from the study as originally planned have been explained.

## Competing interests

The views on this paper are the authors own viewpoints and do not represent the position of their organizations. The authors declare no conflict of interests.

The authors declared that they have no competing interests.

## Ethical approval

The paper is published with the permission of the Centre director, KEMRI. All the participant who participate in the telephone survey as well as those who attended the consultative workshop gave individual consent to participate in the study and the workshop, and permitted the publication of this manuscript. Permission to conduct this study was granted by a review conducted by the Centre for Geographic Medicine Research, Coast (CGMRC)- Centre Specific Committee and a final Ethical approval given by the Research and Scientific Review Unit (SERU) at the Kenya Medical Research Institute (KEMRI).

For peer review only



For peer review only - http://bmjopen.bmj.com/site/about/guidelines.xhtml

**DISCUSSION GUIDE FOR RESEARCH INSTITUTIONS WORKING WITH FIELDWORKERS (FWs) IN SSA**

**TITLE: STRENGTHENING TRAINING OF FRONT LINE (FWs) STAFF AS A MEANS TO ENHANCE COMMUNITY ENGAGEMENT**

**INFORMATION SHEET FOR MANAGERS AND SENIOR FRONTLINE STAFF**

## What is this survey all about?

This survey will be conducted through telephone/ teleconference discussion. One front line staff manager (people who have a direct role of providing support supervision to frontline staff) and one senior frontline staff member from a total of 20 health research institutions in Sub-Saharan Africa will be invited to participate in the discussion.

The aim of the discussion is to identify practical and ethical challenges experienced by frontline staff in their day to day activities and the available support supervision, performance management and Continuing Professional Development (CPD) practices within these institutions. Later, representatives from a few of the 20 institutions will be invited to a consultative workshop to follow up and discuss these issues in detail. The report generated from the survey and workshop will be shared with all institutes for their own use. In the long term, the report could be used to inform further discussions on the potential of developing a generic curriculum for frontline staff training.

## What is expected of you if you take part in this discussion?

If you agree to take part in this discussion, we will request you to select a date and time when you would prefer the moderator to call and have the discussion with you. We will then call and ask a few questions with regards to your perceptions of the roles of frontline staff and the institutional support supervision, monitoring and evaluation and general CPD practices. These questions are shown on the attached discussion guide for your attention. The discussion is expected to take a maximum of 30 minutes.

Participation in this interview is voluntary. While it is important to answer all the questions, feel free to skip any question that you do not feel comfortable to answer and the moderator will continue asking the next question. You can also decide to terminate the discussion at any time if you decide not to continue with the discussion for any reason.

## Is there any individual benefit for taking part in this discussion?

There is no individual benefit in taking part in this discussion. However, by sharing your experiences in working with frontline staff, you will assist in contributing to knowledge that could improve the way frontline staff deal with their practical and ethical challenges as well as their support supervision, performance and general CPD management practices in future.

## If you have any question

If you have any question about this survey, please contact any of the following people through the email addresses provided below

| | | |
|---|---|---|
| Francis Kombe<br>KWTRP<br>P.O BOX 230, KILIFI<br>Fkombe@kemri-wellcome.org | Sam Kinyanjui<br>KWTRP<br>P.O BOX 230, KILIFI<br>Skinyajui@kemri-wellcome.org | Dorcas Kamuya<br>KWTRP/ Ethox-Nuffield Department of Population Science, Oxford University<br>P.O BOX 230, KILIFI<br>Dkamuya@kemri-wellcome.org |

For peer review only

**DISCUSSION GUIDE FOR RESEARCH INSTITUTIONS WORKING WITH FIELDWORKERS (FWs) IN SSA**

**TITLE: STRENGTHENING TRAINING OF FRONT LINE (FWs) STAFF AS A MEANS TO ENHANCE COMMUNITY ENGAGEMENT**

## SECTION A: Institutional Background

1. Name of organization ______________________________________________
2. Vision ______________________________________________
3. Mission ______________________________________________
4. Motto ________________________________________
5. Year organization was established [__][__][__] [__]
6. Source of main funding for the organization ________________________________________
7. Other funding agencies and collaborators ________________________________________
8. Types of research conducted at the organization: ______________________________________

## SECTION B: Frontline staff Managers' Perspective[1]

*This section explores managers' perceptions of the roles of and practical and ethical challenges faced by interface staff when discharging their duties and the institutional support supervision, performance management and Continuing Professional Development (CPD) practices*

1. What names are given to your frontline staff whose primary role involves recruiting and consenting research participants into studies?

2. What key roles/activities do they undertake in your organization?

3. In your institution, how many staff does it have (in total) and how many of these are employed as frontline staff?

4. What is the range of qualifications for frontline staff and what is their minimum level of education/qualification?

5. What is the nature of their employment e.g. are they in contract? What is the average contract period? Are the contracts renewable and what is considered during renewal? Any systems that ensure retention of frontline staff in the institutions?

6. What systems are in place to coordinate the way frontline staff are employed and retained across different studies in the institutions? What is the role of PI (if any) in retaining frontline staff?

7. What formal and informal processes are in place to induct new frontline staff when they first arrive, and to train them for their roles?

[1] Manager is the person whose primary responsibility involves working directly with front line staff

For peer review only - http://bmjopen.bmj.com/site/about/guidelines.xhtml

**DISCUSSION GUIDE FOR RESEARCH INSTITUTIONS WORKING WITH FIELDWORKERS (FWs) IN SSA**

**TITLE: STRENGTHENING TRAINING OF FRONT LINE (FWs) STAFF AS A MEANS TO ENHANCE COMMUNITY ENGAGEMENT**

---

*8.* How is the training for interface staff organized? Are there any refresher trainings/skills improvement activities? What are the *training approach eg frequency and objectives of the training,*

*9.* How is the frontline staff training managed? Describe funding, design, whether centralized or decentralized, and monitoring and evaluation of the training

*10.* Describe any practices, experiences and studies (if any) that have informed the current training eg feedback mechanisms; availability of a community engagement strategy, community Knowledge Attitude and Perception (KAP) surveys etc

*11.* How do you ensure skills learned are assimilated into the frontline's day to day activities? Are there any support supervision mechanisms, performance appraisals etc ? How are these implemented?

*12.* Describe any career progression scheme used by your organization for these staff groups

*13.* What challenges do you experience in working with and managing frontline staff?

**SECTION C: Frontline staffs' Experience-Staff perspective**

*This section explores frontline staff's practical and ethical challenges faced as they discharge their duties and their perceptions of the institutional support in training and CPD*

1. What are roles and responsibilities of frontline staff?

2. What challenges do frontline staff face when interacting with community members? *Practical and ethical challenges/dilemmas faced, e.g. issues of confidentiality?*

3. What type of in-house and external training is given to frontline staff in your institution?
   - ***Which of these do you perceive to be most appreciated and for what reason?***

4. What are the perceived institutional challenges in supporting and capacity building of frontline staff?
   - ***How do frontline members of staff address the above challenges?***

**SECTION D: Planned Workshop**

At the end of this survey, we hope to invite a selected number of the institutions to a 2 day workshop. Kindly indicate if you would like to be considered to attend this workshop.

For peer review only

# COREQ (COnsolidated criteria for REporting Qualitative research) Checklist

A checklist of items that should be included in reports of qualitative research. You must report the page number in your manuscript where you consider each of the items listed in this checklist. If you have not included this information, either revise your manuscript accordingly before submitting or note N/A.

| Topic | Item No. | Guide Questions/Description | Reported on Page No. |
|---|---|---|---|
| **Domain 1: Research team and reflexivity** | | | |
| *Personal characteristics* | | | |
| Interviewer/facilitator | 1 | Which author/s conducted the interview or focus group? | 22 |
| Credentials | 2 | What were the researcher's credentials? E.g. PhD, MD | 22 |
| Occupation | 3 | What was their occupation at the time of the study? | 22 |
| Gender | 4 | Was the researcher male or female? | 22 |
| Experience and training | 5 | What experience or training did the researcher have? | 22 |
| *Relationship with participants* | | | |
| Relationship established | 6 | Was a relationship established prior to study commencement? | 6 |
| Participant knowledge of the interviewer | 7 | What did the participants know about the researcher? e.g. personal goals, reasons for doing the research | 6 |
| Interviewer characteristics | 8 | What characteristics were reported about the inter viewer/facilitator? e.g. Bias, assumptions, reasons and interests in the research topic | 3, 10, 22 |
| **Domain 2: Study design** | | | |
| *Theoretical framework* | | | |
| Methodological orientation and Theory | 9 | What methodological orientation was stated to underpin the study? e.g. grounded theory, discourse analysis, ethnography, phenomenology, content analysis | 5 |
| *Participant selection* | | | |
| Sampling | 10 | How were participants selected? e.g. purposive, convenience, consecutive, snowball | 5 |
| Method of approach | 11 | How were participants approached? e.g. face-to-face, telephone, mail, email | 5 |
| Sample size | 12 | How many participants were in the study? | 5 |
| Non-participation | 13 | How many people refused to participate or dropped out? Reasons? | 5 |
| *Setting* | | | |
| Setting of data collection | 14 | Where was the data collected? e.g. home, clinic, workplace | 8 |
| Presence of non-participants | 15 | Was anyone else present besides the participants and researchers? | 5 |
| Description of sample | 16 | What are the important characteristics of the sample? e.g. demographic data, date | 6 |
| *Data collection* | | | |
| Interview guide | 17 | Were questions, prompts, guides provided by the authors? Was it pilot tested? | 8 |
| Repeat interviews | 18 | Were repeat inter views carried out? If yes, how many? | 8-10 |
| Audio/visual recording | 19 | Did the research use audio or visual recording to collect the data? | 8 |
| Field notes | 20 | Were field notes made during and/or after the inter view or focus group? | 8 |
| Duration | 21 | What was the duration of the inter views or focus group? | 8 |
| Data saturation | 22 | Was data saturation discussed? | |
| Transcripts returned | 23 | Were transcripts returned to participants for comment and/or | |

For peer review only

| Topic | Item No. | Guide Questions/Description | Reported on Page No. |
|---|---|---|---|
| | | correction? | |
| **Domain 3: analysis and findings** | | | |
| *Data analysis* | | | |
| Number of data coders | 24 | How many data coders coded the data? | 9 |
| Description of the coding tree | 25 | Did authors provide a description of the coding tree? | |
| Derivation of themes | 26 | Were themes identified in advance or derived from the data? | 9-10 |
| Software | 27 | What software, if applicable, was used to manage the data? | 9 |
| Participant checking | 28 | Did participants provide feedback on the findings? | 9 |
| *Reporting* | | | |
| Quotations presented | 29 | Were participant quotations presented to illustrate the themes/findings? Was each quotation identified? e.g. participant number | 10-16 |
| Data and findings consistent | 30 | Was there consistency between the data presented and the findings? | |
| Clarity of major themes | 31 | Were major themes clearly presented in the findings? | 10-16 |
| Clarity of minor themes | 32 | Is there a description of diverse cases or discussion of minor themes? | 10-16 |

Developed from: Tong A, Sainsbury P, Craig J. Consolidated criteria for reporting qualitative research (COREQ): a 32-item checklist for interviews and focus groups. *International Journal for Quality in Health Care*. 2007. Volume 19, Number 6: pp. 349 – 357

**Once you have completed this checklist, please save a copy and upload it as part of your submission. DO NOT include this checklist as part of the main manuscript document. It must be uploaded as a separate file.**

For peer review only

BMJ Open

# Enhancing Fieldworkers' Performance Management Support in Health Research: An exploratory study on the views of Field managers and Fieldworkers from major research centres in Africa

| | |
|---|---|
| Journal: | *BMJ Open* |
| Manuscript ID | bmjopen-2018-028453.R4 |
| Article Type: | Original research |
| Date Submitted by the Author: | 04-Nov-2019 |
| Complete List of Authors: | Kombe, Francis; Centre for Geographic Medicine Research Coast; African Research Integrity Network<br>Marsh, V; Centre for Geographic Medicine Research Coast; Centre for Tropical Medicine and Global Health, Nuffield Department of Medicine, Oxford University, UK<br>Molyneux, Sassy ; Centre for Geographic Medicine Research Coast; Centre for Tropical Medicine and Global Health, Nuffield Department of Medicine, Oxford University, UK<br>Kamuya, Dorcas; KEMRI Wellcome Trust Research Programme; Ethox Centre, Nuffield Department of Population Health, Oxford University, UK<br>Ikamba, Dorothy; KEMRI Wellcome Trust Research Programme<br>Kinyanjui, Samson; Centre for Geographic Medicine Research Coast; Centre for Tropical Medicine and Global Health, Nuffield Department of Medicine, Oxford University, UK |
| <b>Primary Subject Heading</b>: | Ethics |
| Secondary Subject Heading: | Ethics, Qualitative research |
| Keywords: | Fieldworkers, Africa, Capacity Building, Health Research, Research Ethics, Research Integrity |
| | |

SCHOLARONE™
Manuscripts

# Enhancing Fieldworkers' Performance Management Support in Health Research: An exploratory study on the *views of Field managers and Fieldworkers from major research centres in Africa*

Francis Kombe[1], Vicki Marsh[1,3], Sassy Molyneux[1,3], Dorcas Kamuya[1,2], Dorothy Ikamba[1], Kinyanjui Samson Muchina[1,3]

[1]KEMRI Wellcome Trust Research Programme, Kenya; [2]Ethox Centre, Nuffield Department of Population Health, Oxford University, UK; [3]Centre for Tropical Medicine and Global Health, Nuffield Department of Medicine, Oxford University, UK.

**Corresponding author**: Francis Kombe, KEMRI Wellcome Trust Research Programme, PO Box 230, Kilifi 80108, Kenya. Mobile +254 722459045; Email-Kajoleh@gmail.com

**Acknowledgements**: We acknowledge and greatly appreciate the support of all research institutions' directors who allowed their field managers and field workers to take part in this project, and all the field managers and fieldworkers who participated. We are extremely indebted to Prof Mike Parker, the director of the Wellcome Centre for Ethics and Humanities and director of Ethox Centre at the University of Oxford for his support and guidance during the project; and all the senior field managers and delegates who attended the consultative workshop. The manuscript is published with the support of the director, KEMRI.

**Funding**:



For peer review only - http://bmjopen.bmj.com/site/about/guidelines.xhtml

This project was funded by Global Health Bioethics Network-UK (Wellcome Trust grant no. OXF-GHB02) and supplemented by KWTRP Capacity Building Strategic Award (Wellcome Trust grant no 084538). The research and capacity building activities of the Global Health Bioethics Network are supported by a Wellcome Trust Strategic Award (096527)

**Keywords:** Africa, Fieldworkers, Research Integrity, Research Ethics, Capacity Building.

**Word Count: 7270**

For peer review only

## ABSTRACT

### Introduction

Fieldworkers are part of the system that promotes scientific and ethical standards in research, through data collection, consenting and supporting research, due to their insider cultural knowledge and fluency in local languages. The credibility and integrity of health research, therefore, rely on how fieldworkers adhere to institutional and research procedures and guidelines.

### Objectives

This study mapped out existing practices in training, support and performance management of fieldworkers in Africa, described fieldworkers and their managers' experiences, and lessons learned. A consultative process, involving field managers from 15 international health research institutions, was used to identify appropriate ways of addressing the challenges fieldworkers face.

### Methods

**Phase one-** We conducted 32 telephone interviews with 20 field managers and 12 senior fieldworkers from 18 major research centres in Africa, MRC-UK and the INDEPTH Network Secretariat.

**Phase two-** We held a two-and-a-half days workshop involving 25 delegates including 18 field managers from the institutions that were involved in phase one; and 7 additional stakeholders from the KWTRP. An earlier report from phase one was published in BMC Med Ethics in 2015. Data transcribed from the interviews and workshop proceedings were analyzed thematically using Nvivo 10 software.




For peer review only - http://bmjopen.bmj.com/site/about/guidelines.xhtml

## Results

Most institutions employed fieldworkers, usually with 12 years of formal education and residing within the geographic areas of research, to support studies. Although their roles were common, there were marked differences in the type of training, professional development schemes and fieldworkers support. Fieldworkers faced various challenges, with the potential to affect their ethical and scientific practices.

## Discussion

Fieldworkers undertake vital tasks that promote data quality and ethical practice in research. There is a need for research institutions to develop a structured support system, provide fieldworkers with interpersonal-skills training and provide space for discussion, reflection and experience-sharing to help fieldworkers tackle the practical and ethical challenges they face.

## Strengths and limitations of this study

### Strengths

- The fact that participants of this study were drawn from West, South and East Africa makes the results of this study rich with an extended spectrum of experiences adduced, which provide deeper insights to the issues that were discussed.
- The use of consultative approaches, that allowed issues raised during telephone interviews to be followed up and discussed further in a workshop, provided a unique opportunity to cross-check data from fieldworkers with their managers,

For peer review only

reflect on and unpack important issues, to identify relevant and applicable strategies for addressing challenges faced.

## Limitations

- Because all interviews were done by phone, important interview dynamics such as non-verbal cues may have been missed.
- Because our interviews focused on fieldworkers' challenges and potential opportunities, this could have raised the fieldworkers' expectation to receive direct support to address the challenges they faced and could have contributed to overreporting of the challenges they experienced.
- Because all interviewees were nominated by centre directors, who were the primary contact people, it is possible that interviewees could have reported what they believed would be acceptable to their directors due to power differentials between the participants (field managers and fieldworkers), and the centre directors.

For peer review only



For peer review only - http://bmjopen.bmj.com/site/about/guidelines.xhtml

# 1. BACKGROUND

High-quality data is a critical output of all science worldwide. Thus, research institutions put significant emphasis on generating data that are of the highest scientific and ethical standards. In many research programmes in low and middle-income countries, much of the data is collected by frontline staff referred to as “fieldworkers”. This cadre of staff is also often responsible for recruiting potential research participants, administering consent forms and following up participants during studies (1). As such, beyond ensuring data integrity, fieldworkers play a key role in ensuring the ethical implementation of research protocols. The role of fieldworkers is particularly critical in international collaborative programmes with researchers drawn from outside local communities. In such settings, fieldworkers, who are normally from the local communities, can serve as the face of research programmes with the potential to significantly influence communities’ trust in research.

In carrying out these complex roles, fieldworkers face practical and ethical dilemmas that require the balancing of institutional roles and guidelines against community expectations and fieldworkers' interests (2). Hence, fieldworkers continuously make independent (often unconscious) decisions on how to apply ‘ethical principles’ on the ground. For example, fieldworkers reported challenges such as dealing with ‘silent refusals’ (3) (where participants avoid key study procedures but do not openly refuse), and with many requests for health and other social-economic support that are not included in the study protocol (4-8).

For peer review only

### 1.1 Capacity Building and Investment In Research

Over the years there has been an increasing interest and investment in developing research capacity in Africa and other low and middle income regions through long-term initiatives such as the WHO- Tropical Diseases Research Programme (TDR) (9), the European and Developing Countries Clinical Trials Initiative Partnership (EDCTP) and more recently, The Wellcome Trust-DFID funded "Developing Excellence in Leadership, Training and Science "scheme (DELTAS) Africa. Efforts to strengthen research capacity are likely to continue, as the trend towards greater cross-border data sharing, globalization of clinical research and comparability of data quality at the global level continues (10). Given that fieldworkers play such a vital role in health research, it would be expected that research programmes, in addition to training scientists, would also focus on building fieldworkers' capacity to carry out their duties in the most effective manner. However, fieldworkers' capacity and skillsets have received relatively little attention. The mechanisms and processes by which research institutions in Africa support fieldworkers in undertaking their duties remain unclear. Apart from the Good Clinical Practice guidelines, which focus primarily on clinical trials, no other guidelines are available that provide a framework for the content and approach for supporting the continuing professional development and career growth of fieldworkers. Understanding the challenges that fieldworkers face and the type of systems, and level of support research institutions in Africa provide, can enable us to identify opportunities for strengthening fieldworkers' scientific and ethical practices.



For peer review only - http://bmjopen.bmj.com/site/about/guidelines.xhtml

In this paper, we draw on our results from a two-phase qualitative exploratory study on the perceptions, experiences and views of field managers and fieldworkers on the structural and performance management support extended to health research fieldworkers with the aim of informing institutional policies that promote research integrity and ethical practices for this critical cadre of staff (11).

## 2. STUDY OBJECTIVES

This study mapped out existing practices in relation to training, support and performance management of fieldworkers in Africa, fieldworkers' and their managers' experiences, and lessons learned. In addition, field managers from international health research institutions were involved in a consultative workshop to identify appropriate ways of addressing the challenges faced by fieldworkers working in research centres in Africa.

## 3. METHODS

The study was planned over two phases and focused on understanding structural and performance management practices and their implications within institutions. In phase one, which was implemented in February 2014, we conducted semi-structured telephone interviews with 32 fieldworker managers and experienced fieldworkers across 18 health research institutions in 14 countries in Africa and the United Kingdom. In phase two, which was implemented in July 2014, we held a two and a half days workshop involving 25 delegates. 18 of these delegates were from the institutions



For peer review only - http://bmjopen.bmj.com/site/about/guidelines.xhtml

that were involved in phase one (i.e. one per institution). The workshop also included the director of training, 3 senior social scientists with long term experience in managing fieldworkers (also Co-PIs of the project), the head of community engagement and 2 senior fieldworkers' supervisor from the KWTRP (12). Fieldworkers were not involved in the second phase.

This paper focuses on the results of phase one (telephone interviews). Issues that were identified during phase one were further discussed in more detail in the consultative workshop in order to build consensus, and identify common recommendations and strategies for addressing the reported challenges. Thus, certain sections of the paper inevitably refer to views shared during the workshop. Results of the consultative workshop have already been published (12).

### 3.1 Patient and public involvement

Patients and the public were not involved in the design and conception of this study

### 3.2 Study site and selection of research institutions and interview participants

This study was led by a team based at the Kenya Medical Research Institute (KEMRI) Wellcome Trust Research Programme (KWTRP) in Kenya. The KWTRP is a long-standing international health research institution with a wide range of existing scientific and capacity building collaborations with other research institutes in Sub-Saharan Africa (see http://kemri-wellcome.org/). During the first phase, our sampling strategy for research institutions, and relevant individuals within these focused on African



For peer review only - http://bmjopen.bmj.com/site/about/guidelines.xhtml

institutions that manage Health and Demographic Surveillance Systems, given their employment of significant numbers of fieldworkers. We identified these institutions from our existing scientific and capacity building linkages, including the INDEPTH Network (http://www.indepth-network.org), an international research network for health and demographic surveillance sites in Africa which encompasses all research centres in Sub-Saharan Africa that have Health Demographic Surveillance Systems.

From the original list of 34 institutions obtained from the KWTP training department and INDEPTH site, we sent an email to the directors, with an information sheet about the study, to invite them to participate in the study. A total of 18 African international research institutions (see table 1) and the MRC-UK and the INDEPTH Network Secretariat in Ghana responded positively. A total of 16 directors did not reply to our email and could not, therefore, determine why they did not respond; thus, the reasons for nonresponse are unknown. These were dropped after three reminders. The 18 African research institutions included a mix of institutions with varied years of operations that employed a diverse number of fieldworkers, engaged in a range of local and international collaborations and specialized in different research programmes/thematic areas. This mixture of characteristics was important in maximizing shared learning and experiences across institutions. In each institution, we wrote to the director and sought permission to interview one senior fieldworker and one field manager. In the letter, we particularly underscored the need to select a manager whose primary responsibility involved working directly with and managing fieldworkers, including offering support supervision, training and other forms of performance management.



For peer review only - http://bmjopen.bmj.com/site/about/guidelines.xhtml

## 3.3 Data collection and analysis

### 3.3.1 *Phase One: Telephone Interviews with managers and fieldworkers*

Out of the 34 directors contacted, 18 sent names of two individuals (1 senior fieldworker and 1 field manager) to be interviewed. We then contacted the proposed individuals and sought their consent to participate in a semi-structured interview, using telephone or Voice Over Internet Protocol (VOIP) communication. Interviews were conducted using an interview guide (Appendix A), that was developed in consultation with members of the study team and pre-tested before being used. In total, the 18 institutions that participated in the study employed a total workforce of 7839 staff, with 2505 (32.0%) being fieldworkers. Table 1 shows all the institutions that participated in the study, marked in dark blue (those that attended the workshop) and light blue (those that did not attend the workshop), with their names anonymized. In total, 7 of the research Institutions that participated in the interviews were from West Africa, 4 were from Southern Africa and 7 were from East Africa. In addition, individuals from one regional research coordination institution from Africa and one UK research funding organization were also interviewed. The Institutions conducted research in various fields including but not limited to Infectious tropical and bacterial diseases (n=9), Population Health and HIV studies (n=7), Social science and health systems research (n=9), Clinical Research/Drug and Vaccine trials (n=5) Epidemiology-Prevention and control of Malaria (n=9), Maternal and Newborn Health (n=6), Non-communicable diseases (n=3) fundamental sciences (n=1), human genetics (n=1), Molecular biology



For peer review only - http://bmjopen.bmj.com/site/about/guidelines.xhtml

(n=1) and Clinical biology and bioinformatics (n=1). All the institutions received funding from external donors, mainly from the USA, United Kingdom (UK) and Canada. The number of staff employed per institution ranged from 45 to 1500 (mean 461), with the number of fieldworkers ranging from 30 to 630 (mean 147).

For peer review only



For peer review only - http://bmjopen.bmj.com/site/about/guidelines.xhtml

# Table 1: Main characteristics of Institutions Involved in the project

| No | Name of Institution | Year Established | The main type of research | Number of Staff | Number of fieldworkers | % of FWs | Source of Funding | Description of training given to Fieldworkers | Description of career progressing system used |
|---|---|---|---|---|---|---|---|---|---|
| 1. | **Research Institution 1-WA.** | 1947 | Infectious tropical diseases | 1104 | 301 | 27.3 | MRC, UK | CORE training is given to all fieldworkers with three-level, including basic, intermediary and advanced. Everybody who gets employed as a fieldworker must go through core training. | Successful completion of fieldworker straining is automatically awarded one salary grade promotion and allows fieldworkers to move through a career progression from A2 A3, B1 B2 B3, C1 C2 C3, D1 D2 D3 and E1 E2 E3. Some fieldworkers move up to C2 after the training because of the change in job description and responsibilities. |
| 2. | **Research Institution 2-SA.** | 1997 | Health and Population, HIV studies, | 800 | 52 | 5.4 | WT, NIH & EGPAF | A mass training is done for 5 weeks: 4 weeks course work and 1 week practical. The test is done to assess skills gained and certificate issued upon passing the exams. Those who pass are put on a waiting list and employed directly to studies when a vacancy arises. | A professional development process involving all staff is available. It is not specific to fieldworkers. |
| 3. | **Research Institution 3-WA.** | 1983 | Anthropology, Demography, Health Economics, Clinical trials | 153 | 40 | 26.1 | NIH and USAID, WHO & EGAD, | A centrally managed training coordinated by a social scientist who takes through the fieldworkers on how to approach the community is usually given | Fieldworkers are usually outsourced. No scheme of service is available |
| 4. | **Research Institution 4-EA.** | 1970 | Infectious diseases, prevention and control | 120 | 40 | 33.3 | NORAD, SIDA, EDCTP, WT, BMGF, WHO, TDR, GLOBVAC, Swiss TPH, | New staff are trained on the specific project and other training such as GCP, Data Management and Ethics. | There is no career ladder but if they improve their educational qualification, they get an opportunity to apply for other higher posts |
| 5. | **Research Institution 5-SA.** | 1996 | Malaria, HIV AIDS, Vaccine Trials, Pneumonia, Bacterial infection | 200 | 100 | 50.0 | AECID, MOH & EDPTP | All fieldworkers undergo project-related training, GCP, Data Management and Ethics upon being employed | None |
| 6. | **Research Institution 6-EA** | 1989 | Immunology, TB, Bacteremia, HIV, Social Science, Health Economics, | 750 | 300 | 40 | WT, IAVI, NIH | Fieldworkers training is centrally coordinated. All new fieldworkers undergo training in communication skills, research ethics, introduction to medical research, GCP and phlebotomy before being allowed to work in the field | A career progression framework for fieldworkers exists, which stipulate the entry point and career progression subject to the attainment of job experience, in-house training as well as external training which could either be self or programme sponsored |
| 7. | **Research Institution 7-EA** | 1998 | Health system research, Maternal and Newborn Health & HIV | 700 | 135 | 19.3 | DFID, INDEPTH, MOH, IDRC, DFID, RF, BMGF, FP7, USAID & CDC-RBM- | Field enumerators who work in the DSS go through a refresher training every six months to share the challenges from the previous round and train them on new issues, discuss the challenges and how to address them we also remind the fieldworkers on the ethical issues for example about their responsibilities | An interviewer and field staff can start as a field interviewer and then be a supervisor and then sometimes field manager. HR has its arrangement but the structure is not formal |
| 8. | **Research Institution 8-EA** | 1996 | Malaria, HIV, Health and Poverty, TB | 800 | 183 | 22.9 | MOH, Swiss Institute, BMG, EU | FWs are given intensive training about how to work in the DSS and the procedures of doing DSS activities. We also train them about ethics and their responsibilities. At the same time, other related issues concerning DSS activities are varied. | The training unit deals with all the training needs and capacity building. So a fieldworker can even be appointed to be a field supervisor or a field manager if they express interest. A structure is available |
| 9. | **Research Institution 9-EA** | 2011 | Health system research, Reproductive health | 250 | 100 | 75.6 | Wellcome Trust | New fieldworkers are paired with more experienced fieldworkers for a given period of time. They are also required to attend mandatory meetings to share their experiences. After every DSS round, a three days' refresher training is organized to recap on what they have done and solve any problems. | Training and capacity building is available for senior research staff but not for fieldworkers |
| 10. | **Research Institution 10-WA.** | 2005 | social scientist and clinical research | 90 | 50 | 50.0 | MOH & LSHTM | Training for field workers is coordinated by the leader of the study team so for every study, we have a team responsible for coordinating the fieldworkers training, who train them about field practices, community entry law and so on. | There is no formal structure for fieldworkers' career development. Only those with a degree and Masters qualification can be promoted to senior positions |
| 11. | **Research Institution 11-WA.** | 1994 | Malaria, TB, HIV, and Maternal and child health | 200 | 100 | 50.0 | LSHTM, The Kwame Nkrumah University of science and technology, University of Ghana | FWs receive training retraining on the forms they use in the field, at the end of the round and the end of the year. Every week, they meet with their supervisors in the field to discuss any emerging issues. | Fieldworkers can be supported to undertake advanced training in nursing or laboratory but there is no guarantee that their position would be reserved when they complete the course. A formal structure is not available |
| 12. | **Research Institution 12-EA.** | 1984 | Malaria, TB, HIV and emerging infection | 1500 | 630 | 42.0 | CDC, EDCTP and other agencies | Fieldworkers are trained on ethics, which entails Good Clinical Practice then they are paired up with more experienced ones and then trained on relevant processes and specific study document, tools and protocol training, general orientation, confidentiality issues and after this, they are left to | There are promotions every year when staff is being evaluated and other conditions which the staff should meet before the supervisor would recommend they get a promotion. No career progression framework for fieldworkers |
| 13. | **Research Institution 13-EA** | 1988 | Maternal and Newborn health | 380 | 105 | 27.6 | MRC UK | All fieldworkers undergo a GCP training and then trained on their specific study protocol | Career progression depends on studies. Different studies have different requirement for a career, some of them may directly be organized by the program for a certain cadre to go for training. We don't have a structure of career progression for fieldworkers |



For peer review only - http://bmjopen.bmj.com/site/about/guidelines.xhtml

| | | | | | | | | | |
|---|---|---|---|---|---|---|---|---|---|
| 14. | **Research Institution 14-SA** | 1995 | HIV and AIDS, Malaria, Non-communicable diseases | 300 | 73 | 24.3 | WT & EU | There is no standard structure. Once employed and taken through HR induction and study protocol, they are ready to go and work. There is no process to build FWs capacity to address practical and ethical challenges FWs face. | No structure available but if there is a new position, a fieldworker who has the appropriate qualification can apply. There is no formal approach to supporting fieldworkers. Sponsorship is competitive against all staff members |
| 15. | **Research Institution 15-SA** | 1979 | TB, HIV, NCD, Clinical and epidemiological research | 184 | 73 | 39.7 | WT, LSHTM, BMGF & EU | Fieldworkers get trained on demographic surveillance its basic outline and principles because most studies are linked to our Continuous Registration System (CRS). They are also trained on HIV counselling and testing and data entry. | No formal career path exists |
| 16. | **Research Institution 16-WA** | 1992 | Vaccine and Drug trials, fundamental sciences, Mosquitoes genetics, Parasites genetics, Drug Resistance (PCR, in Vitro tests…), Malaria Immunology, Malaria and Pregnancy trial | 45 | 30 | 66.7 | NIH, MOH and EDCTP | Fieldworkers are given protocol-specific training and told about their responsibilities and objectives of the study. | Training/Career Development Program is in place to increase the number of qualified scientific and technical personnel capable of conducting independent medical research. This includes post-doctoral training-PhD levels, Parasitology, Cellular Biology, Pharmacology, Immunology; Maters of Sciences: Clinical research, Clinical Pharmacology, Public Health, Parasitology, Entomology, Nutrition. No specific framework for fieldworkers |
| 17. | **Research Institution 17-WA.** | 1998 | Maternal and child health, tropical diseases; malaria, Reproductive Health, Communicable diseases; Non-communicable diseases; human genetics; Health systems research, Social and Behavioural Studies, Poverty and Equity Studies | 263 | 193 | 73 | Ghana Health Service, Rockefeller Foundation, Population Council- USA, USAID | Fieldworkers training depends on the nature of the project, so for instance, if you are doing a survey on the health system, you will be taken through that and the overview of the overall Centre, community engagement and ethics. The project staff and the PI undertake the training | If a junior/frontline staff goes for further studies and has worked for 10 or more years and the field of study is relevant to their line of work, then the Centre, they may be supported. There is no formal structure to support fieldworkers. |
| 18. | **Research Institution 18-WA.** | 2007 | Molecular biology, immunology, clinical biology and bioinformatics | Staff are contracted from other organizations | FWs are contracted through other organization on a need basis then laid off at the end of a study | Depends on the project | National government, UNI-AID, Liverpool University, NIH and Fogarty foundation | New fieldworkers are usually given a Standard Operational Procedure then shown what they are going to do after that, they can be allowed to work in the field. | FWs are contracted through other organization on a need basis then laid off at the end of a study |
| 19. | ***Note: A discussion with MRC-UK and the INDEPTH secretariat in Ghana was also held to crosscheck the information gathered from the interviews held** | | | | | | | | |
| **Total- 7 Research Institution from West Africa, 4 Research Institution from Southern Africa and 7 Research Institution from East Africa and 1 regional research coordination institution from Africa and 1 UK research funding institution** | | | | | | | | | |

view only



For peer review only - http://bmjopen.bmj.com/site/about/guidelines.xhtml

Each telephone interview lasted for approximately one and a half hours. During the interviews, we sought to develop an understanding of current institutional practices and experiences in relation to training, support supervision and performance management of fieldworkers. Interviews with field managers explored issues related to processes of recruiting fieldworkers, the minimum qualification for fieldworkers, and the requirements for professional training as well as internal and external training given to fieldworkers. Additionally, issues around capacity building, support supervision and professional development for fieldworkers were discussed. Interviews with senior fieldworkers focused on their perceptions of the roles they play in research, challenges and mechanisms for addressing these challenges, career development opportunities and other existing support systems. The first author conducted the interviews, which were voice recorded, transcribed and immediately read through for verification and filling of any gaps by the interviewer before analysis.

### 3.3.2 *Phase Two: The workshop with field managers*

A total of 25 managers from 15 of the 18 research centres that took part in the phase one telephone interviews attended the Two and a half days' workshop to further discuss the phase one results. In total, the 15 institutions represented at the workshop employed a total of 6702 staff, with 2136 (31.9%) being fieldworkers. The institutions came from 9 different African countries. To attend the workshop, invitations were sent directly to the individuals who participated in phase one interviews and requested to seek clearance from their line managers before attending the workshop.



For peer review only - http://bmjopen.bmj.com/site/about/guidelines.xhtml

The workshop agenda was developed collaboratively between the study team and an independent Kenyan consultant who acted as the main workshop facilitator, and who had been selected based on his in-depth experience of participatory methods and prior familiarity with research institutions in Africa. Overall, the workshop combined information sharing and large group discussions on day one with a series of participatory consultative and consensus-building activities over the following days. On day one, two sets of presentations were made and discussed in plenary. The first presentation described phase one results, with discussions on the range, strengths and challenges of fieldworkers' employment and support structures across institutions. The second presentation highlighted results from earlier social science research at the KWTRP and other research sites in Africa on the complex nature of fieldwork, including practical, social and ethical challenges that fieldworkers often face as part of their routine work, particularly within low-income communities and households. Discussions that followed the presentations focused on the relevance and importance of these challenges across sites.

## 4. DATA ANALYSIS

Data analysis in this study followed a Framework Analysis approach (13). Transcripts were independently read and discussed in-depth by two members of the study team (FK and VM) to develop an initial coding framework, drawing on the main questions raised during interviews (see Appendix A) and themes that emerged during discussions. FK used Nvivo 10 software to organize data across the transcripts under these codes, adding codes for new issues emerging from the data. Data analysis



For peer review only - http://bmjopen.bmj.com/site/about/guidelines.xhtml

charts were developed by FK and VM to account for the codes under themes by the participants, and support interpretation of patterns within the responses, drawing on the social science and research ethics literature.

Preliminary analysis of the telephone survey data was used to inform about the type and range of fieldworkers' support supervision and training practices as well as performance management strategies. The themes generated from this initial analysis were explored further and discussed in detail during the consultative workshop, with a view for consensus building and checking for similarities and variations across different research institutions. Emerging new themes were also discussed and explored using a combination of qualitative participatory approaches, including large and small group discussions, round table and moderated discussions.

Moderators were careful to discuss ideas in detail without influencing the direction and outcome of the discussion. During the analysis, we compared and contrasted the fieldworkers' and field managers' views based on specific themes. Through this, we realized there were issues that fieldworkers and field managers shared the same views and others that they had different views. In this paper, we have tried to present these similarities and contracts together, rather than separately. We believe this will be much easier for our readers to follow than it would have been if we separated the views of fieldworkers from those of the field managers. Finally, although our participants are classified as fieldworkers and field managers, it is important to note that questions in the interview guides that were used to interview them were very



For peer review only - http://bmjopen.bmj.com/site/about/guidelines.xhtml

similar. Despite the similarity of the interview guide used, the field managers interviews were considerably longer, allowing for a more in-depth exploration of issues. This makes it logical to analyze and present these data together as shown in the next section

## 5. RESULTS

In this section, we describe and discuss results from the telephone survey and where applicable, the highlights of the workshop, across three inter-related themes that were explored or emerged as important across this study. These themes are A) The current institutional structures and systems that support fieldworkers across the institutions involved in the study; B) Participants' perceptions of work-related challenges for fieldworkers and their relationship to support structures and; C) Views on the ways in which challenges for fieldworkers might be addressed, including agreement on institutional and wider recommendations.

### 5.1 Current Institutional structures and systems for fieldworkers

Across interviews and the subsequent workshop, it became clear that there is a large amount of diversity in the names, tasks and institutional structures that govern and support the way that fieldworkers operate in the institutions involved. For example, during interviews, senior fieldworkers described the existence of many different tasks and titles, including fieldworker, field enumerator, field officer, tracer, community technician and field assistant, which were often used interchangeably, within and across different centres. Similarly, the way that fieldworkers were recruited, contracted, trained and supported in their work was highly variable, between projects



For peer review only - http://bmjopen.bmj.com/site/about/guidelines.xhtml

within an institution and between institutions. Table 1 describes these parameters across the research institutions involved.

As Table 1 shows, some form of in-house fieldworkers training was common across the institutions, with training mainly focused on the skills needed to undertake study-specific tasks, such as using particular data collection tools. In some cases, training in Good Clinical Practice (GCP) was also offered, particularly to support fieldworkers undertaking informed consent processes. Often, such training was generally the responsibility of individual study teams within institutions. 4 out of the 15 institutions (27%) confirmed having a centrally coordinated training approach, 3 of which covered a more comprehensive set of topics including data collection skills, communication skills, informed consent processes, GCP and research ethics more generally. The other sites described conducting some kind of *ad hoc* training based on a specific study requirement or had no training plans for fieldworkers.

One area of fieldworker performance management which was rarely discussed and generally under-recognized across institutions was the concept of fieldworkers' professional career progression or continuing professional development. While such measures were largely routine for other cadres of research staff within institutions, they had not been applied to fieldworkers. Many felt this would be an important step towards recognizing the important role fieldworkers play, and in motivating them to maintain a high professional standard of work, but challenges were seen in sustaining

For peer review only



For peer review only - http://bmjopen.bmj.com/site/about/guidelines.xhtml

such an initiative given the typically short-term contracts offered to fieldworkers, based on similarly short research funding cycles.

> *"It is good to establish a professional development scheme for fieldworkers. However, such a system may not be sustainable, given the dynamic employment terms of fieldworkers and researcher in general…So although it is a good concept, it may be difficult to implement it [the scheme] within the context of research that relies on funding that is not always guaranteed"* (FWM, SA[1])

## 5.2 Participants' perceptions of work-related challenges for fieldworkers and their relationship to support structures

Across the following paragraphs, we describe the forms of challenges that fieldworkers and those working closely with them described during interviews, and that were taken forward for discussion at the workshop. In describing these, we highlight the types of institutional support that influence these challenges, including structural influences from human resource management strategies and research funding cycles, and more *ad hoc* often individual efforts to manage complex challenges on the ground.

### *5.2.1 Using different 'labels' for fieldworkers*

Discussions during interviews and at the workshop drew out differences in opinions on the importance of the 'labels' that were used to describe the work we describe here as

---

*[1] First three letters of the initials on the quote stand for the position of the interviewee i.e Senior Field Worker (SFW) and Field Worker Manager (FWM); and the last two letters represent the country eg Kenya (KN), South Africa (SA), Ghana (GN) etc*



For peer review only - http://bmjopen.bmj.com/site/about/guidelines.xhtml

'fieldwork'. At a most pragmatic level, some fieldworkers felt that the most important issue was having a job, rather than being concerned about the title that was associated with that job. Practically, this attitude seems to hint at potentially problematic power imbalances for fieldworkers in relation to the institutions in which they were employed. Other fieldworkers felt that using different 'labels', particularly within one institution, could generate confusion about roles and lead to employment conditions being less transparent:

> *"It [roles taken by field workers] is not different. These are the organisation's terms…community technician, field worker, follow up staff, field technician…I mean it is just the same thing…it's just their [the organisation] way of making us look different, but we are the same".* (SFW, KE)

Many field managers recognised the phenomenon of multiple labels as being used for slightly different forms of 'fieldwork'. There was, however, an initial divergence of opinion about the value of harmonising job titles. Some felt that this variation underlined important, even if small differences, in fieldworkers' current roles and prior experience or qualifications, which might not be obvious to all team members. Others felt there was little need for multiple titles, given the potential for confusion and conflict this might present. As will be described in the later section on recommendations, developing clear and strategic approaches to fieldworkers' roles within an institution emerged as an important feature of good practice for fieldworkers' employment. For many, harmonizing these structures and processes across institutions was also seen as valuable. Field managers felt harmonizing fieldworker employment terms, training and skills would promote cross-study and institution transfer of fieldworkers and



For peer review only - http://bmjopen.bmj.com/site/about/guidelines.xhtml

address the challenge associated with a high turnover of fieldworkers due to short-term contracts.

### *5.2.2 Seeing fieldwork as a short-term activity*

A core tension that was strongly expressed in interviews and the workshop was a general lack of recognition for the work that fieldworkers undertake within research institutions. Fieldworkers themselves saw their work as important, and as fundamentally contributing to answering important research questions, promoting the reputation of institutions, careers of scientists and other cadres of research staff. There was, however, some frustration and perceived unfairness around the lack of opportunities for fieldworkers to progress within their area of work, or achieve recognition over sometimes periods of many years working in this role. Experiences were shared in which scientists who joined research centres were able to move ahead to higher positions through focused capacity-building strategies, while fieldworkers' positions remained static. Some fieldworkers described working for an institution for more than ten years without more than basic training or promotion. Perhaps not surprising, given the way that 'fieldwork' has traditionally been viewed within research institutions (that is, as short-term and relatively non-technical work), the institutions involved in this study had not developed strategic approaches for fieldworkers' career development.

In response to these frustrations, some fieldworkers had taken up self-funded part-time training courses outside their existing jobs in the hope that this would allow them

For peer review only

to progress within the institution. This signified the importance of how fieldworkers valued education and training as an important element of their career advancement. In practice, however, institutions did not have structures that would allow fieldworkers to move into roles in which such new skills could be used. Against this background, most fieldworkers reported feeling demotivated, including a feeling of low self-esteem and disillusionment:

> *"Once you are employed as a fieldworker, you will always be a fieldworker* [regardless of your qualifications]"*. (SFW, GN)*

While some field managers supported this view, some argued that career development was a matter of personal motivation and determination. They explained how they [managers] had moved through the career ladder to become field managers, often starting their careers as junior fieldworkers and gaining extensive fieldwork experience along with their career progression to their current positions, with the same limited training and support. Most managers had therefore been part of the system for a long time and were able to share their views both from the perspective of fieldworkers as well as field managers hence extending the spectrum of experiences adduced and provide deeper insights to the issues that were raised. Given these challenges, which workshop participants saw as fundamentally unfair and as potentially importantly undermining the quality of research at its roots, participants agreed that fieldworkers should have greater recognition for the role they play within research institutions. They also agreed that it was important for institutions to support some form of career progression, for example, within a specialised category of staff supporting fieldwork. Providing fieldworkers with generic knowledge and skills on core research areas as



For peer review only - http://bmjopen.bmj.com/site/about/guidelines.xhtml

part of such a scheme would enable them to transfer between studies at the end of a funding cycle. This would enable research centres to retain experienced fieldworkers, a policy that was thought likely to promote high scientific and ethical standards; and give fieldworkers more stability, skills and recognition.

For peer review only



For peer review only - http://bmjopen.bmj.com/site/about/guidelines.xhtml

### 5.2.3 Responding to complex challenges on the ground

In both the telephone interviews and the subsequent workshop, fieldworkers and managers described facing often very difficult challenges that seem to be implicit in the nature of much fieldwork. Some of these were practical, including the physical burdens of fieldwork, such as the experience of long working days with long distances to cover on foot, risks of injury through dog or snake bites, and physical assault in less secure settings. Many also described emotional burdens including anxiety, fear, sadness and sometimes helplessness when interacting with individuals and families facing severe social and economic hardship. Within the community, fieldworkers said they were perceived as ‘fellow community members’ and, at the same time, the ‘face’ of the institutions that employ them, generating expectations of support that were beyond their mandates from the community members they visited (14).

These challenges have been well described in the literature on global health ethics, including from social science research at KWTRP, and our results in this study underline the existence of these important challenges (1, 15-18). The fact that our study draws on experiences from 18 major research centres in Africa and found similar challenges is an indication of how common and important those challenges are. Within this literature, there is increasing recognition of the moral work that fieldworkers are involved in during their everyday work (3, 19-21) which can directly impact the science and ethics of the studies they work for (5, 22). For example, a key issue in the literature concerns the negotiation of informed consent processes, where fieldworkers balance perceived pressures to recruit participants according to targets set by



For peer review only - http://bmjopen.bmj.com/site/about/guidelines.xhtml

managers and the extended period that may be needed to explain complex and often unfamiliar topics within consent forms to household members. Besides, community members may feel that by agreeing to participate in studies, they will develop some leverage to access greater institutional support, including through their relationships with fieldworkers (4, 5, 23-25). Similar ethical challenges experienced by fieldworkers have been reported in high-income settings, including the United States of America (USA), with a specific focus on how fieldworkers' and study coordinators' practices can affect the protection of research participants (26). Although it could be argued that these settings are contextually different, it would be interesting to examine why fieldworkers in High-Income Countries (HIC) experience the same ethical challenges as those in Africa, despite them being better trained, educated and resourced than their counterparts. (2, 26)

A particularly difficult situation that fieldworkers involved in routine Demographic Surveillance System (DSS) activities in this study described was the need to directly interact with bereaved families and seek information about deceased relatives as part of conducting verbal autopsies (interviews held with close family members of a recently deceased person to try to establish the cause of death)(27, 28). Such emotionally challenging interactions generated distress and worry for fieldworkers and could chronically undermine performance, leading to ‘burnout’:

> *“Yeah but you see what, after maybe going through these challenges sometimes they affect you or they affect your output” [SFW, SA]*
>
> *….”I felt bad, and you know the following day I was to go back to work, and there is no supportive counselling or something like that… So as fieldworkers*



For peer review only - http://bmjopen.bmj.com/site/about/guidelines.xhtml

*sometimes we just burn out and sometimes with this kind of trauma, it becomes difficult…we go through a lot*' [SFW, KE]

During workshop discussions about support mechanisms to help fieldworkers address these emotional challenges, field managers were not able to identify existing strategies; as was also reported by fieldworkers during the telephone interviews. Some managers described generally relying on fieldworkers' judgements in handling these difficult situations.

*"It is difficult to anticipate what challenges fieldworkers are likely to face…and even if they were to share them [the challenges] some of them are too complex to resolve. It is, therefore, easier to just let them [fieldworkers] solve them spontaneously and trust that they use enough wisdom to do the right thing"* [FWM, KE]

Experiences shared by fieldworkers revealed that the issues were usually quite complex and intertwined with cultural, structural and emotional sensitivities associated with the need to balance community and cultural expectation against the policies and guidelines of research institutions. Thus, fieldworkers were often expected to strike a delicate balance, sometimes without the knowledge of how acceptable their actions were. Despite these actions having the potential to directly affect the scientific and ethical standards of data that fieldworkers collect, structural support and referral systems were reportedly not available. This was well captured by one senior fieldworker who said.

For peer review only

"…. *sometimes, you get that huruma [pity], you go into your pocket and give out something…if you have a human heart, sometimes some situations are touching"* [SFW, SA].

During interviews and workshop discussions, there were no accounts of existing internal or external institutional mechanisms to support fieldworkers to specifically deal with these types of emotional challenges. Although effective support supervision and sharing these challenges during team meeting was identified as a potential avenue to identify strategies to deal with these challenges, the approaches used to supervise fieldworkers were noted to be significantly different across institutions, and not centrally coordinated within institutions, making it difficult to reflect on their effectiveness. Workshop participants therefore generally felt there was a gap in fieldworkers' support structures that should be addressed. These challenges were seen as occurring across all the institutions involved in the study.

### 5.3 Ways of strengthening institutional support

Based on the outputs of discussions described so far, two core institutional fieldworker support strategies were raised, discussed and agreed upon over the course of the workshop. The first concerned adapting the *content of skill-building initiatives* to support fieldworkers to manage their interactions with community members and household members in ways that could support good scientific and ethical practice, seen from multiple perspectives. The second strategic support mechanism addressed the wider question of how professional support within institutions could support fieldworkers more *structurally* towards these aims, including aspects of performance



For peer review only - http://bmjopen.bmj.com/site/about/guidelines.xhtml

management that commonly exist for other groups of research staff. Workshop participants agreed that ‘performance management’ in this respect would include aspects of recruitment, training, supervision, support, assessment and career or professional development. Specifically, delegates emphasized the need to strengthen fieldworkers’ interpersonal skills training to ensure they are well equipped to deal with cross-cutting issues around communication, community engagement, building trust and respect, which are fundamental in consenting and quality data collection. Fieldworkers themselves believed that more support would improve their skills, and accordingly the quality of the data they collect. The need to develop a harmonized training curriculum, with core training modules across Africa was identified.

In this study, we noted a mismatch related to lack of appropriate training for fieldworkers; fieldworkers’ limited level of education and the complexity of the tasks they are expected to undertake, including but not limited to translating study information to study participants, understanding institutional and international ethical guidelines and applying these when interacting with study participants. These results support the need for research institutions in Africa to invest in training and building the capacity of fieldworkers, to enhance the standards of the work fieldworkers undertake and provide optimum support for research.

### 5.4 Agreement on recommendations

Given the issues discussed in the preceding paragraphs around institutional fieldworkers’ support structures, participants at the workshop agreed there was an



For peer review only - http://bmjopen.bmj.com/site/about/guidelines.xhtml

urgent need to introduce more standardized and comprehensive approaches, focused on important areas that affect fieldworkers' performance and practices, including career progression, training/capacity building and management of emotional and ethical challenges. It was felt that strategies could build on the positive experiences of research institutions that had already begun to institutionalize such approaches. Participants recommended a pragmatic approach, aimed at empowering and building the knowledge and skills of fieldworkers, including processes to support continuous learning through the regular reflection of the practical and ethical issues they face in everyday experience. In this way, an important element of training and support supervision was described as enhancing skills to recognise and respond to these ethical issues. The specific recommendations made are described and discussed in the following paragraphs.

### *5.4.1 Creating space for experience sharing and problem-solving*

One challenge identified during the survey was lack of psychological support after experiencing difficult and sometimes traumatic experiences, such as some interviewees breaking down during verbal-autopsy interviews, losing a study participant when close relationships had been built and even being physically attacked by emotional/aggrieved participants during interviews. Long working days with long distances to cover on foot, risks of injury through dog or snake bites, and physical assault in less secure settings were also said to be emotionally overburdening to fieldworkers. Documented evidence shows that stressful and traumatic experiences can alter the way people view themselves, the world around them, and alter how they



For peer review only - http://bmjopen.bmj.com/site/about/guidelines.xhtml

process information and the way they behave and respond to the environment around them(29). A strategy described in the literature to address this challenge is the use of regular de-brief sessions, where fieldworkers are given opportunities to share experiences and consider the best approach to address challenges. During the workshop, participants recommended that field managers should create such opportunities for fieldworkers to talk about distressing or worrying issues encountered at work, through forms of feedback and reflection. This approach also requires a support supervision mechanism that is sensitive to the complex nature of the experiences fieldworkers face. This supports True's (2011) arguments that having access to colleagues to discuss issues and having effective support supervision acted as a buffering factor for research front-line staff who did not engage in ethical misconduct, despite facing the same pressures to meet recruitment goals and other stressors of conducting community-based research (30). Combining different ways of knowing and learning will enable fieldworkers to standardize the way they handle practical and ethical issues they encounter, even where these present as dilemmas with limited information on the best course of action. At the same time, in some cases, more traditional approaches to managing emotional distress, such as staff counselling, might also be needed.

### *5.4.2 Increasing attention on soft skills for fieldworkers*

As reported by both fieldworkers and field managers, advancement has been made in the area of technical training for research staff, including fieldworkers. Good Clinical Practice, Health and Safety, Good Clinical Laboratory Practice (GCLP) training have



For peer review only - http://bmjopen.bmj.com/site/about/guidelines.xhtml

become a standard approach for clinical study site preparation. While this training enables studies to adhere to international GCP/GCLP standards, they rarely equip fieldworkers with the necessary skills to enable them to deal with practical local challenges they face on the ground. There is, therefore, a need for fieldworkers to undergo more generic interpersonal skills training, such as communication skills, basic psychology and counselling and community engagement technics to enable them to appreciate the practical local situations and develop skills to deal with the ground challenges.

### *5.4.3 Having career development guidelines that are responsive to fieldworkers*

In a previous commentary by Kombe et al (2015), that reported the views of field managers who attended a consultative workshop as part of this study, important recommendations made by the managers were presented, including the need to i) increase institutional recognition of fieldworker's roles and the need for systematic and comprehensive capacity building to ensure adequate resources are allocated and that capacity building activities are well described in research proposals and grant applications; ii) develop common areas of a core curriculum for fieldworkers capacity building to enhance quality of training processes and build their knowledge in basic biology, research approaches and methods, research ethics and research regulatory frameworks as well as data collection and documentation; respectful communication, and being aware of and managing ethical challenges and issues in practice; and iii) increase emphasis on fieldworkers' career development including developing regionally accredited training to support fieldworkers' professional development to



For peer review only - http://bmjopen.bmj.com/site/about/guidelines.xhtml

increase individual motivation and enhance capacity for employment across different research organizations (12). These recommendations should be considered in order to address the challenges presented in this paper and enhance the overall quality and integrity of research in Africa. Similar strategies have been proposed by True et al, to address challenges faced by community members employed as research staff in the USA (2)

## 6. DISCUSSION

The important role fieldworkers play in health research can be enhanced if fieldworkers are given adequate support. The challenges they face require a combination of knowledge, skills, attitude change and ethical values that can be enhanced through structured institutional support and instilling a sense of professional responsibility and moral values to fieldworkers; through ongoing support supervision, soft skills training and creating space for consultative discussions and experience sharing.

In this study, fieldworkers were reported to provide important support for research. From the institutions that participated in the study, fieldworkers were employed to undertake diverse tasks and were given a range of different titles. All participants that were interviewed acknowledged the fact that the tasks undertaken by fieldworkers were critical in supporting health research. On the other hand, Fieldworkers reported experiencing a myriad of challenges, a fact that was supported by the fieldworker managers interviewed, and strongly deliberated upon during the workshop, during the second phase of this study.



For peer review only - http://bmjopen.bmj.com/site/about/guidelines.xhtml

Despite the above-perceived importance of the tasks fieldworkers undertake in supporting health research, there were no centrally and institutionally coordinated systems or structures that were reported to have been put in place to provide the much-needed support for fieldworkers in most of the institutions involved in the study. Fieldworkers coordination and support were largely left in the hands of study-specific field managers and supervisors, who often focused on the outputs of the specific studies they coordinated, with much less focus on generic support and supervision systems. Institutional systems to support fieldworkers to undertake their tasks effectively were reported to be limited or often unavailable. Furthermore, in spite of the myriad of challenges fieldworkers reported to experience in their day to day activities, which were confirmed by their field managers during the telephone interviews and the consultative workshop, no institutional systems were reported to be in place to support fieldworkers in addressing such challenges. Fieldworkers were, therefore, essentially expected to use their discretion, cultural competence and moral reasoning to navigate through the many practical and ethical challenges they faced. Given the limited support, education and ethics knowledge and competence fieldworkers possessed, this situation, points to serious implications on the quality of the data fieldworkers collect, their ethical practice and the overall integrity of the health research enterprise fieldworkers are expected to support.

This study interviewed participants via telephone. Although this could not have necessarily affected the quality of the data collected, important qualitative interview



For peer review only - http://bmjopen.bmj.com/site/about/guidelines.xhtml

dynamics, which can provide important insights into the data analysis process could not be identified and considered. However, the triangulation of different study approaches, including the consultative workshop with field managers, provided an important approach for validating the data collected and strengthening the study design.

The results from this study underscore the need to establish and strengthen institutional support for health research fieldworkers. Although a lot of studies have been done to understand the moral and ethical challenges and dilemmas faced by fieldworkers, very little has been done to explore which fieldworkers' institutional support systems exist and what implications such systems have on fieldworkers' scientific and ethical practices. There is, therefore, a need for further research, to develop a deeper understanding of this area. This study has highlighted important gaps related to the management and coordination of fieldworkers, that require further assessment and strategic consideration. Furthermore, the strategic solutions and recommendations identified in this study call for the need for health research institutions in Africa and funders of health research to strongly consider developing institutional systems and structures to facilitate centralized coordination and management of fieldworkers by health research institutions in Africa. As stated by Alexander and Richman, strengthening the capacity of fieldworkers has the potential to increase the protection of research participants, enhance research integrity and promote the collection of valid data (31). Efforts to empower fieldworkers to make them better able to deal with these challenges should therefore continue.



For peer review only - http://bmjopen.bmj.com/site/about/guidelines.xhtml

This was an exploratory study hence the results are not generalizable. However, they do offer important insights on fieldworkers and field managers' views and experiences of fieldworkers' performance managing practices for institutions that come from similar settings as those that participated in this study. Although some of the proposed approaches such as interpersonal skills training, centralized coordination of fieldworkers training, fieldworkers' scheme of service and regular discussions to address fieldwork challenges have been ongoing in institutions such as the KWRTP, in Kenya, lack of documented evidence makes it difficult to say how effective and sustainable such approaches are. More research, therefore, needs to be done to explore the feasibility of the proposed strategies and generalizability of these study results within a wider context in Africa. Finally, the proposed solutions may not solve all the challenges fieldworkers face, but they are certainly an important step towards improving the scientific and ethical standards of health research output from the African continent. The value of this study is not that every research centre in Africa can identify with every issue raised here, this would be difficult due to the variety of research centres in the continent, rather, we hope that by raising the issues highlighted here, even those centres that were not involved in the study will be provoked to examine their fieldworkers support system and see if any issues raised here apply to their setting. Furthermore, empirical studies should be done to identify which approaches are most effective in enabling research fieldworkers to deal with the myriad of challenges they face. Such research will go a long way in improving the scientific and ethical standards research, not only in Africa but internationally, given the critical global role of Africa in research.



For peer review only - http://bmjopen.bmj.com/site/about/guidelines.xhtml

## 7. DATA AVAILABILITY STATEMENT

Data are available in the form of transcripts that are currently archived in password-protected computers. All the transcripts are anonymized, to avoid identification of the participants who were interviewed. Permission was obtained to use the data for purposes of this study only. No permission was granted to use the data for future research.

For peer review only



For peer review only - http://bmjopen.bmj.com/site/about/guidelines.xhtml

## 8. REFERENCES

1. Kamuya DM, Theobald SJ, Munywoki PK, Koech D, Geissler WP, Molyneux SC. Evolving friendships and shifting ethical dilemmas: fieldworkers' experiences in a short term community based study in Kenya. Dev World Bioeth. 2013;13(1):1-9.

2. True G, Alexander LB, Fisher CB. Supporting the role of community members employed as research staff: Perspectives of community researchers working in addiction research. Social Science & Medicine. 2017;187:67-75.

3. Kamuya DM, Theobald SJ, Marsh V, Parker M, Geissler WP, Molyneux SC. “The one who chases you away does not tell you go”: silent refusals and complex power relations in research consent processes in Coastal Kenya. PloS one. 2015;10(5):e0126671.

4. Chantler T, Otewa F, Onyango P, Okoth B, Odhiambo F, Parker M, et al. Ethical Challenges that Arise at the Community Interface of Health R esearch: Village R eporters’ Experiences in Western K enya. Developing world bioethics. 2013;13(1):30-7.

5. Kingori P. Experiencing everyday ethics in context: Frontline data collectors perspectives and practices of bioethics. Social science & medicine. 2013;98:361-70.

6. Madiega PA, Jones G, Prince RJ, Geissler PW. ‘She's my sister-in-law, my visitor, my friend’–challenges of staff identity in home follow-up in an HIV trial in Western Kenya. Developing world bioethics. 2013;13(1):21-9.

7. Marshall PA, Marshall PL. Ethical challenges in study design and informed consent for health research in resource-poor settings: World Health Organization; 2007.

8. Richman KA, Alexander LB, True G. Proximity, ethical dilemmas, and community research workers. AJOB Primary Research. 2012;3(4):19-29.

9. Lansang MA, Dennis R. Building capacity in health research in the developing world. Bulletin of the World Health Organization. 2004;82(10):764-70.

10. Glickman SW, McHutchison JG, Peterson ED, Cairns CB, Harrington RA, Califf RM, et al. Ethical and scientific implications of the globalization of clinical research. New England Journal of Medicine. 2009;360(8):816-23.

11. Participants of an International Workshop in Kenya on the Role of Frontline Staff in Biomedical Research J, Kombe F. Enhancing quality and integrity in biomedical research in Africa: an international call for greater focus, investment and

For peer review only

standardisation in capacity strengthening for frontline staff. BMC Med Ethics. 2015;16(1):77.

12. Kombe F. Enhancing quality and integrity in biomedical research in Africa: an international call for greater focus, investment and standardisation in capacity strengthening for frontline staff. BMC medical ethics. 2015;16(1):77.

13. Srivastava A, Thomson SB. Framework analysis: a qualitative methodology for applied policy research. 2009.

14. Molyneux S, Kamuya D, Madiega PA, Chantler T, Angwenyi V, Geissler PW. Field workers at the interface. Developing world bioethics. 2013;13(1):ii.

15. Kingori P. Experiencing everyday ethics in context: frontline data collectors perspectives and practices of bioethics. Social science & medicine. 2013;98:361-70.

16. Molyneux S, Kamuya D, Madiega PA, Chantler T, Angwenyi V, Geissler PW. Field workers at the interface. Dev World Bioeth. 2013;13(1):ii-iv.

17. Chantler T, Otewa F, Onyango P, Okoth B, Odhiambo F, Parker M, et al. Ethical challenges that arise at the community interface of health research: village reporters' experiences in Western Kenya. Dev World Bioeth. 2013;13(1):30-7.

18. Angwenyi V, Kamuya D, Mwachiro D, Marsh V, Njuguna P, Molyneux S. Working with Community Health Workers as 'volunteers' in a vaccine trial: practical and ethical experiences and implications. Dev World Bioeth. 2013;13(1):38-47.

19. Kamuya DM, Theobald SJ, Munywoki PK, Koech D, Geissler WP, Molyneux SC. Evolving Friendships and Shifting Ethical Dilemmas: Fieldworkers' Experiences in a Short Term Community Based Study in K enya. Developing world bioethics. 2013;13(1):1-9.

20. Wax ML. On fieldworkers and those exposed to fieldwork: Federal regulations and moral issues. Human Organization. 1977;36(3):321.

21. Zinn MB. Field research in minority communities: Ethical, methodological and political observations by an insider. Social problems. 1979;27(2):209-19.

22. Kombe F, Anunobi EN, Tshifugula NP, Wassenaar D, Njadingwe D, Mwalukore S, et al. Promoting Research Integrity in A frica: An African Voice of Concern on Research Misconduct and the Way Forward. Developing world bioethics. 2014;14(3):158-66.

23. Molyneux S, Kamuya D, Marsh V. Community members employed on research projects face crucial, often under-recognized, ethical dilemmas. The American Journal of Bioethics. 2010;10(3):24-6.



For peer review only - http://bmjopen.bmj.com/site/about/guidelines.xhtml

24. Mosavel M, Ahmed R, Daniels D, Simon C. Community researchers conducting health disparities research: Ethical and other insights from fieldwork journaling. Social science & medicine. 2011;73(1):145-52.

25. Simon C, Mosavel M. Community members as recruiters of human subjects: Ethical considerations. The American Journal of Bioethics. 2010;10(3):3-11.

26. Davis AM, Hull SC, Grady C, Wilfond BS, Henderson GE. The invisible hand in clinical research: The study coordinator's critical role in human subjects protection. The Journal of Law, Medicine & Ethics. 2002;30(3):411-9.

27. Zahr CA, Pinchuk M. Verbal autopsy standards: ascertaining and attributing the cause of death: World Health Organization; 2007.

28. Fottrell E, Byass P. Verbal autopsy: methods in transition. Epidemiologic reviews. 2010;32(1):38-55.

29. Steele W. Trauma's Impact on Learning. Trauma and Loss: Research and Interventions. 2002.

30. True G, Alexander LB, Richman KA. Misbehaviors of front-line research personnel and the integrity of community-based research. J Empir Res Hum Res Ethics. 2011;6(2):3-12.

31. Alexander LB, Richman KA. Ethical Dilemmas in Evaluations Using Indigenous Research Workers. American Journal of Evaluation. 2008;29(1):73-85.

For peer review only



For peer review only - http://bmjopen.bmj.com/site/about/guidelines.xhtml

## 9. CONTRIBUTOR AND GUARANTOR INFORMATION

All authors were involved in conceptualizing the idea and contributed in writing the manuscript. Mr Francis Kombe (MPH), a male senior community facilitator with a wealth of experience in health research ethics, community engagement and communication skills, who worked as a fieldworker himself, before taking a managerial role at KWTRP, contributed to the planning, conduct, and reporting of the work described in the article and is responsible for the overall content. Prof Vicki Marsh (PhD), a female senior social scientist at KWTRP and Oxford University and Ms Dorothy Ikamba (Msc), a female career development officer at KWTRP, and career counsellor, assisted with reviewing the transcripts and the development of themes and sub-themes during the analysis. Prof Sassy Molyneux (PhD), a female senior social scientist at KWTRP and Oxford University, Dr Dorcas Kamuya (PhD), a female social scientist at KWTRP and Wellcome Trust Society & Ethics Fellow, and Dr Samson Kinyanju, a male immunologist and head of training at the KWTRP, contributed in designing the initial research ideas, reviewed the data collected and were involved in drafting the initial manuscript and edited subsequent versions. All authors reviewed and approved the final manuscript for publication. Francis Kombe accepts full responsibility for the work and the conduct of the study. He had full access to the data and controlled the decision to publish. Francis Kombe attests that all listed authors meet authorship criteria and that no others meeting the criteria have been omitted.

## 10. COPYRIGHT/LICENSE FOR PUBLICATION

Francis Kombe has the right to grant on behalf of all authors and does grant on behalf of all authors, a worldwide license to the publishers and its licensees in perpetuity, in all forms, formats and media (whether known now or created in the future), to i) publish, reproduce, distribute, display and store the contribution, ii) translate the Contribution into other languages, create adaptations, reprints, include within collections and create summaries, extracts and/or, abstracts of the contribution, iii) create any other derivative work(s) based on the contribution, iv) to exploit all subsidiary rights in the contribution, v) the inclusion of electronic links from the contribution to third party material where-ever it may be located; and, vi) license any third party to do any or all of the above

For peer review only

## 11. TRANSPARENCY STATEMENT

Francis Kombe affirms that the manuscript is an honest, accurate, and transparent account of the study being reported; that no important aspects of the study have been omitted; and that any discrepancies from the study as originally planned have been explained.

## 12. COMPETING INTERESTS

The views on this paper are the authors own viewpoints and do not represent the position of their organizations. The authors declare no conflict of interests.
The authors declared that they have no competing interests.

## 13. ETHICAL APPROVAL

The paper is published with the permission of the centre director, KEMRI. All the participant who participate in the telephone survey as well as those who attended the consultative workshop gave individual to consent to participate in the study and the workshop and permitted the publication of this manuscript. Permission to conduct this study was granted by a review conducted by the Centre for Geographic Medicine Research, Coast (CGMRC)- Centre Specific Committee and final Ethical approval was given by the Research and Scientific Review Unit (SERU) at the Kenya Medical Research Institute (KEMRI).

For peer review only



For peer review only - http://bmjopen.bmj.com/site/about/guidelines.xhtml

**DISCUSSION GUIDE FOR RESEARCH INSTITUTIONS WORKING WITH FIELDWORKERS (FWs) IN SSA**

**TITLE: STRENGTHENING TRAINING OF FRONT LINE (FWs) STAFF AS A MEANS TO ENHANCE COMMUNITY ENGAGEMENT**

**INFORMATION SHEET FOR MANAGERS AND SENIOR FRONTLINE STAFF**

## What is this survey all about?

This survey will be conducted through telephone/ teleconference discussion. One front line staff manager (people who have a direct role of providing support supervision to frontline staff) and one senior frontline staff member from a total of 20 health research institutions in Sub-Saharan Africa will be invited to participate in the discussion.

The aim of the discussion is to identify practical and ethical challenges experienced by frontline staff in their day to day activities and the available support supervision, performance management and Continuing Professional Development (CPD) practices within these institutions. Later, representatives from a few of the 20 institutions will be invited to a consultative workshop to follow up and discuss these issues in detail. The report generated from the survey and workshop will be shared with all institutes for their own use. In the long term, the report could be used to inform further discussions on the potential of developing a generic curriculum for frontline staff training.

## What is expected of you if you take part in this discussion?

If you agree to take part in this discussion, we will request you to select a date and time when you would prefer the moderator to call and have the discussion with you. We will then call and ask a few questions with regards to your perceptions of the roles of frontline staff and the institutional support supervision, monitoring and evaluation and general CPD practices. These questions are shown on the attached discussion guide for your attention. The discussion is expected to take a maximum of 30 minutes.

Participation in this interview is voluntary. While it is important to answer all the questions, feel free to skip any question that you do not feel comfortable to answer and the moderator will continue asking the next question. You can also decide to terminate the discussion at any time if you decide not to continue with the discussion for any reason.

## Is there any individual benefit for taking part in this discussion?

There is no individual benefit in taking part in this discussion. However, by sharing your experiences in working with frontline staff, you will assist in contributing to knowledge that could improve the way frontline staff deal with their practical and ethical challenges as well as their support supervision, performance and general CPD management practices in future.

## If you have any question

If you have any question about this survey, please contact any of the following people through the email addresses provided below


| | | |
|---|---|---|
| Francis Kombe<br>KWTRP<br>P.O BOX 230, KILIFI<br>Fkombe@kemri-wellcome.org | Sam Kinyanjui<br>KWTRP<br>P.O BOX 230, KILIFI<br>Skinyajui@kemri-wellcome.org | Dorcas Kamuya<br>KWTRP/ Ethox-Nuffield Department of Population Science, Oxford University<br>P.O BOX 230, KILIFI<br>Dkamuya@kemri-wellcome.org |




For peer review only

**DISCUSSION GUIDE FOR RESEARCH INSTITUTIONS WORKING WITH FIELDWORKERS (FWs) IN SSA**

**TITLE: STRENGTHENING TRAINING OF FRONT LINE (FWs) STAFF AS A MEANS TO ENHANCE COMMUNITY ENGAGEMENT**

## SECTION A: Institutional Background

1. Name of organization ______________________________
2. Vision ______________________________
3. Mission ______________________________
4. Motto ______________________________
5. Year organization was established [__][__][__] [__]
6. Source of main funding for the organization ______________________________
7. Other funding agencies and collaborators ______________________________
8. Types of research conducted at the organization: ______________________________

## SECTION B: Frontline staff Managers' Perspective[1]

*This section explores managers' perceptions of the roles of and practical and ethical challenges faced by interface staff when discharging their duties and the institutional support supervision, performance management and Continuing Professional Development (CPD) practices*

1. What names are given to your frontline staff whose primary role involves recruiting and consenting research participants into studies?

2. What key roles/activities do they undertake in your organization?

3. In your institution, how many staff does it have (in total) and how many of these are employed as frontline staff?

4. What is the range of qualifications for frontline staff and what is their minimum level of education/qualification?

5. What is the nature of their employment e.g. are they in contract? What is the average contract period? Are the contracts renewable and what is considered during renewal? Any systems that ensure retention of frontline staff in the institutions?

6. What systems are in place to coordinate the way frontline staff are employed and retained across different studies in the institutions? What is the role of PI (if any) in retaining frontline staff?

7. What formal and informal processes are in place to induct new frontline staff when they first arrive, and to train them for their roles?

---

[1] Manager is the person whose primary responsibility involves working directly with front line staff

For peer review only

**DISCUSSION GUIDE FOR RESEARCH INSTITUTIONS WORKING WITH FIELDWORKERS (FWs) IN SSA**

**TITLE: STRENGTHENING TRAINING OF FRONT LINE (FWs) STAFF AS A MEANS TO ENHANCE COMMUNITY ENGAGEMENT**

---

*8.* How is the training for interface staff organized? Are there any refresher trainings/skills improvement activities? What are the *training approach eg frequency and objectives of the training,*

*9.* How is the frontline staff training managed? Describe funding, design, whether centralized or decentralized, and monitoring and evaluation of the training

*10.* Describe any practices, experiences and studies (if any) that have informed the current training eg feedback mechanisms; availability of a community engagement strategy, community Knowledge Attitude and Perception (KAP) surveys etc

*11.* How do you ensure skills learned are assimilated into the frontline's day to day activities? Are there any support supervision mechanisms, performance appraisals etc ? How are these implemented?

*12.* Describe any career progression scheme used by your organization for these staff groups

*13.* What challenges do you experience in working with and managing frontline staff?

## SECTION C: Frontline staffs' Experience-Staff perspective

*This section explores frontline staff's practical and ethical challenges faced as they discharge their duties and their perceptions of the institutional support in training and CPD*

1. What are roles and responsibilities of frontline staff?

2. What challenges do frontline staff face when interacting with community members? *Practical and ethical challenges/dilemmas faced, e.g. issues of confidentiality?*

3. What type of in-house and external training is given to frontline staff in your institution?
   - ***Which of these do you perceive to be most appreciated and for what reason?***

4. What are the perceived institutional challenges in supporting and capacity building of frontline staff?
   - ***How do frontline members of staff address the above challenges?***

## SECTION D: Planned Workshop

At the end of this survey, we hope to invite a selected number of the institutions to a 2 day workshop. Kindly indicate if you would like to be considered to attend this workshop.

For peer review only

## COREQ (COnsolidated criteria for REporting Qualitative research) Checklist

A checklist of items that should be included in reports of qualitative research. You must report the page number in your manuscript where you consider each of the items listed in this checklist. If you have not included this information, either revise your manuscript accordingly before submitting or note N/A.

| Topic | Item No. | Guide Questions/Description | Reported on Page No. |
|---|---|---|---|
| **Domain 1: Research team and reflexivity** | | | |
| *Personal characteristics* | | | |
| Interviewer/facilitator | 1 | Which author/s conducted the interview or focus group? | 22 |
| Credentials | 2 | What were the researcher's credentials? E.g. PhD, MD | 22 |
| Occupation | 3 | What was their occupation at the time of the study? | 22 |
| Gender | 4 | Was the researcher male or female? | 22 |
| Experience and training | 5 | What experience or training did the researcher have? | 22 |
| *Relationship with participants* | | | |
| Relationship established | 6 | Was a relationship established prior to study commencement? | 6 |
| Participant knowledge of the interviewer | 7 | What did the participants know about the researcher? e.g. personal goals, reasons for doing the research | 6 |
| Interviewer characteristics | 8 | What characteristics were reported about the inter viewer/facilitator? e.g. Bias, assumptions, reasons and interests in the research topic | 3, 10, 22 |
| **Domain 2: Study design** | | | |
| *Theoretical framework* | | | |
| Methodological orientation and Theory | 9 | What methodological orientation was stated to underpin the study? e.g. grounded theory, discourse analysis, ethnography, phenomenology, content analysis | 5 |
| *Participant selection* | | | |
| Sampling | 10 | How were participants selected? e.g. purposive, convenience, consecutive, snowball | 5 |
| Method of approach | 11 | How were participants approached? e.g. face-to-face, telephone, mail, email | 5 |
| Sample size | 12 | How many participants were in the study? | 5 |
| Non-participation | 13 | How many people refused to participate or dropped out? Reasons? | 5 |
| *Setting* | | | |
| Setting of data collection | 14 | Where was the data collected? e.g. home, clinic, workplace | 8 |
| Presence of non-participants | 15 | Was anyone else present besides the participants and researchers? | 5 |
| Description of sample | 16 | What are the important characteristics of the sample? e.g. demographic data, date | 6 |
| *Data collection* | | | |
| Interview guide | 17 | Were questions, prompts, guides provided by the authors? Was it pilot tested? | 8 |
| Repeat interviews | 18 | Were repeat inter views carried out? If yes, how many? | 8-10 |
| Audio/visual recording | 19 | Did the research use audio or visual recording to collect the data? | 8 |
| Field notes | 20 | Were field notes made during and/or after the inter view or focus group? | 8 |
| Duration | 21 | What was the duration of the inter views or focus group? | 8 |
| Data saturation | 22 | Was data saturation discussed? | |
| Transcripts returned | 23 | Were transcripts returned to participants for comment and/or | |

For peer review only

For peer review only - http://bmjopen.bmj.com/site/about/guidelines.xhtml

| Topic | Item No. | Guide Questions/Description | Reported on Page No. |
|---|---|---|---|
| | | correction? | |
| **Domain 3: analysis and findings** | | | |
| *Data analysis* | | | |
| Number of data coders | 24 | How many data coders coded the data? | 9 |
| Description of the coding tree | 25 | Did authors provide a description of the coding tree? | |
| Derivation of themes | 26 | Were themes identified in advance or derived from the data? | 9-10 |
| Software | 27 | What software, if applicable, was used to manage the data? | 9 |
| Participant checking | 28 | Did participants provide feedback on the findings? | 9 |
| *Reporting* | | | |
| Quotations presented | 29 | Were participant quotations presented to illustrate the themes/findings? Was each quotation identified? e.g. participant number | 10-16 |
| Data and findings consistent | 30 | Was there consistency between the data presented and the findings? | |
| Clarity of major themes | 31 | Were major themes clearly presented in the findings? | 10-16 |
| Clarity of minor themes | 32 | Is there a description of diverse cases or discussion of minor themes? | 10-16 |

Developed from: Tong A, Sainsbury P, Craig J. Consolidated criteria for reporting qualitative research (COREQ): a 32-item checklist for interviews and focus groups. *International Journal for Quality in Health Care*. 2007. Volume 19, Number 6: pp. 349 – 357

**Once you have completed this checklist, please save a copy and upload it as part of your submission. DO NOT include this checklist as part of the main manuscript document. It must be uploaded as a separate file.**

For peer review only
